# The Monothiol Glutaredoxin Grx4 Regulates Iron Homeostasis and Virulence in Cryptococcus neoformans

**DOI:** 10.1128/mBio.02377-18

**Published:** 2018-12-04

**Authors:** Rodgoun Attarian, Guanggan Hu, Eddy Sánchez-León, Mélissa Caza, Daniel Croll, Eunsoo Do, Horacio Bach, Tricia Missall, Jennifer Lodge, Won Hee Jung, James W. Kronstad

**Affiliations:** a Michael Smith Laboratories, University of British Columbia, Vancouver, British Columbia, Canada; b Department of Microbiology and Immunology, University of British Columbia, Vancouver, British Columbia, Canada; c Laboratory of Evolutionary Genetics, Institute of Biology, University of Neuchâtel, Neuchâtel, Switzerland; d Department of Systems Biotechnology, Chung-Ang University, Anseong, South Korea; e Department of Biochemistry, Saint Louis University School of Medicine, St. Louis, Missouri, USA; f Department of Molecular Microbiology, Washington University School of Medicine, St. Louis, Missouri, USA; Duke University; University of Melbourne; Friedrich Schiller University Jena

**Keywords:** cryptococcosis, capsule, melanin, nuclear localization, transcriptome

## Abstract

The acquisition of iron and the maintenance of iron homeostasis are important aspects of virulence for the pathogenic fungus Cryptococcus neoformans. In this study, we characterized the role of the monothiol glutaredoxin Grx4 in iron homeostasis and virulence in C. neoformans. Monothiol glutaredoxins are important regulators of iron homeostasis because of their conserved roles in [2Fe-2S] cluster sensing and trafficking. We initially identified Grx4 as a binding partner of Cir1, a master regulator of iron-responsive genes and virulence factor elaboration in C. neoformans. We confirmed that Grx4 binds Cir1 and demonstrated that iron repletion promotes the relocalization of Grx4 from the nucleus to the cytoplasm. We also found that a *grx4* mutant lacking the GRX domain displayed iron-related phenotypes similar to those of a *cir1*Δ mutant, including poor growth upon iron deprivation. Importantly, the *grx4* mutant was avirulent in mice, a phenotype consistent with observed defects in the key virulence determinants, capsule and melanin, and poor growth at 37°C. A comparative transcriptome analysis of the *grx4* mutant and the WT strain under low-iron and iron-replete conditions confirmed a central role for Grx4 in iron homeostasis. Dysregulation of iron-related metabolism was consistent with *grx4* mutant phenotypes related to oxidative stress, mitochondrial function, and DNA repair. Overall, the phenotypes of the *grx4* mutant lacking the GRX domain and the transcriptome sequencing (RNA-Seq) analysis of the mutant support the hypothesis that Grx4 functions as an iron sensor, in part through an interaction with Cir1, to extensively regulate iron homeostasis.

## Abstract (precis)

Fungal pathogens cause life-threatening diseases in humans, particularly in immunocompromised people, and there is a tremendous need for a greater understanding of pathogenesis to support new therapies. One prominent fungal pathogen, Cryptococcus neoformans, causes meningitis in people suffering from HIV/AIDS. In the present study, we focused on characterizing mechanisms by which C. neoformans senses iron availability because iron is both a signal and a key nutrient for proliferation of the pathogen in vertebrate hosts. Specifically, we characterized a monothiol glutaredoxin protein, Grx4, that functions as a sensor of iron availability and interacts with regulatory factors to control the ability of C. neoformans to cause disease. Grx4 regulates key virulence factors, and a mutant is unable to cause disease in a mouse model of cryptococcosis. Overall, our study provides new insights into nutrient sensing and the role of iron in the pathogenesis of fungal diseases.

## INTRODUCTION

Cryptococcus neoformans is an opportunistic pathogen that causes life-threatening meningoencephalitis in immunocompromised people, including those with HIV/AIDS (1–3). Despite the use of highly active antiretroviral therapy (HAART), there are still ∼200,000 cases of cryptococcal meningoencephalitis per year, and the fungus is responsible for 15% of all AIDS-related deaths (4). This burden of disease underlines the urgent need to understand the mechanisms of fungus proliferation in vertebrate hosts as a foundation for identifying new drug and vaccine targets.

As with other pathogenic microbes, iron sensing and acquisition are important aspects of virulence for C. neoformans (5–8). Iron is important for C. neoformans both as a nutrient and as a signal to regulate the expression of the main virulence factor of the fungus, the polysaccharide capsule (5, 9). The abilities of C. neoformans to grow at the host body temperature of 37°C and to deposit melanin in the cell wall are also crucial for virulence (2, 6). To cause disease, the fungus must overcome nutritional immunity in which vertebrate hosts withhold iron to suppress pathogen growth (5, 10). C. neoformans employs various iron regulators and uptake mechanisms that contribute to virulence. These include heme uptake pathways as well as high- and low-affinity iron uptake systems (5, 11–13). The use of heme as an iron source depends on an exported mannoprotein, Cig1, and a cell surface reductase, Fre2 (13, 14). High-affinity uptake involves reduction of ferric iron (Fe^3+^) to the ferrous form (Fe^2+^) by cell surface reductases, with subsequent transport by a permease (Cft1) and ferroxidase (Cfo1) complex in the plasma membrane (5, 12, 14). The expression of these and other iron-related functions in C. neoformans is controlled by a GATA-type transcription factor, Cir1 (cryptococcal iron regulator 1), and additional transcription factors, including HapX (15, 16). Cir1 also integrates iron sensing and the regulation of iron uptake functions with the elaboration of virulence factors in C. neoformans (15).

Other fungi also use GATA-type transcriptional repressors with similarity to Cir1 to regulate the expression of iron-responsive genes; these fungi and their regulators include Schizosaccharomyces pombe (Fep1), Aspergillus sp. (SreA), Neurospora crassa (SRE), and Ustilago maydis (Urbs1) (17–21). In general, these GATA-type transcription factors are characterized by one or two zinc finger motifs for DNA binding, and these flank a region containing four conserved cysteine residues. In contrast, the regulators of iron homeostasis in Saccharomyces cerevisiae, Aft1 and Aft2, are transcriptional activators (22, 23).

The mechanisms by which iron-responsive transcription factors in fungi sense intracellular iron levels and regulate iron homeostasis are best understood in S. cerevisiae and S. pombe (22–26). In these fungi, the transcription factors interact with monothiol glutaredoxins (GRXs), which participate in iron sensing and regulation (19, 24, 27–35). Monothiol GRXs are glutathione (GSH)-dependent proteins with a cysteine-glycine-phenylalanine-serine (CGFS) motif at the active site. These proteins are found in both prokaryotes and eukaryotes and have emerged as key players in cellular redox and iron homeostasis (24, 36, 37). Recent studies in S. cerevisiae and S. pombe demonstrated essential roles for CGFS GRXs in intracellular iron homeostasis, iron trafficking, and the maturation of [2Fe-2S] cluster proteins and have established the proteins as novel [2Fe-2S] cluster-binding regulatory partners for transcription factors, including Fep1 and Aft1 (24, 25, 27–35).

In this study, we present evidence that the monothiol glutaredoxin Grx4 of C. neoformans is involved in virulence and the maintenance of iron homeostasis and that the protein interacts with the GATA-type iron regulator Cir1. Specifically, mutants lacking the *GRX4* region encoding the GRX domain are defective for growth at the host temperature of 37°C and upon iron limitation. Along with defects in other virulence factors such as capsule and melanin, these findings account for the loss of virulence for the *grx4* mutant in a murine model of cryptococcosis. Our results from transcriptional profiling by transcriptome sequencing (RNA-Seq) further support a role for the GRX domain of Grx4 in iron homeostasis through the regulation of functions for Fe-S cluster binding, heme biosynthesis, mitochondrial activities, and iron binding and uptake. These data support the conclusion that Grx4 is an important contributor to iron sensing and virulence in C. neoformans.

## RESULTS

### The monothiol glutaredoxin Grx4 interacts with Cir1, a regulator of functions for iron uptake and virulence.

Given that monothiol glutaredoxins (GRXs) make critical contributions to iron homeostasis in other fungi (24–27), we examined the genome sequence of the serotype A strain H99 of C. neoformans to identify candidate GRX proteins that could potentially interact with the key iron regulator Cir1. Specifically, we searched for orthologs of Grx3 and Grx4 from S. cerevisiae and Grx4 from S. pombe because these proteins are involved in iron regulation and homeostasis. A BLASTp analysis identified a putative monothiol glutaredoxin encoded by the gene CNAG_02950 in the genome of C. neoformans (38) that shared 63% amino acid sequence identity in the C-terminal region with Grx4 from S. pombe, 61% identity with Grx3 and Grx4 from S. cerevisiae, and 60% identity with Grx3 from Homo sapiens (see Fig. S1 in the supplemental material). The C-terminal region of Grx4 from C. neoformans and the other monothiol glutaredoxin proteins contains a conserved glutaredoxin (GRX) domain with a signature CGFS (cysteine glycine phenylalanine serine) motif in the predicted active site (Fig. S1). Of note, the monothiol Grx domain with the CGFS active site motif is highly conserved and known to be required for [2Fe-2S] cluster binding and trafficking, as well as the regulation of iron homeostasis in fungi (24–37). The N-terminal region of Grx4 from C. neoformans also contained the WAXXC motif of the thioredoxin (TRX) domain found in monothiol glutaredoxins (24, 36, 37). Overall, the sequence analysis supports a possible role for Grx4 in iron-related processes in C. neoformans.

10.1128/mBio.02377-18.1 FIG S1 Grx4 from C. neoformans has a conserved C-terminal GRX domain with a signature “CGFS” motif. (A) Alignment of the amino acid sequence of the C-terminal Grx domain of C. neoformans Grx4 (XP_012047837.1) with selected Grx sequences from other organisms. UhGrx4, Ustilago hordei, CCF50760.1; UmGrx4, Ustilago maydis, XP_011390702.1; SpGrx4, Schizosaccharomyces pombe, NP_596647.1; ScGrx3, Saccharomyces cerevisiae, AJV06961.1; ScGrx4, S. cerevisiae, NP_011101.3; CaGrx4, Candida albicans, KHC50815.1; HcGrx4, Histoplasma capsulatum, EEH02725.1; HsGrx4, Homo sapiens, AAF28841.1. (B) Phylogeny tree generated using neighbor-joining tree analysis in MEGA (v.7.0.26). Download FIG S1, PDF file, 0.8 MB. Copyright © 2018 Attarian et al. 2018 Attarian et al. This content is distributed under the terms of the Creative Commons Attribution 4.0 International license.

**FIG 1:**
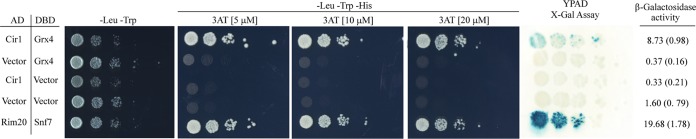
Grx4 interacts with Cir1. A yeast two-hybrid assay was used to examine the interaction of Grx4 and Cir1. DBD and AD indicate the Gal4 DNA binding and activation domains fused to Grx4 and Cir1, respectively. The vector designation indicates the empty vector control. All combinations of transformants grew in the absence of leucine (Leu) and tryptophan (Trp), confirming plasmid retention in the strains. Only yeast cells transformed with plasmids containing *GRX4* and *CIR1* grew in the absence of histidine (His), confirming an interaction to allow expression of *HIS3*. 3-Amino-1,2,4-triazole (3AT) was included at different concentrations to enhance the stringency of the *HIS3* selection. Qualitative and quantitative analyses of β-galactosidase activity were performed using X-Gal (5-bromo-4-chloro-3-indolyl-β-d-galactopyranoside) or chlorophenol red-β-d-galactopyranoside as a substrate, respectively, and the quantitative numbers represent the mean values of three assays with the standard error of the mean provided in parentheses.

Grx3 and Grx4 in S. cerevisiae and Grx4 in S. pombe are known to interact with and to influence the activity of transcription factors that regulate iron homeostasis (24–35). We therefore employed the yeast two-hybrid assay to test the interaction between Grx4 and Cir1. The gene encoding Grx4 was synthesized and fused to the coding region for the Gal4 DNA binding domain (DBD), and the cDNA for Cir1 was fused with the Gal4 activation domain (AD). Yeast cells that harbored both DBD-Grx4 and AD-Cir1 were selected based on growth in the absence of leucine and tryptophan. The ability of these transformants to grow without uracil or histidine and the expression of β-galactosidase indicated a positive interaction between Grx4 and Cir1 (Fig. 1). The previously established interaction of Snf7 and Rim20 (39) was included as a positive control (Fig. 1). Overall, the results support the conclusion that Grx4 and Cir1 interact.

### Grx4 colocalizes with Cir1 in the nucleus upon iron limitation, but partially relocates to the cytoplasm upon iron repletion.

**FIG 2:**
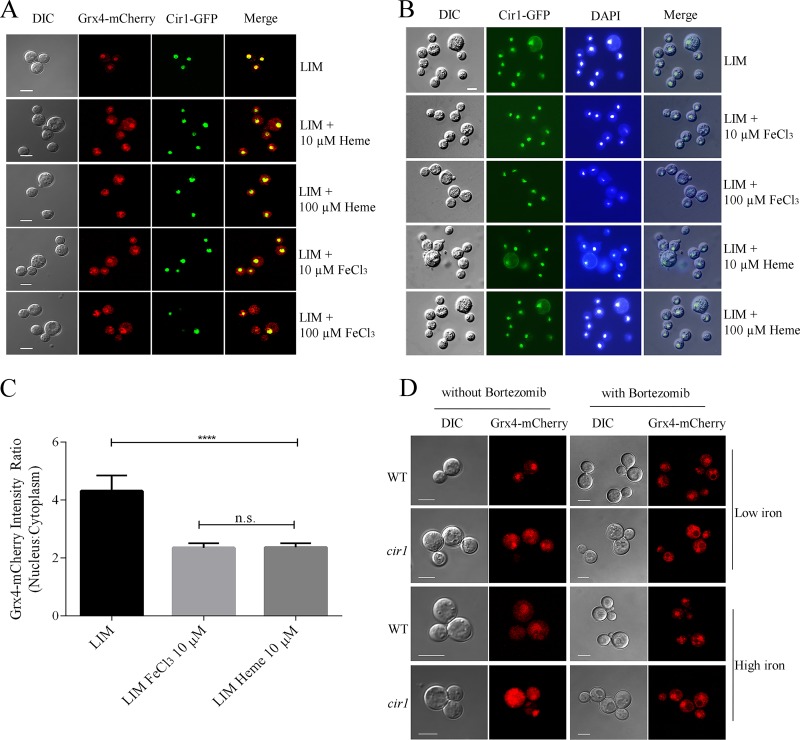
Grx4 is localized in nuclei upon iron limitation. (A) Grx4-mCherry and Cir1-GFP were colocalized in nuclei under low-iron conditions (defined low-iron medium [LIM]), but Grx4-mCherry shifted to the cytosol with addition of iron (FeCl_3_ or heme) and Cir1-GFP remained in the nucleus. Size bar = 5 μm. (B) Colocalization of Cir1-GFP with DAPI in nuclei under all conditions. (C) Quantitation of the Grx4-mCherry signal in the nucleus and cytoplasm in response to different levels of iron, as described in Materials and Methods. The graph shows the average of each treatment with 95% confidence intervals (CI; *n* ≥ 20). One-way ANOVA and Tukey statistical tests were performed to analyze the signal intensity, and *** indicates *P* < 0.0001. (D) Deletion of *CIR1* caused mislocalization of Grx4-mCherry to the cytosol under the low-iron condition (YNB + BPS medium). Grx4-mCherry was partially retained in nuclei under the high-iron condition upon treatment with bortezomib (BTZ), a proteasome inhibitor. The same results were obtained with cells grown in defined LIM. Size bar = 5 μm.

The ability of Grx4 and Cir1 to interact suggested that the proteins would colocalize in the nucleus, and we therefore examined protein localization by tagging Grx4 with mCherry and Cir1 with green fluorescent protein (GFP). Phenotypic assays indicated that the strains harboring the Cir1-GFP and Grx4-mCherry fusions behaved like the wild-type (WT) strain (see Fig. S2 in the supplemental material), and the Grx4-mCherry and Cir1-GFP fusion proteins were detected as single bands by immunoblot analysis (see Fig. S3 in the supplemental material). We cultured the strains carrying Grx4-mCherry and/or Cir1-GFP in low-iron or iron-replete medium for 5 h at 30°C and examined fluorescence. As shown in Fig. 2A, both proteins were found in the nucleus under the low-iron condition, and addition of iron as FeCl_3_ or heme resulted in partial relocalization of Grx4 to the cytoplasm (Fig. 2A). In contrast, Cir1 remained in the nucleus regardless of iron availability, and we confirmed the nuclear localization of Cir1-GFP by DAPI (4′,6-diamidino-2-phenylindole) staining (Fig. 2B). Quantitation of the relocation of Grx4-mCherry to the cytoplasm in response to iron and heme is shown in Fig. 2C. We observed that the ratio of the nuclear to cytoplasmic signals from Grx4-mCherry shifted from ∼4-fold to ∼2-fold upon iron/heme repletion. We also examined the dependence of Grx4-mCherry localization on Cir1 by expressing the protein in a *cir1* deletion mutant (Fig. 2D). In this situation, Grx4 was observed to be mainly in the cytoplasm, suggesting that the nuclear localization of Grx4 upon iron limitation is at least partially dependent on Cir1. An immunoblot analysis revealed that there was minimal decrease of protein levels for Grx4-mCherry in either the WT strain or *cir1* mutant after culturing the cells in yeast nitrogen base-bathophenanthroline disulfonate (YNB-BPS [low-iron condition]) or YNB-BPS plus FeCl_3_ for 5 h (see Fig. S3 in the supplemental material). As mentioned, Grx4-mCherry was distributed in the nucleus and cytoplasm in both WT and *cir1* mutant cells under the iron-replete condition (Fig. 2D). Interestingly, inclusion of the proteasome inhibitor bortezomib (BTZ) under the high-iron condition appeared to enhance the level of the Grx4-mCherry protein that remained in the nucleus in both the WT strain and the *cir1* mutant (Fig. 2D). Although additional analyses are needed to investigate this result, it is possible that a proteasome-sensitive factor participates in the proper localization of Grx4 along with Cir1. BTZ treatment did not influence the localization of the Cir1-GFP protein in the WT strain, regardless of iron availability (G. Hu, unpublished results). Overall, the localization experiments further support an interaction between Grx4 and Cir1 and revealed that Grx4 localization is influenced by iron availability, the iron regulator Cir1, and the proteasome inhibitor BTZ.

10.1128/mBio.02377-18.2 FIG S2 The phenotypes of tagged strains (Grx4-mCherry, Cir1-GFP, and Grx4-mCherry Cir1-GFP) are the same as the WT strain. Ten-fold serial dilutions of each strain grown in YPD medium overnight were spotted onto YPD or l-DOPA medium. The plates were incubated at either 30 or 37°C for 2**** ****days before being photographed. Download FIG S2, PDF file, 0.6 MB. Copyright © 2018 Attarian et al. 2018 Attarian et al. This content is distributed under the terms of the Creative Commons Attribution 4.0 International license.

10.1128/mBio.02377-18.3 FIG S3 Abundance of Cir1-GFP or Cir1-mCherry assessed by Western blot analysis. (A) Proteins from cells expressing Grx4-mCherry and WT cells grown under the low-iron condition were analyzed by Western blot analysis using anti-mCherry antibody. Intact Grx4-mCherry (58**** ****kDa) was detected in cells expressing Grx4-mCherry but not in WT cells. (B) Proteins from cells expressing Grx4-mCherry in either the WT strain or the *cir1* mutant grown under conditions in low- versus high-iron medium for 5 h were analyzed using anti-mCherry antibody. (C) The proteins from cells expressing Cir1-GFP in either the WT or *grx4* mutant background grown under the indicated conditions (low versus high iron) for 5 h were analyzed using anti-GFP antibody. Download FIG S3, PDF file, 0.9 MB. Copyright © 2018 Attarian et al. 2018 Attarian et al. This content is distributed under the terms of the Creative Commons Attribution 4.0 International license.

### Loss of the GRX domain in Grx4 results in defects in the formation of major virulence factors and blocks cryptococcosis in mice.

**FIG 3:**
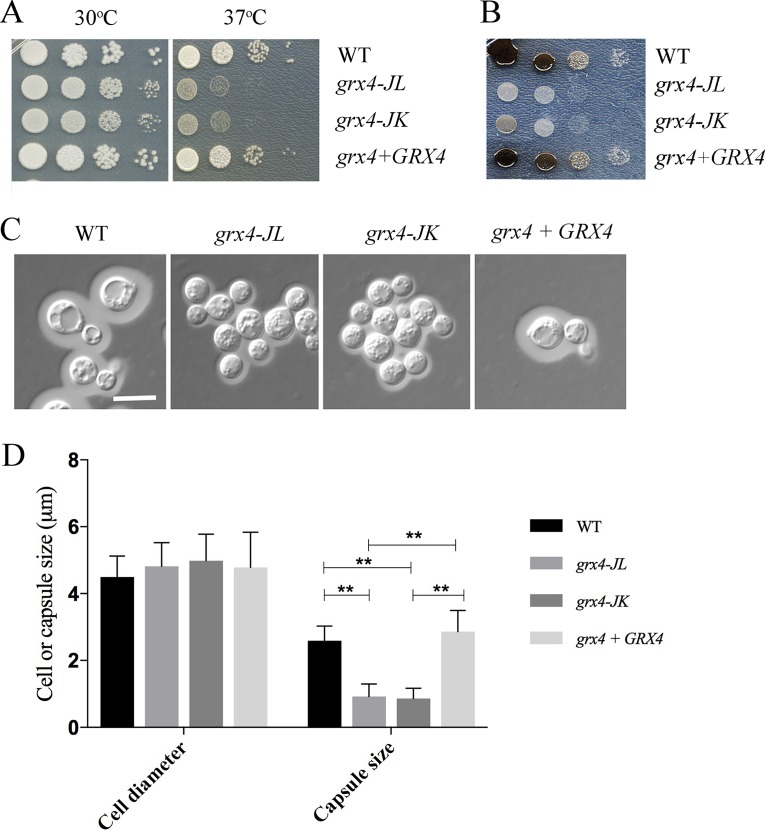
Grx4 influences the elaboration of three major virulence factors. (A) The sensitivity of the WT strain of C. neoformans, two independent *grx4* mutants, and the *GRX4* reconstituted strain to temperature (30 and 37°C) was examined using spot assays on YPD medium. (B) Spot assays were performed with each strain on l-DOPA plates with incubation at 30°C to examine melanin production. (C) Cells were grown in defined low-iron medium at 30°C for 48 h, and capsule formation was assessed by India ink staining for the indicated strains. Size bar = 10 μm. (D) Fifty cells of each strain from panel C were measured for cell diameter and capsule size. Each bar represents the average of the 50 measurements with standard deviations. Statistical significance relative to the WT capsule size is indicated by ** (Student's *t* test, *P* < 0.01).

We next investigated the function of Grx4 in C. neoformans by constructing a deletion mutation in the *GRX4* gene to remove the coding region for the GRX domain. Specifically, two independent mutants were constructed that lack the sequence encoding the C-terminal 157-amino-acid region containing the GRX domain. These mutations are designated *grx4-JL* and *grx4-JK*, as described in Materials and Methods. Interestingly, we were unable to delete the entire open reading frame, perhaps due to an essential function for the N-terminal TRX domain or an impact on an adjacent gene. We note that the deletion mutant retains the first two exons that would potentially encode an N-terminal 85 amino acid segment with part of the TRX domain, as identified by structural analysis for Grx3 in S. cerevisiae (40). We also complemented the *grx4-JL* mutation with the WT *GRX4* gene. The strains were initially examined for the phenotypes related to virulence that were previously observed in mutants lacking Cir1 (15). Specifically, we found that the *grx4-JL* and *grx4-JK* mutants displayed poor growth at 37°C (Fig. 3A), a phenotype shared with the *cir1* mutant (15). The *grx4* mutants also had reduced production of melanin on medium containing l-DOPA (l-3,4-dihydroxyphenylalanine) as a substrate (Fig. 3B), and this was in contrast to the melanin production observed for the *cir1* mutant (15). The polysaccharide capsule is a major virulence trait for C. neoformans, and the loss of Grx4 also resulted in reduced capsule size (Fig. 3C and D), as does loss of Cir1 (15). Taken together, these findings indicate that the GRX domain of Grx4 is an important regulator of virulence factor production in C. neoformans.

**FIG 4:**
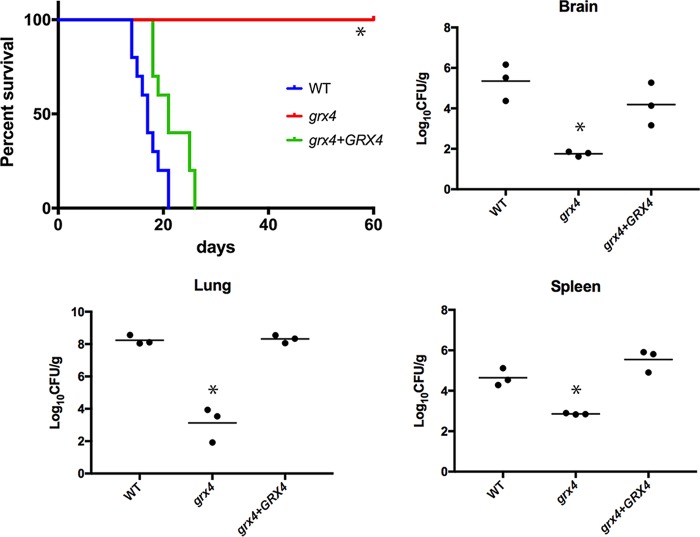
Grx4 is required for virulence in a mouse inhalation model. Ten female BALB/c mice were challenged by intranasal inoculation with 10^5^ cells of the WT strain (H99), the *grx4-JL* mutant, or the *GRX4* reconstituted strain. Survival differences between groups of mice were evaluated by the log rank Mantel-Cox test. The *P* values for the mice infected with the WT and mutant strains were statistically different (*, *P* <0.001). Also shown is the distribution of fungal cells in the organs (brain, lung, and spleen) of infected mice. Organs infected with the WT, the *grx4-JL* mutant, or the *GRX4* reconstituted strain were collected at the humane endpoint of the experiment, and fungal burdens were monitored in organs by determining CFU upon plating on YPD medium. Three mice for each strain were used for the experiments, and horizontal bars in each graph represent the average CFU. In all organs, differences in fungal burdens between the *grx4* mutant and the WT strain and between the *grx4* mutant and the reconstituted strain, were statistically significant by the nonparametric Mann-Whitney two-tailed U test (*, *P* < 0.05).

Our analysis of the impact of Grx4 on virulence-related phenotypes predicted that *grx4* mutants would be unable to cause disease in mice. To test this idea, we inoculated mice intranasally with cells of the WT strain, the *grx4-JL* mutant, or the complemented strain. All mice infected with the WT and complemented cells succumbed to infection by day 24, while the mice infected with the *grx4-JL* mutant did not show disease symptoms and survived for the duration of the experiment (60 days) (Fig. 4). A more detailed examination of fungal burden in the infected mice revealed that the *grx4-JL* mutant failed to accumulate in the brain, lung, and spleen (Fig. 4). Therefore, we conclude that the GRX domain of Grx4 is required for the proliferation and/or survival of C. neoformans in a vertebrate host.

### The GRX domain of Grx4 is required for growth on low-iron media.

**FIG 5:**
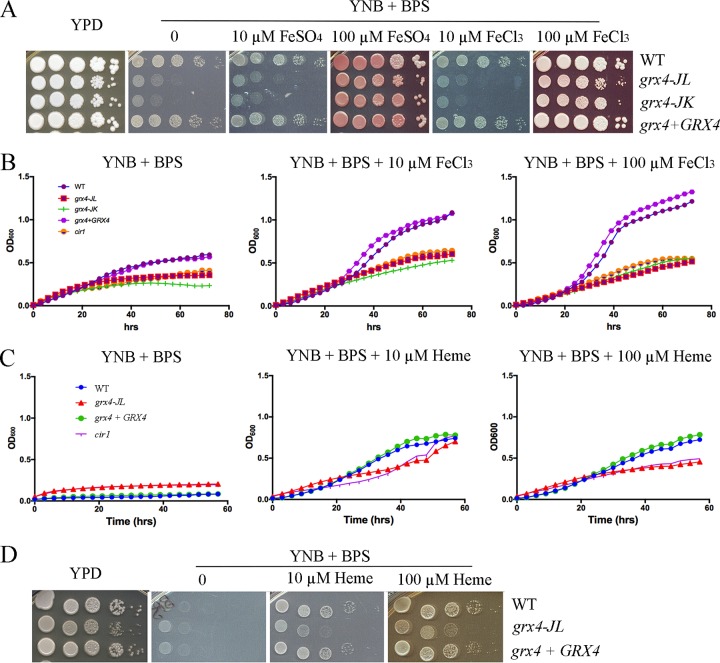
Grx4 is required for robust growth on inorganic iron or heme as the sole iron source. (A) Spot assays with each strain were performed on YNB-BPS medium with different concentrations of FeSO_4_ or FeCl_3._ (B) Cells of the WT, the *grx4* mutant, and the *GRX4* reconstituted strain were inoculated into liquid YNB medium plus 150 μM BPS without and with supplementation with FeCl_3_ as the iron source. The cultures were incubated at 30°C, and OD_600_ values were measured. The *cir1* mutant strain was included for comparison with the *grx4* strains. (C) The indicated strains were also tested for growth without and with supplementation with heme by the same method as in panel B. (D) Spot assays of each strain on YNB-BPS medium with different concentrations of heme.

**FIG 6:**
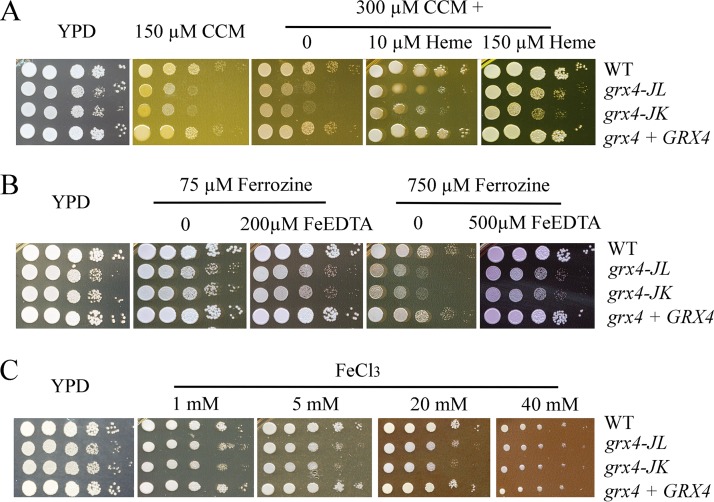
Grx4 is involved in iron homeostasis. (A and B) Disruption of *GRX4* leads to increased sensitivity to the iron-chelating drugs curcumin and ferrozine. (A) Spot assays with the WT, two independent *grx4* mutants, and the *GRX4* complemented strains (without iron starvation) on YPD plates with or without curcumin (CCM) at a concentration of either 150 or 300 μM, supplemented with 0, 10, or 150 μM heme as the iron source. (B) Spot assays with each strain without iron starvation on YPD plates with or without 75 μM or 750 μM ferrozine supplemented with 0 or the indicated amount of FeEDTA (15). (C) Spot assays with each strain grown in YPD medium overnight and spotted onto YPD supplemented with either 1, 5, 20, or 40 mM FeCl_3_.

Given that Grx4 is critical for the virulence of C. neoformans and shares some phenotypes with Cir1, we hypothesized that the GRX domain of Grx4 might also contribute along with Cir1 to iron homeostasis. We therefore examined the ability of the *grx4* mutants to proliferate on media with low and high concentrations of iron and heme. We found that the mutants showed poor proliferation on solid media with FeCl_3_, FeSO_4_, or heme as iron sources (Fig. 5). The impaired growth was particularly notable at low iron levels where the cells are dependent on high-affinity iron uptake. Similar defects in proliferation for the mutants were observed for cultures in liquid media with reduced iron availability (Fig. 5). We also examined the influence of iron on the *grx4* mutants in more detail by testing their sensitivity to iron chelators (e.g., curcumin and ferrozine), as well as their ability to proliferate in the presence of elevated iron concentrations (Fig. 6A to C). These experiments revealed that the mutants were sensitive to curcumin and high concentrations of ferrozine. Heme and FeEDTA partially rescued the inhibitory effects of curcumin or ferrozine, respectively (Fig. 6A and B). Elevated iron in the culture medium slightly impaired the proliferation of the mutants (Fig. 6C). Overall, these results indicate that the GRX domain of Grx4 participates in iron homeostasis in C. neoformans, a role that is consistent with its interaction with Cir1.

### Transcriptional profiling supports a role for the GRX domain of Grx4 in the regulation of iron-dependent processes.

**FIG 7:**
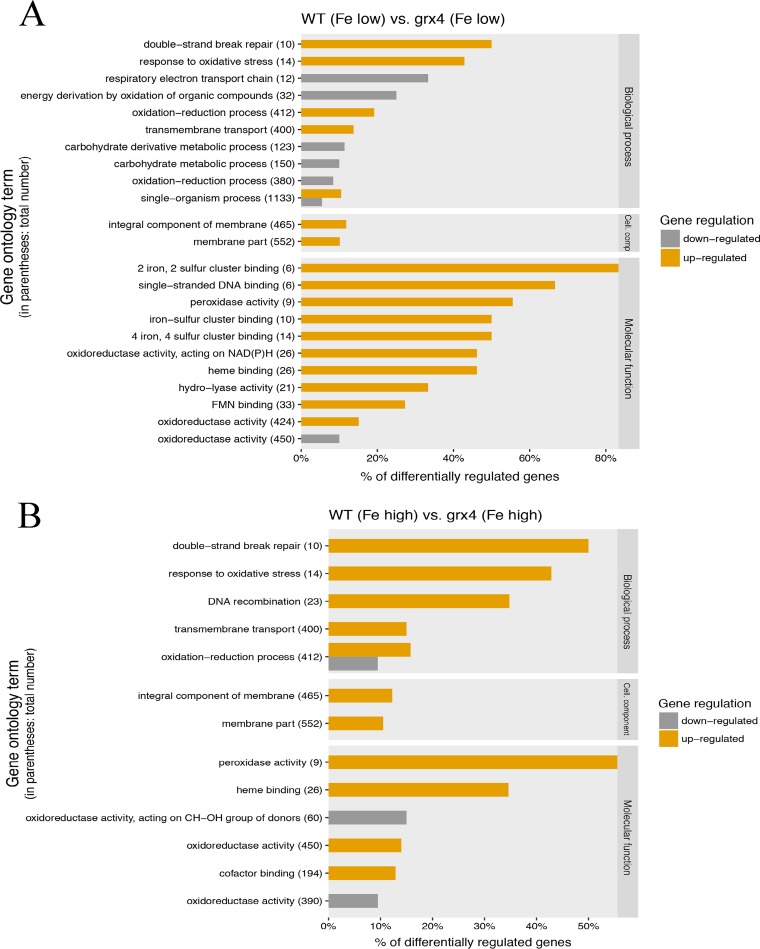
Impact of loss of the GRX domain of Grx4 on the transcripts for specific Gene Ontology categories. (A and B) Gene Ontology (GO) enrichment analysis of the differentially expressed genes between WT and *grx4* strains under low-iron (A) and high-iron (B) conditions (with total gene numbers within each functional category shown as the percentage of genes showing differential expression).

The glutaredoxin Grx4 in S. pombe is known to coregulate genes for iron acquisition with Fep1, an ortholog of Cir1, and to interact with Php4, a regulator of functions that use iron (27, 29, 30, 32–35). We therefore performed an RNA-Seq analysis to assess the impact of loss of the GRX domain of Grx4 on the transcriptomes of C. neoformans cells grown under low- and high-iron conditions (Fig. 7). As shown in Table 1, the deletion mutation in *GRX4* had an impact on transcript levels for several hundred genes under both low- and high-iron conditions. That is, ∼647 genes were upregulated in the *grx4* mutant compared with the WT strain under the low-iron condition, and 736 genes were upregulated under the high-iron condition. We also found that 404 genes were downregulated in the *grx4* mutant under the low-iron condition versus 358 genes under the high-iron condition (Table 1). The lists of upregulated or downregulated transcripts under both conditions are presented in Tables S1 and S2, respectively, in the supplemental material.

**TABLE 1:** Number of genes differentially regulated by iron availability and/or Grx4

Comparison	No. of genes^*a*^:							
	Upregulated				Downregulated			
	2-fold	5-fold	10-fold	Total	2-fold	5-fold	10-fold	Total
WT: low vs high iron	525	16	0	541	328	53	27	408
*grx4* vs WT								
Low iron	498	99	50	647	317	62	25	404
High iron	580	100	56	736	290	52	16	358
*grx4*: low vs high iron	100	2	0	102	237	11	0	248

a The gene numbers were calculated using *P* value-based statistics (*P* < 0.01).

10.1128/mBio.02377-18.7 TABLE S1 List of transcripts upregulated in the *grx4* mutant under both low- and high-iron conditions. Download Table S1, DOCX file, 0.1 MB. Copyright © 2018 Attarian et al. 2018 Attarian et al. This content is distributed under the terms of the Creative Commons Attribution 4.0 International license.

10.1128/mBio.02377-18.8 TABLE S2 List of transcripts downregulated in the *grx4* mutant under both low- and high-iron conditions. Download Table S2, DOCX file, 0.08 MB. Copyright © 2018 Attarian et al. 2018 Attarian et al. This content is distributed under the terms of the Creative Commons Attribution 4.0 International license.

An analysis of Gene Ontology (GO) terms for biological processes revealed that the highest percentages of differentially expressed and upregulated genes (top 3) under the low-iron condition were in double-strand break repair, response to oxidative stress, and oxidation-reduction processes (Fig. 7A). Respiratory electron transport chain, energy derivation by oxidation of organic compounds, and carbohydrate derivative metabolic process were the top categories for genes with downregulated transcripts (Fig. 7A). For the iron-replete condition, the top categories for upregulated transcripts were double-strand break repair, response to oxidative stress, and DNA recombination, while the single category noted for downregulated transcripts was oxidation-reduction process (Fig. 7B). Notable GO terms for molecular function also implicated Grx4 in the regulation of [2Fe-2S] cluster binding, single-stranded DNA binding, peroxidase activity, oxidoreductase activity, and heme binding (Fig. 7B). The influence of Grx4 on DNA-related processes and iron-dependent mitochondrial functions related to respiration and oxidative phosphorylation was also highlighted by STRING analysis (41) (see Fig. S5 in the supplemental material). As a whole, these results, along with the interaction of Grx4 with the iron regulator Cir1 (Fig. 1 and 2) and the influence of the *grx4* deletion on growth on low-iron media (Fig. 5), highlight the important role that the GRX domain of Grx4 plays in iron-related processes in C. neoformans.

10.1128/mBio.02377-18.4 FIG S4 STRING analysis of upregulated transcripts in the *grx4* cells under either the low- or high-iron condition. STRING was used to visualize predicted protein-protein interactions for the identified 639 Grx4-regulated proteins (http://string-db.org) using the corresponding proteins from C. neoformans strain JEC21 in the database. Notably, functions relevant to mitochondria (cellular respiration, electron transport, and metal binding) and oxidative phosphorylation were identified. Download FIG S4, PDF file, 2.2 MB. Copyright © 2018 Attarian et al. 2018 Attarian et al. This content is distributed under the terms of the Creative Commons Attribution 4.0 International license.

10.1128/mBio.02377-18.5 FIG S5 Verification of RNA-Seq data by qPCR. qPCR was used to verify the transcript levels of *CIR1*, *LAC1*, and *FRE3* in the WT and *grx4* mutant cells, grown under either the low-iron (A) or high-iron (B) condition. The expression pattern of these three genes is consistent with the data revealed by RNA-Seq analysis. Download FIG S5, PDF file, 0.6 MB. Copyright © 2018 Attarian et al. 2018 Attarian et al. This content is distributed under the terms of the Creative Commons Attribution 4.0 International license.

We also manually examined the categories of genes whose transcript levels are influenced by Grx4 and compiled a table to illustrate the regulation of selected functions for iron transport, heme biosynthesis, mitochondrial and cytosolic iron sulfur cluster assembly, iron sulfur-containing proteins, electron transport, mitochondrial functions, DNA repair, ergosterol metabolism, and oxidative stress (Table 2). We also performed quantitative reverse transcription-PCR (qRT-PCR) to compare the expression of genes encoding the functions related to iron (*CIR1*, *LAC1*, and *FRE3*) in WT and *grx4* mutant cells and found results consistent with the RNA-Seq data (Fig. S5). In general, these results highlighted the negative impact of Grx4 on the regulation of iron-dependent functions (Table 2), similar to the influence of the monothiol glutaredoxin Grx4 with the transcription factor Php4 in S. pombe (27, 32–35).

**TABLE 2:** Grx4-regulated genes encoding iron transport and homeostasis and mitochondrial functions

			Result for iron level comparison^*a*^:			
Gene ID	Gene name	Function	WT low vs high	*grx4* low vs WT low	*grx4* high vs WT high	*grx4* low vs high
Iron transport						
CNAG_00815	*SIT1*	Siderochrome-iron uptake transporter	1.86	1.06	**2.09**	0.94
CNAG_06761	*SIT3*	Siderophore-iron transporter Str1	**2.29**	*0.37*	0.89	0.96
CNAG_07387	*SIT4*	Siderophore-iron transporter	**2.81**	**3.92**	**10.55**	1.04
CNAG_07519	*SIT5*	Siderophore-iron transporter	**2.23**	0.64	**2.07**	0.68
CNAG_07751	*SIT6*	Siderophore iron transporter MirB	**3.63**	**2.32**	**8.35**	1.00
CNAG_03498	*FRE201*	Metalloreductase	1.45	**2.06**	**3.39**	0.88
CNAG_06524	*FRE3*	Ferric reductase	**2.50**	**5.07**	**16.25**	0.77
CNAG_06976	*FRE6*	Ferric reductase	**2.40**	0.66	1.16	1.35
CNAG_00876	*FRE7*	Ferric reductase	*0.40*	0.89	*0.49*	0.72
CNAG_03498	*FRE8*	Ferric reductase	1.45	**2.06**	**3.39**	0.88
CNAG_04864	*CIR1*	Iron regulator 1	0.81	*0.02*	*0.01*	1.23
CNAG_01242	*HAPX*	Conserved hypothetical protein	1.93	1.21	**2.14**	1.08
CNAG_02950	*GRX4*	Grx4 family monothiol glutaredoxin	1.72	*0.11*	*0.15*	1.21
CNAG_00727	*MMT2*	Mitochondrial protein with role in iron accumulation	0.50	0.89	*0.43*	1.04
CNAG_03465	*LAC1*	Laccase 1	0.99	**10.22**	**7.55**	1.32
CNAG_05154	*CCC1*	Membrane fraction protein	0.57	**5.20**	**5.50**	0.54
CNAG_07519	*SIT1*/*ARN1*	Conserved hypothetical protein	**2.23**	0.64	**2.07**	0.68
CNAG_01653	*CIG1*	Cytokine inducing-glycoprotein, putative a hemophore	**2.29**	0.64	1.32	1.10
CNAG_07865		Ferro-O_2_-oxidoreductase	**2.55**	0.94	1.99	1.20
Heme biosynthesis						
CNAG_01721	*HEM3*	Porphobilinogen deaminase	*0.15*	**2.62**	1.71	*0.22*
CNAG_02460	*HEM13*	Coproporphyrinogen III oxidase	*0.39*	0.82	*0.27*	1.16
CNAG_03939	*HEM1*	5-Aminolevulinic acid synthase	*0.19*	**2.52**	1.10	*0.42*
CNAG_03187	*HEM14*	Protoporphyrinogen oxidase	0.92	*0.45*	*0.45*	0.91
CNAG_01908	*HEM4*	Uroporphyrinogen-III synthase	*0.04*	**10.60**	**2.72**	*0.14*
Mitochondrial ISC assembly						
CNAG_03395	*NFU1*	NifU-like protein C	*0.10*	**3.78**	1.59	*0.25*
CNAG_03589	*YAH1*	Adrenodoxin-type ferredoxin	0.54	**2.24**	**2.22**	0.54
CNAG_02522	*MRS3*/*4*	Carrier	1.25	0.85	1.11	0.95
CNAG_03985	*GRX5*	Monothiol glutaredoxin-5	*0.45*	1.95	**2.04**	*0.43*
CNAG_05199	*SSQ1*	Heat shock protein	*0.41*	0.81	0.52	0.64
CNAG_04288	*JAC1*	Conserved hypothetical protein	1.78	*0.34*	0.60	1.01
CNAG_02131	*ISA1*	Iron sulfur assembly protein 1	*0.32*	**2.20**	1.58	*0.44*
CNAG_00491	*ISA2*	Iron sulfur assembly protein 2	0.92	0.70	0.82	0.77
CNAG_00389	*IBA57*	Mitochondrial protein	*0.25*	**2.42**	1.17	0.52
Cytosolic ISC assembly						
CNAG_04202	*NAR1*	Iron hydrogenase	*0.15*	**3.04**	1.61	*0.28*
CNAG_01802	*DRE2*	Cytoplasmic protein	*0.50*	**3.10**	**2.41**	0.63
CNAG_01137	*ACO1*	Aconitase	*0.21*	1.47	1.35	*0.23*
ISC-containing proteins						
CNAG_07908	*ACO2*	Aconitate hydratase	*0.20*	**5.64**	**2.13**	0.53
CNAG_06621	*BIO2*	Biotin synthase	*0.35*	1.28	0.98	*0.45*
CNAG_00462	*CIR2*	Oxidoreductase	*0.05*	**8.51**	1.35	*0.29*
CNAG_01914	*COQ11*	Mitochondrial protein	0.98	0.58	0.56	1.00
CNAG_07572	*ELP3*	Pol II transcription elongation factor	*0.27*	**2.90**	1.76	*0.44*
CNAG_04862	*GLT1*	Glutamate synthase	*0.22*	**7.36**	**7.15**	*0.22*
CNAG_07491	*GRX6*	Conserved hypothetical protein	1.06	0.71	0.92	0.82
CNAG_00237	*LEU1*	3-Isopropylmalate dehydratase	*0.22*	**9.54**	**3.01**	0.70
CNAG_05194	*LIP5*	Lipoic acid synthetase	*0.47*	1.89	**2.16**	*0.41*
CNAG_02565	*LYS4*	Homoaconitate hydratase	*0.07*	**8.81**	1.64	*0.40*
CNAG_05070	*MET5*	Sulfite reductase beta subunit	*0.14*	**3.60**	**2.13**	*0.23*
CNAG_03206	*NTG2*	DNA-(apurinic or apyrimidinic site) lyase	*0.23*	**3.50**	1.89	*0.42*
CNAG_02315	*RIP1*	Ubiquinol-cytochrome *c* reductase iron-sulfur subunit	*0.22*	**2.47**	1.30	*0.42*
CNAG_07667	*SAT4*	Other/HAL protein kinase	0.89	0.92	0.83	0.98
CNAG_07356	*SHH3*	Succinate dehydrogenase	*0.16*	**2.19**	1.13	*0.32*
CNAG_06558	*TAH18*	NADPH-ferrihemoprotein reductase	0.81	**3.20**	1.82	1.41
Electron transport						
CNAG_05318	*CYB2*	l-Mandelate dehydrogenase	*0.41*	**2.78**	1.84	0.61
CNAG_05169	*CYB2*	Cytochrome *b*_2_	*0.21*	**4.54**	1.86	*0.50*
CNAG_07480	*MCR1*	Cytochrome *b*_5_ reductase	**2.24**	0.95	1.67	1.27
CNAG_00716	*CYC7*	Electron carrier	*0.22*	**2.14**	1.96	*0.23*
CNAG_03666		Acyl-CoA dehydrogenase	*0.06*	**2.83**	1.16	*0.14*
CNAG_03629	*YHB1*	NADH-ubiquinone oxidoreductase	*0.05*	**4.30**	1.15	*0.20*
CNAG_01229	*CYB2*	l-Mandelate dehydrogenase	*0.13*	**3.91**	**2.05**	*0.25*
CNAG_05631		NADH-ubiquinone oxidoreductase	*0.07*	**5.40**	1.29	*0.30*
CNAG_01078	*ALD5*	Aldehyde dehydrogenase	1.51	**2.39**	**3.00**	1.20
CNAG_04189	*SDH1*	Succinate dehydrogenase flavoprotein subunit	*0.17*	1.52	1.03	*0.24*
CNAG_04521		Oxidoreductase	1.40	1.83	**2.54**	1.00
CNAG_03663	*CYB2*	l-Lactate dehydrogenase	*0.10*	**3.02**	1.31	*0.24*
CNAG_03226	*SDH2*	Succinate dehydrogenase iron-sulfur subunit	*0.09*	1.48	0.87	*0.15*
CNAG_05179	*QCR2*	Ubiquinol-cytochrome *c* reductase complex core	*0.17*	**2.12**	0.95	*0.37*
CNAG_05258		Glucose-methanol-choline oxidoreductase	**5.68**	**4.72**	**17.36**	1.53
CNAG_01323	*QCR7*	Ubiquinol-cytochrome *c* reductase	*0.27*	**3.20**	1.96	*0.43*
CNAG_05909	*CYT1*	Electron transporter	*0.12*	**3.18**	1.18	*0.32*
Mitochondrial functions						
CNAG_00162		Alternative oxidase	*0.02*	**52.56**	1.80	0.68
CNAG_03824	*MIR1*	Phosphate transport protein MIR1	1.19	*0.26*	*0.31*	1.00
CNAG_00499		Carnitine/acyl carnitine carrier	*0.36*	1.70	1.04	0.59
CNAG_05909		Electron transporter	*0.12*	**3.18**	1.18	*0.32*
CNAG_02315		Ubiquinol-cytochrome *c* reductase	*0.22*	**2.47**	1.30	*0.42*
CNAG_05132		Cytochrome *c* oxidase	*0.48*	1.14	0.95	0.56
CNAG_03225		Malate dehydrogenase	0.91	0.56	*0.44*	1.14
CNAG_00237		3-Isopropylmalate dehydratase	*0.22*	**9.54**	**3.01**	0.70
CNAG_03596		Dihydrolipoamide succinyltransferase	*0.46*	0.87	0.74	0.54
CNAG_07908		Aconitate hydratase	*0.20*	**5.64**	**2.13**	0.53
CNAG_05031		Succinyl-CoA:3-ketoacid-coenzyme A transferase	0.96	*0.22*	0.65	*0.32*
CNAG_06138		NADH dehydrogenase (ubiquinone) Fe-S 6	*0.22*	**2.05**	1.01	*0.45*
DNA repair						
CNAG_07552	*RAD8*	DNA repair Rad8	0.87	**6.75**	**4.97**	1.17
CNAG_00178	*REV1*	DNA repair REV1	1.40	**6.12**	**7.12**	1.19
CNAG_05198	*RAD7*	DNA repair RAD7	1.27	**5.49**	**7.27**	0.95
CNAG_02544	*SWI5*	DNA repair Swi5/Sae3	1.56	**5.06**	**7.04**	1.10
CNAG_02512	*RAD16*	DNA repair RAD16	1.97	**4.84**	**8.86**	1.06
CNAG_01163	*RAD54*	DNA repair and recombination RAD54	1.24	**3.42**	**3.98**	1.06
CNAG_00299	*RAD5*	DNA repair RAD5	0.75	**2.82**	**2.19**	0.96
CNAG_02771	*RAD54b*	DNA repair and recombination RAD54B	1.10	**7.91**	**8.79**	0.98
CNAG_00720	*RAD51*	DNA repair RAD51	1.51	**7.11**	**10.72**	0.99
CNAG_03637		Double-strand break repair factor and silencing regulator	1.09	**3.61**	**3.71**	1.05
Ergosterol metabolism						
CNAG_00519	*ERG3*		**2.18**	*0.24*	*0.39*	1.30
CNAG_01129	*ERG7*		1.66	*0.43*	0.54	1.32
CNAG_00854	*ERG2*		**2.46**	*0.25*	0.57	1.08
CNAG_02830	*ERG4*		1.82	*0.46*	0.94	0.88
CNAG_01737	*ERG25*		**2.19**	*0.17*	*0.35*	1.06
Oxidative stress						
CNAG_00575	*CAT3*	Catalase 3 (CAT3)	*0.48*	**5.59**	**3.31**	0.81
CNAG_00654	*SRX1*	Conserved hypothetical protein (SRX1, sulfiredoxin)	**4.34**	**4.85**	**14.41**	1.45
CNAG_01138	*CCP1*	Cytochrome *c* peroxidase (CCP1)	*0.07*	**181.00**	**27.35**	*0.44*
CNAG_03482	*TSA1*	Thioredoxin-dependent peroxide reductase (TSA1)	1.31	**5.10**	**4.62**	1.44
CNAG_03985	*GRX5*	Monothiol glutaredoxin-5 (GRX5)	*0.45*	1.95	**2.04**	*0.43*
CNAG_04388	*SOD2*	Mitochondrial superoxide dismutase Sod2	0.98	0.58	0.51	1.12
CNAG_05847	*TRR1*	Thioredoxin-disulfide reductase (TRR1)	1.24	**12.12**	**10.13**	1.47

a The numbers in boldface are the measurements where the log_2_ value is ≥2; the numbers in italic are log_2_ values of <0.5.

**FIG 8:**
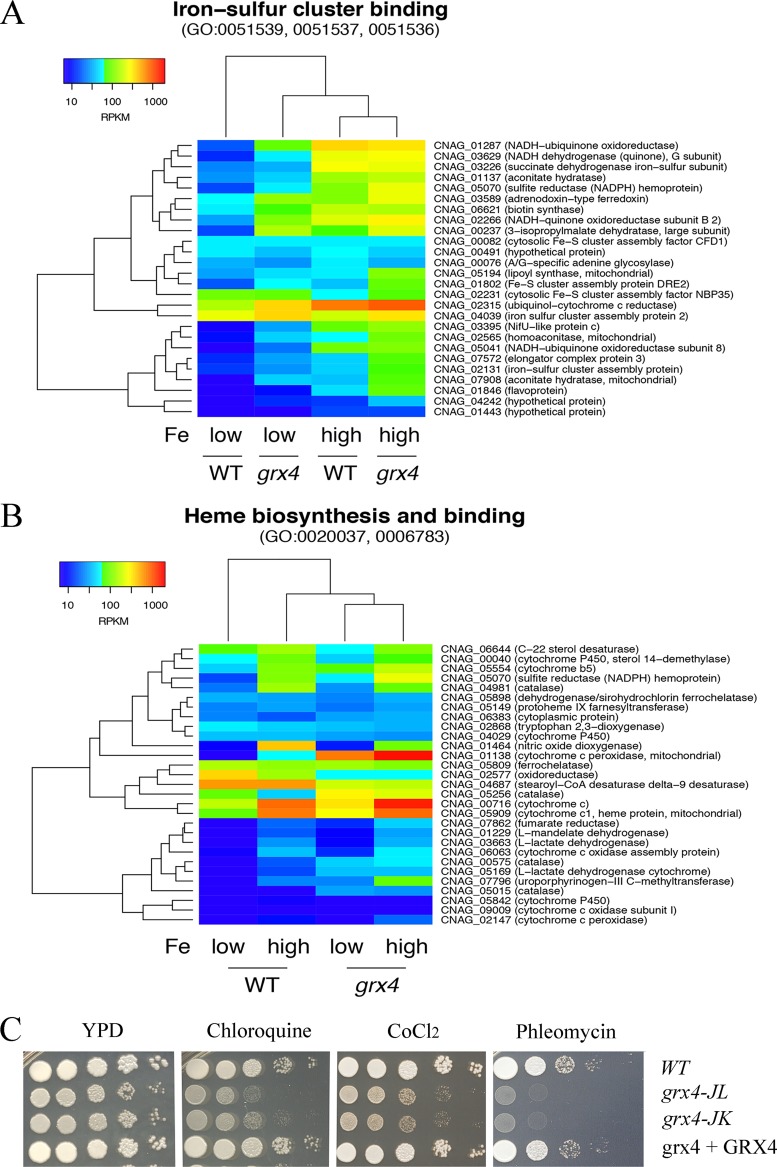
Grx4 regulates genes involved in metal ion transport, heme biosynthesis, and iron-sulfur cluster binding in response to iron availability. (A) Changes in transcript abundance of the genes encoding functions in iron-sulfur cluster binding between the WT and *grx4* mutant strains grown under low- and high-iron conditions, with the results represented by the heat map. (B) Changes in transcript abundance of the genes encoding functions in heme biosynthesis and binding between the WT and *grx4* mutant strains grown under low- and high-iron conditions, with the results represented by the heat map. (C) Spot assays of the indicated strains on YPD medium with or without the antimalarial drug chloroquine (6 mM), CoCl_2_ (a hypoxia-mimicking agent [600 μM]), or phleomycin (an iron-dependent inhibitor [8 μg/ml]).

**FIG 9:**
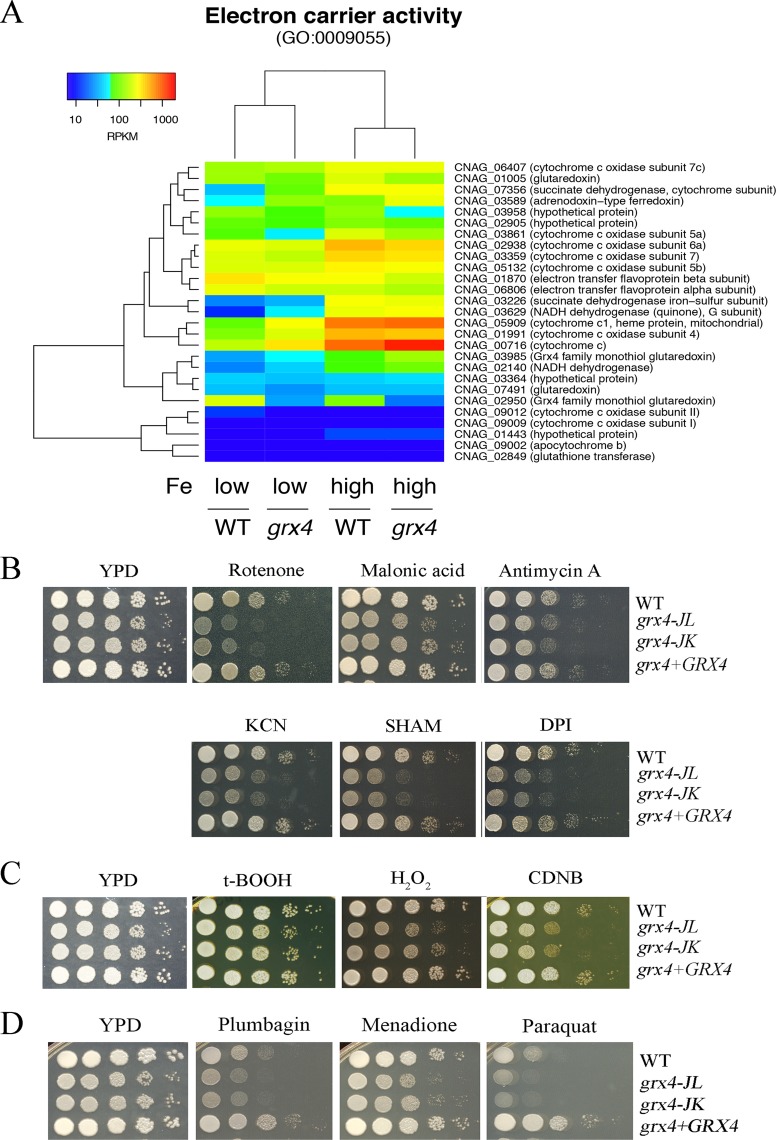
Grx4 is implicated in the regulation of functions for electron transport and the response to oxidative stress. (A) Heat map representation of changes in transcript abundance of the genes encoding functions in electron carrier activity between the WT and *grx4* mutant strains grown under low- and high-iron conditions. (B) Spot assays on YPD medium indicate that the *grx4* mutation leads to sensitivity to inhibitors of electron transport chain complexes I to IV and the alternative oxidase (75 μg/ml rotenone, 2 mM malonic acid, 5 μg/ml antimycin A, 10 mM potassium cyanide [KCN], 10 mM salicylic hydroxamate [SHAM], and 50 μM diphenyleneiodonium [DPI]). (C) Spot assays on YPD medium indicate that the *grx4* mutation of *GRX4* leads to the sensitivity to agents that provoke oxidative stress (2 mM t-BOOH, 0.01% H_2_O_2_, and 5 µg/ml 1-chloro-2,4-dinitrobenzene [CDNB]). (D) Spot assays on YPD medium indicate that the *grx4* mutants have altered susceptibilities to inhibitors of reactive oxygen species (50 µM plumbagin, 5 µg/ml menadione, and 500 µM paraquat).

**FIG 10:**
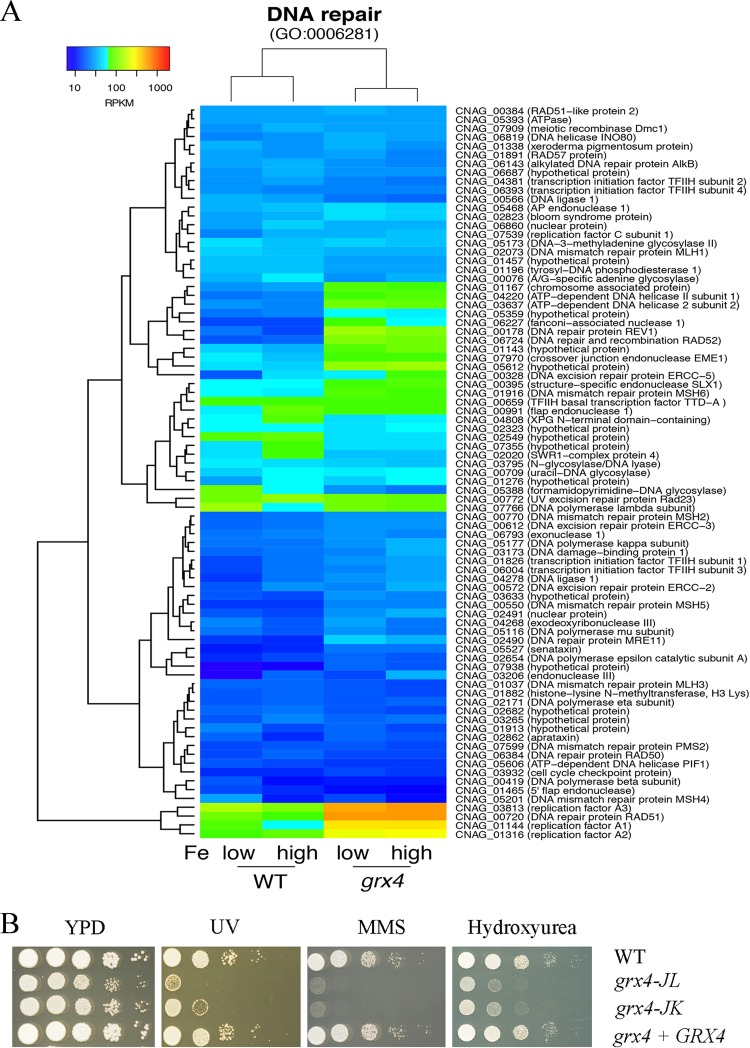
Grx4 regulates function for DNA repair and confers resistance to DNA-damaging agents. (A) Heat map representation of changes in transcript abundance of the genes encoding functions in DNA repair between the WT strain and *grx4* mutant grown under low- and high-iron conditions. (B) Spot assays on YPD medium with exposure to UV light (400 J/m^2^), the DNA repair inhibitor hydroxyurea (HU [25 mM]), and the DNA-damaging agent methyl methanesulfonate (MMS [0.03%]).

We next constructed heat maps of specific functions highlighted by the GO term analysis to further examine the influence of Grx4 on transcription. In the context of GO terms for molecular function, loss of the GRX domain of Grx4 influenced transcript levels for iron sulfur cluster binding (Fig. 8A) and heme biosynthesis (Fig. 8B). Specifically, the *grx4* mutant generally displayed elevated transcripts for these genes, a finding consistent in part with the participation of the GRX domain in the regulation of iron-using functions. We also constructed heat maps to examine the expression patterns of genes in additional categories from the GO term analysis, and these revealed further connections between Grx4 and iron-using functions for electron carrier activity (Fig. 9A). As noted above, DNA repair was the top category for the GO term analysis for biological processes. The expression patterns for genes in this category are shown in Fig. 10, and this regulation is consistent with the important role of iron as a cofactor for enzymes involved in DNA synthesis and repair (42, 43).

### Loss of the GRX domain of Grx4 results in phenotypes consistent with dysregulation of iron homeostasis.

The transcriptome changes under both low- and high-iron conditions (Fig. 7) strongly indicate that iron homeostasis is dysregulated in the *grx4* deletion mutant. We therefore examined iron-related phenotypes to confirm the biological impact of the Grx4 defect. Specifically, we tested the sensitivity of the *grx4* mutants to a wide range of inhibitors that influence iron-dependent processes, including chloroquine, which is toxic to cells with perturbed iron homeostasis, the hypoxia-mimicking agent CoCl_2_ which challenges mitochondrial function, and the inhibitor phleomycin, which is dependent on iron for toxicity (Fig. 8C). Additionally, our phenotypic assays with inhibitors of complexes of the electron transport chain (e.g., rotenone, malonic acid, antimycin A, potassium cyanide [KCN], salicylic hydroxamate [SHAM], and diphenyleneiodonium [DPI]) and agents that provoke oxidative stress (e.g., t-BOOH, H_2_O_2_, 1-chloro-2,4-dinitrobenzene [CDNB], plumbagin, menadione, and paraquat) demonstrated sensitivity for the *grx4* mutants (Fig. 9B to D). Finally, we found that the *grx4* mutants were sensitive to agents that provoke DNA damage, including UV light, methyl methanesulfonate, and hydroxyurea (Fig. 10B). The phenotypes indicated in Fig. 8 and 10 were observed both on rich medium (as shown) and on the more defined YNB medium supplemented with iron (see Fig. S6 in the supplemental material). Overall, the observed phenotypes are consistent with a role for the GRX domain of Grx4 in regulating iron homeostasis such that iron-dependent functions are dysregulated upon loss of the domain. These functions are likely to be critical for mitochondrial function and adaptation to the host environment.

10.1128/mBio.02377-18.6 FIG S6 Grx4 influences susceptibility to various inhibitors and DNA-damaging agents on minimal medium supplemented with iron. Ten-fold serial dilutions of each strain were spotted onto YNB medium plus BPS and FeCl_3_ (100**** ****μM) supplemented with the agents indicated, and the plates were incubated at 30°C for 2**** ****days before being photographed. The concentrations of the inhibitors were as follows: chloroquine, 6**** ****mM; CoCl_2_, 600**** ****μM; phleomycin, 8**** ****μg/ml; SHAM, 10**** ****mM; and paraquat, 500**** ****µM. DNA-damaging agents included UV light (100 J/m^2^) and 0.03% methyl methanesulfonate (MMS). Download FIG S6, PDF file, 0.5 MB. Copyright © 2018 Attarian et al. 2018 Attarian et al. This content is distributed under the terms of the Creative Commons Attribution 4.0 International license.

## DISCUSSION

In this study, we identified a putative monothiol glutaredoxin, Grx4, as an interaction partner with Cir1, the iron-responsive transcription factor that regulates iron uptake functions and virulence in C. neoformans. Subcellular localization studies reinforced the idea that interaction between Grx4 and Cir1 is relevant for iron sensing. That is, we found that Grx4 moves from the nucleus to the cytoplasm upon iron repletion, while Cir1 is located in the nucleus regardless of iron availability. Interestingly, the relocation of Grx4 was dependent on Cir1 because a Grx4-mCherry fusion protein remains in the cytoplasm in a *cir1* mutant. Additionally, more than one factor may contribute to retention of Grx4 in the nucleus, given that treatment with the proteasome inhibitor BTZ influenced the location of the Grx4-mCherry signal. These findings prompted a detailed characterization of the impact of a *grx4* mutation on iron homeostasis and virulence. We found that the GRX domain of Grx4 is required for robust proliferation upon iron depletion and at 37°C, as well as for the elaboration of major virulence factors, including capsule and melanin. These phenotypes were consistent with an observed virulence defect in a murine inhalation model of cryptococcosis. Subsequent transcriptional profiling revealed that Grx4 influences the expression of genes for a variety of iron-dependent functions, including DNA repair, response to oxidative stress, [2Fe-2S] cluster binding, heme binding, and oxidoreductase activity. Consistent with this regulation, a *grx4* mutant showed increased sensitivity to agents such as inhibitors of electron transport complexes that challenge functions that utilize iron.

**FIG 11:**
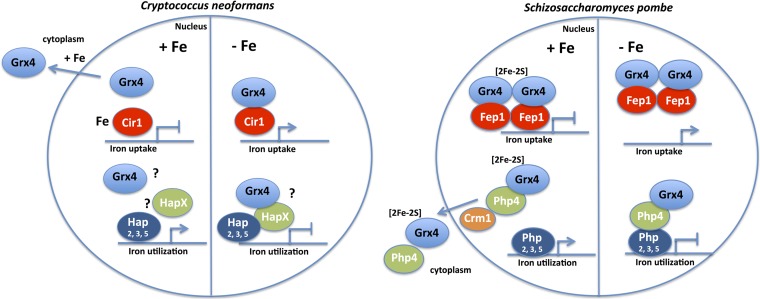
Proposed model for the interaction of Grx4 with Cir1 in C. neoformans and comparisons with Grx4 interactions in S. pombe. During iron repletion, Grx4 in C. neoformans partially relocalizes to the cytoplasm, potentially influencing the extent of its interaction with Cir1 and the repression of genes for iron uptake. Under this condition in S. pombe, Grx4 is known to interact with Fep1 but does not inhibit its ability to repress iron uptake genes (24, 26, 27, 29). Upon iron depletion, Grx4 in S. pombe inhibits Fep1, leading to depression via dissociation from the promoters of genes for iron uptake. We hypothesize that Grx4 similarly influences the activity of Cir1 upon iron limitation. Grx4 also regulates the activity of Php4 in S. pombe, and the proposed model for this yeast indicates that Grx4 promotes the exit of Php4 from the nucleus through interaction with the nuclear exportin Crm1 upon iron repletion, thereby allowing Php2, -3, and -5 to activate genes for iron utilization (32, 33). The expression of these genes is repressed by Grx4 in a complex with Php2, -3, and -5 and Php4 upon iron limitation (35). These interactions in S. pombe provide a framework for future studies of the interaction of Grx4 with HapX, the Php4 ortholog in C. neoformans.

The wealth of information from model yeasts on the mechanistic details of monothiol GRXs, [2Fe-2S] cluster binding, and transcriptional regulation provides a framework to interpret the contribution of Grx4 to iron sensing in C. neoformans (25–35, 44–46). In model yeasts, monothiol glutaredoxins play a critical role in sensing iron availability via [2Fe-2S] cluster assembly to influence the activities of transcription factors that regulate the expression of iron acquisition and iron-dependent functions (25–35). For example, the monothiol glutaredoxins Grx3 and Grx4 form [2Fe-2S] cluster binding complexes with the cytosolic proteins Fra1 and Fra2 in S. cerevisiae to dissociate the activator Aft1 from the promoters of genes for iron uptake upon iron repletion. As a result Aft1 is relocated from the nucleus to the cytoplasm. In contrast, iron deprivation results in accumulation of Aft1 in the nucleus, where it activates iron uptake, mobilization of stored iron from the vacuole, and remodeling of iron-dependent metabolism (45). As mentioned above, and summarized in the proposed model shown in Fig. 11, Grx4 regulates the iron-responsive transcription factors Fep1 and Php4 in S. pombe. Under iron-replete conditions, Grx4 binds and inactivates Php4, a repressor of genes encoding proteins for iron use. Under this condition, Php4 is retained in the cytoplasm in a Grx4-dependent manner (34). Upon iron limitation, the association of Grx4 and Php4 is reduced, and Php4 accumulates in the nucleus to repress genes encoding proteins for iron use. Deletion of Grx4 makes Php4 constitutively active and permanently located in the nucleus. Fep1 is an iron-containing protein, and bound iron is required for transcriptional repression of iron uptake functions under iron-replete conditions (24, 27, 29, 30). A Grx4-Fra2 heterodimer constitutively binds to Fep1, and iron deprivation results in disassembly of the Fe-S cluster between Grx4 and Fra2 to allow metal transfer from Fep1 to Grx4-Fra2 and derepression of iron uptake functions.

Our analysis revealed novel features of Grx4 and Cir1 compared with the studies in the model yeasts. Comparisons are most informative with S. pombe because of the detailed information on Fep1, a candidate ortholog of Cir1 in C. neoformans (Fig. 11). The interaction of Grx4 with Cir1 resembles that of Grx4 with Fep1, although Cir1 has a broader transcriptional impact beyond the regulation of iron uptake functions (15, 16). However, we lack the detailed molecular understanding of the impact of Grx4 on Cir1 activity that is available for Fep1 in S. pombe (26, 27, 29). One possible distinction for C. neoformans is the finding that the nuclear versus cytoplasmic location of the Grx4 protein is responsive to iron and heme. Grx4 *in* S. pombe appears to be located in both compartments regardless of iron availability (29) (Fig. 11). However, the Grx4 partner Php4 relocates between the nucleus and the cytoplasm in response to iron repletion in S. pombe, as mentioned above. Php4 is similar to Hap4 in S. cerevisiae and is part of the well-characterized CCAAT complex (Php2, -3, -4, and -5) (46). Orthologs in Candida albicans (Hap43) and Aspergillus fumigatus (HapX) are also involved in iron regulation and virulence (47–49). We previously characterized the function of a Php4-related protein designated HapX in C. neoformans (16). Microarray analysis revealed that HapX influences transcript levels of genes encoding iron use functions, as expected for an ortholog of Php4. HapX may therefore be an additional partner of Grx4 in C. neoformans, as hypothesized in the model shown in Fig. 11. Interestingly, HapX positively regulates the transcript levels of *CIR1* and genes encoding iron uptake functions under the low-iron condition (16). Like Php4, HapX also negatively regulates functions that use iron such as electron transport proteins. Given that Grx4 and Php4 interact in S. pombe, we predict that Grx4 and HapX proteins have similar interactions and regulatory functions in C. neoformans (Fig. 11). Experiments to examine this prediction are under way, and our preliminary yeast two-hybrid assays indicate that Grx4 interacts with HapX (E. Sánchez-León, unpublished results). In this context, it is interesting to note that Grx4 appears to make a greater contribution to the ability of C. neoformans to cause disease than HapX (16), suggesting a wider influence on functions that contribute to proliferation in vertebrate hosts. Additional proteins are known to participate in Grx4-mediated regulation of iron homeostasis in model yeasts, including BolA-like proteins (24). At least one ortholog of a BolA-like protein is predicted for C. neoformans, and studies are therefore needed to examine the role of this protein.

The interaction of Grx4 with transcription factors in model yeasts suggests that Grx4 may coregulate genes with Cir1 in C. neoformans. We found that loss of Grx4 impacted the expression of a partially overlapping set of genes, compared with a previous microarray study in which we identified the sets of genes regulated by Cir1 and HapX in response to different iron levels (15, 16). The GO terms for Cir1 regulation under low iron included iron ion transport, siderophore transport, processing of 20S pre-RNA, and rRNA metabolism (16). Additional GO term categories of DNA replication, DNA metabolism, and DNA repair were also identified in an earlier study (15). For HapX, we found that loss of this factor influenced the transcript levels for genes in the GO categories of ATP synthesis-coupled electron transport, and siderophore transport under the low-iron condition (16). As expected, the GRX domain of Grx4 contributes to the regulation of a subset of genes in the categories influenced by Cir1 and HapX. Specifically, the GO categories for transcripts impacted by Grx4 included double-strand break repair, response to oxidative stress and oxidation-reduction processes, respiratory electron transport chain, energy derivation by oxidation of organic compounds, and carbohydrate derivative metabolic process. In this regard, the pattern for Grx4 more closely resembles that of HapX, especially for functions related to electron transport. We therefore speculate, as depicted in Fig. 11, that part of the contribution of Grx4 occurs through an interaction with HapX, a protein with similarities to Php4 in S. pombe, and a shared contribution to the regulation of iron-using functions. Given that only microarray data are currently available for Cir1 and HapX, additional work is needed to obtain RNA-Seq data for mutants lacking these proteins to allow a more direct comparison of shared and distinct targets of regulation with Grx4.

We confirmed the RNA-Seq finding that Cir1 and Grx4 both participate in the regulation of a subset of genes by examining the targets *LAC1* and *FRE3* with qRT-PCR. Loss of the GRX domain of Grx4 or Cir1 resulted in elevated *LAC1* transcripts, but the impact on melanin formation was quite different. That is, a *cir1* mutant causes a hypermelanized phenotype, and our current analysis revealed a reduced melanin phenotype for the *grx4* mutant (15). These observations suggest that Grx4 might influence the expression of *LAC1* by an additional mechanism that is independent of Cir1, and in this regard it is likely that Grx4 may interact with other transcription factors (in addition to HapX). Some of these other factors may influence melanization. Conditions of the media could also have an influence because the cells for RNA-Seq analysis were grown in liquid media, and l-DOPA solid medium was used to assay melanin. Cir1 and Grx4 also positively regulated *FRE3*, as expected for shared participation in the control of a subset of iron uptake functions.

Why does loss of the GRX domain of Grx4 cause a severe virulence defect? A significant component of the contribution of Grx4 is likely due to its influence on the expression and activity of Cir1, as well as potential regulatory interactions with other transcription factors, such as HapX. In particular, the *grx4* and *cir1* mutants share defects in capsule formation, the major virulence factor, and both fail to proliferate well at 37°C. These phenotypes would certainly be expected to impair virulence. Other contributions are likely, and these include the dysregulation of iron homeostasis in the *grx4* mutant to impair adaptation to the host environment necessary to withstand defense responses (e.g., oxidative stress). In this context, human glutaredoxin is known to play an important role in redox homeostasis and protection against oxidative damage (50). Similarly, Grx3 and Grx4 in S. cerevisiae, Grx3 in C. albicans, and Grx3 in the insect pathogen Beauveria bassiana contribute to resistance to oxidative stress (51–54). A complete understanding of the contribution of Grx4 will require future work on the functional implications of interactions with Cir1, HapX, and other transcription factors and an investigation of the mechanisms of iron sensing in host tissue.

## MATERIALS AND METHODS

### Strains, plasmids, chemicals, and media.

The serotype A strain H99 (MATα) of C. neoformans var. *grubii* and mutant derivatives were maintained on YPD medium (1% yeast extract, 2% peptone, 2% dextrose, 2% agar). The nourseothricin, neomycin, and hygromycin resistance cassettes were from plasmids pCH233, pJAF1, and pJAF15 (obtained from J. Heitman), respectively. YPD medium plates containing nourseothricin (100 μg/ml) were used to select the *grx4* deletion transformants and plates containing neomycin (200 μg/ml) to select the *GRX4* reconstituted transformants in the *grx4* background. Defined low-iron medium (LIM) and yeast nitrogen base (YNB with amino acids [pH 7.0]) plus 150 μM bathophenanthroline disulfonate (BPS) (YNB-BPS) were used as iron-limiting media, as described previously (9, 11, 14). YPD and/or YNB media supplemented as indicated were used for phenotypic characterizations in growth assays on liquid and solid media. Heme was provided as hemin (Sigma-Aldrich no. H9039).

### Capsule formation and melanin production.

Capsule formation was examined by differential interference contrast (DIC) microscopy after incubation for 24 to 48 h at 30°C in defined LIM and staining with India ink. Melanin production was examined on l-3,4-dihydroxyphenylalanine (l-DOPA) plates containing 0.1% glucose.

### Serial spot dilution assays.

Overnight fungal cultures were washed twice in phosphate-buffered saline (PBS), and cell numbers were adjusted to 2 × 10^7^ cells ml^−1^. Next, 10-fold serial dilutions were prepared, and 5 μl (covering a range of 10^5^ to 10^0^ cells) was spotted onto agar medium. Plates were then incubated at 30 or 37°C for 2 days before being photographed.

### Protein-protein interaction assays.

The Grx4-Cir1 interaction assays were performed using the ProQuest two-hybrid system with Gateway Technology, Invitrogen Life Technologies, Inc., according to the manufacturer’s protocols, and as previously described (39). Briefly, the coding sequence for *GRX4* (726 bp) was synthesized by BioBasic and cloned into pDEST-32 (Gal4 DNA binding domain). The full-length *CIR1* coding region was amplified by PCR from C. neoformans cDNA using the primers listed in Table S3 in the supplemental material and cloned into pDEST-22 (Gal4 activation domain). The plasmids were then cotransformed into MaV203 yeast competent cells.

10.1128/mBio.02377-18.9 TABLE S3 Primers for strain construction and qRT-PCR. Download Table S3, DOCX file, 0.02 MB. Copyright © 2018 Attarian et al. 2018 Attarian et al. This content is distributed under the terms of the Creative Commons Attribution 4.0 International license.

The growth of MaV203 yeast expressing both bait and prey vectors was tested on synthetic complete medium (0.7% yeast nitrogen base without amino acids [Difco], 2.0% glucose, 0.07% synthetic complete selection medium mix [Sigma], 1.7% Bacto agar [Difco], pH 5.6) lacking leucine and tryptophan to select for each vector and histidine and uracil to test for an interaction. Empty pDEST-32 and pDEST-22 vectors were used as negative controls. The physical interaction between the encoded proteins in these plasmids was tested by assessing restoration of uracil and histidine prototrophy and by assaying the activity of β-galactosidase (according to the manufacturer’s protocols, and as previously described [39]).

### Deletion of the conserved GRX domain to create a *grx4* mutant and generation of a *grx4*::*GRX4* complemented strain.

A *grx4* partial deletion mutant was constructed by homologous recombination using a nourseothricin acetyltransferase (NAT) marker linked to 5′ and 3′ flanking sequences of the *GRX4* C-terminal domain by three-step overlapping PCR using primers listed in Table S3. The deletion specifically removes the coding region from amino acid 86 to the end of the polypeptide (amino acid 242). The remaining two N-terminal exons of the gene could potentially encode an 85-amino-acid polypeptide that would contain part of the TRX (thioredoxin) domain. However, transcriptome analysis of the *grx4* mutant by RNA-Seq revealed that the polypeptide was unlikely to be translated from the mRNA from the region (D. Croll, unpublished results). The overlap PCR product was biolistically transformed into WT strain H99, and deletion was confirmed by PCR and Southern blot hybridization as previously described (55–57). Genomic DNA for Southern blot analysis was prepared using cetyl trimethyl ammonium bromide (CTAB) phenol-chloroform extraction. Two independent mutants were prepared with the same deletion construct in the Lodge laboratory (*grx4-JL*) and in the Kronstad laboratory (*grx4-JK*).

To reconstitute the deleted region of *GRX4* in the *grx4* mutant, a genomic DNA fragment containing 1.1 kb of upstream promoter region and a 1.7-kb region carrying the deleted portion of the *GRX4* gene was amplified by PCR. This PCR fragment was fused with the neomycin resistance (NEO^r^) selectable marker (1.9 kb) at its C terminus in an overlap PCR. The overlap PCR product was introduced into the *grx4* mutant by biolistic transformation. Targeted integration was confirmed by PCR and Southern blot hybridization.

### Construction of a *CIR1*::*GFP* fusion allele.

The C-terminal region of the Cir1 protein was tagged with GFP (green fluorescent protein) to examine the subcellular localization of Cir1. Briefly, the upstream sequence (836 bp) and downstream sequence (826 bp) for the fusion construct were amplified from WT gDNA using the primer set Cir1-GFP-P1F and Cir1-GFP-P1R and the primer set Cir1-GFP-P5F and Cir1-GFP-P5R, respectively. The *GFP* gene and the hygromycin resistance gene (*HYG*) were amplified from the plasmid pGH022 using primers Cir1-GFP-P2F and Cir1-GFP-P3R (3,476 bp). Overlap PCR was performed using primers Cir1-GFP-P1F and Cir1-GFP-P5R to yield the 5,138-bp construct. The construct was then used to transform the WT and *grx4* mutant strains by biolistic transformation. Following transformation, mutants were screened for resistance to hygromycin, and the proper location and orientation of *GFP* were determined by PCR. Primer sequences are listed in Table S3.

### Construction of a *GRX4*::*mCherry* fusion allele.

The C-terminal region of the Grx4 protein was tagged with mCherry to examine the subcellular localization of Grx4. Briefly, the upstream sequence (548 bp) and downstream sequence (508 bp) for the fusion construct were amplified from WT gDNA using the primer set Grx4-mCherry-P1F and Grx4-mCherry-P1R and the primer set Grx4-mCherry-P3F and Grx4-mCherry-P3R, respectively. The gene encoding mCherry and the hygromycin resistance gene (*HYG*) were amplified from the plasmid pGH026 using primers Grx4-mCherry-P2F and Grx4-mCherry-P2R (3,524 bp). Overlap PCR was performed using primers Cir1-GFP-P1F and Cir1-GFP-P3R to yield the 4,580-bp construct. The construct was then used to transform the WT (H99), WT::Cir1-GFP, and *cir1* mutant strains by biolistic transformation. Following biolistic transformation, mutants were screened for resistance to hygromycin, and the proper location and orientation of mCherry were determined by PCR. Primer sequences are listed in Table S3.

### Quantitation of Grx4-mCherry fluorescence in the nucleus versus the cytoplasm.

Laser scanning confocal fluorescence images were analyzed to determine signal intensities of mCherry fluorescence in the cytoplasm and nuclei in response to different iron sources/levels for the C. neoformans strain coexpressing Grx4-mCherry and Cir1-GFP. The mean gray values of selected fluorescent areas (integrated density) showing Grx4-mCherry corresponding to the nucleus and the cytoplasm (nonnuclear) were obtained for each cell. Integrated densities of Grx4-mCherry signal in the cytoplasm were obtained by subtracting the nuclear region of the cells and measuring the remaining fluorescence. All measurements were corrected for each image background. One-way analysis of variance (ANOVA) and Tukey statistical tests were performed to analyze the signal intensity data of the C. neoformans strain under low-iron (LIM) and iron-supplemented (LIM plus 10 μM FeCl_3_ or heme) treatments. Both tests revealed statistically significant differences between the treatments (*P* < 0.0001). Laser scanning confocal fluorescence microscopy was performed with an inverted Olympus Fluoview FV1000 confocal microscope ﬁtted with an argon laser (GFP: excitation, 488 nm; emission, 510 nm) and a He/Ne laser (mCherry: excitation, 543 nm; emission, 612 nm), along with a 100× UplanS Apo (Olympus) oil-immersion objective (NA, 1.40). Confocal images were captured and examined using FV10-ASW software (version 4.2.3.6; Olympus). Fluorescence signal intensity measurements and statistical analyses were performed with ImageJ (version 1.51w; NIH, Bethesda, MD) and Prism 6 (version 6.01; GraphPad Software).

### RNA-Seq analysis of gene expression.

Cells for three biological replicates were prepared by growing the WT strain and the *grx4* mutant in 50 ml of YPD overnight at 30˚C. The cells were washed twice with iron-chelated water followed by growth in 50 ml of YNB low-iron medium for 16 h, harvested, and diluted to 4.0 × 10^7^ cells in 50 ml of the same medium with or without 100 μM FeCl_3_. Cultures were incubated at 30˚C for 6 h and harvested for RNA extraction. Total RNA was extracted by RiboPure-Yeast kit (Life Technologies, Carlsbad, CA) and treated with DNase I (Life Technologies, Carlsbad, CA) following the manufacturer’s instructions. The quantity and integrity of the total RNA were evaluated using a 2100 Bioanalyzer (Agilent Technologies, Palo Alto, CA). The samples for verification of RNA-Seq data by quantitative PCR (qPCR) were prepared the same as described above. The primers used for qPCR are listed in Table S3.

### Read alignment and quantification.

Raw Illumina reads were quality trimmed and filtered for adapter contamination using Trimmomatic v.0.36 (58). The following settings were applied: "ILLUMINACLIP:TruSeq3-PE.fa:2:30:10 LEADING:10 TRAILING:10 SLIDINGWINDOW:5:10 MINLEN:50." Reads passing the filters were aligned to the latest C. neoformans var. *grubii* H99 reference genome (38) using hisat v.2.1.0 with parameters –min-intronlen 20 –max-intronlen 1000 (59). The genome sequence and annotation were downloaded from ENSEMBL (http://fungi.ensembl.org) in August 2017. The produced SAM files were position sorted using samtools v.1.5 (60). Reads overlapping annotated transcripts of H99 were quantified with HTSeq-count v.0.9.1, requiring a minimum alignment quality of 10 and setting the matching mode to “union” (60, 61).

Read counts were normalized across samples and replicates using the Biocondutor package edgeR v.3.6 (62). We removed genes with total counts below 3 prior to normalization (63). Library sizes were normalized using trimmed mean of M values (TMM), and gene-level counts were normalized across conditions using RPKM (reads per kilobase of transcript per million mapped reads). For this, the edgeR functions *cpm* and *rpkm* were used. Differential expression was tested using the edgeR function *exactTest*, which tests for mean expression differences based on negative binomially distributed counts. Significance values were corrected for multiple testing using the Benjamin-Hochberg false-discovery rate (FDR). We restricted our analyses to genes with an expression difference of at least 2-fold and an FDR *P* value of <0.001. Log_10_-scaled gene expression values were visualized using the *heatmap.2* function in the R package gplots v.3.0.1.

### Analyses of enrichment in protein functions.

For each comparison among conditions (iron levels and *grx4* genotypes), sets of differentially expressed genes were analyzed for an enrichment in encoded protein functions. Predicted proteins were assigned to Gene Ontology (GO) terms using InterProScan 5.26-65 (64). GO terms were only considered if the total term size in the genome was at least 5. For each comparison, hypergeomtric tests were performed to test for enrichment, and GO terms with an FDR *P* value of <0.001 were considered significant. All enrichment analyses were performed using the R packages GSEABase and GOstats (65). Outcomes of enrichment tests were visualized using the R package ggplot2 (66).

### Virulence assays.

For virulence assays, female BALB/c mice (4 to 6 weeks old) were obtained from Charles River Laboratories (Ontario, Canada). The WT, *grx4* mutant, and *grx4*::*GRX* cells were grown in YPD overnight at 30°C, washed in PBS, and resuspended at 1.0 × 10^6^ cells ml^−1^ in PBS. Inoculation was by intranasal instillation with 50 μl of cell suspension (inoculum of 2.0 × 10^5^ cells per mouse). Groups of 10 mice were inoculated for each strain. The status of the mice was monitored twice daily postinoculation. For the determination of fungal burdens in organs, infected mice were euthanized by CO_2_ inhalation, and organs were excised, weighed, and homogenized in 1 ml of PBS using a MixerMill (Retsch). Serial dilutions of the homogenates were plated on YPD agar plates containing 35 μg/ml chloramphenicol, and CFU were counted after incubation for 48 h at 30°C.

Furthermore, the fungal load distribution in different tissues of the infected mice was determined. Mice reaching the endpoint were euthanized by CO_2_ asphyxiation, and fungal loads in different tissues of the mice, including the brains, lungs, and spleen were determined. Tissues were aseptically removed and immersed in PBS. Organs were homogenized using an automated tissue homogenizer. The samples were serially diluted in PBS and plated on YPD supplemented with 35 μg/ml chloramphenicol. After 2 days of incubation at 30°C, the colony-forming units (CFU) were counted manually. All experiments with mice were conducted in accordance with the guidelines of the Canadian Council on Animal Care and approved by the University of British Columbia's Committee on Animal Care (protocol A17-0117).

### Availability of data.

All raw sequencing reads are available from the NCBI Short Read Archive (SRA) under accession no. SRR7446976 to SRR7446987 and under BioProject no. PRJNA478320 (see Table S4 in the supplemental material).

10.1128/mBio.02377-18.10 TABLE S4 BioProject and BioSample accession numbers for the RNA-Seq data. Download Table S4, DOCX file, 0.07 MB. Copyright © 2018 Attarian et al. 2018 Attarian et al. This content is distributed under the terms of the Creative Commons Attribution 4.0 International license.
