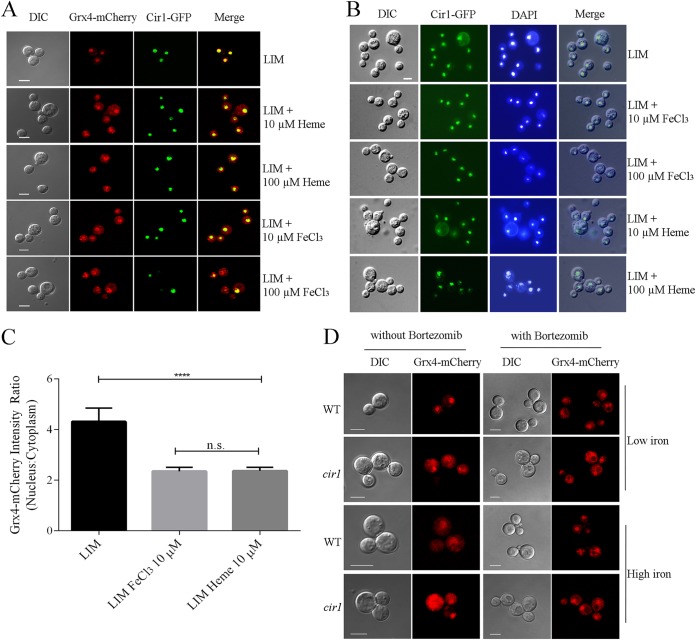# Erratum for Attarian et al., “The Monothiol Glutaredoxin Grx4 Regulates Iron Homeostasis and Virulence in Cryptococcus neoformans”

**DOI:** 10.1128/mBio.00647-19

**Published:** 2019-04-30

**Authors:** Rodgoun Attarian, Guanggan Hu, Eddy Sánchez-León, Mélissa Caza, Daniel Croll, Eunsoo Do, Horacio Bach, Tricia Missall, Jennifer Lodge, Won Hee Jung, James W. Kronstad

**Affiliations:** a Michael Smith Laboratories, University of British Columbia, Vancouver, British Columbia, Canada; b Department of Microbiology and Immunology, University of British Columbia, Vancouver, British Columbia, Canada; c Laboratory of Evolutionary Genetics, Institute of Biology, University of Neuchâtel, Neuchâtel, Switzerland; d Department of Systems Biotechnology, Chung-Ang University, Anseong, South Korea; e Department of Biochemistry, Saint Louis University School of Medicine, St. Louis, Missouri, USA; f Department of Molecular Microbiology, Washington University School of Medicine, St. Louis, Missouri, USA

## ERRATUM

Volume 9, no. 6, e02377-18, 2018, https://doi.org/10.1128/mBio.02377-18. In Fig. 2B, we made a mistake in the assembly of the panels such that the images for the cells grown in LIM were used in both the panel labeled LIM and the panel labeled LIM + 100 μM heme. A new version of Fig. 2 has been prepared to show the correct images for the cells from the LIM + 100 μM heme condition. This mistake in figure preparation does not change any of the interpretations or conclusions in our paper. The Cir1-GFP fusion protein is located in the nucleus under all conditions. We apologize for any inconvenience.

**FIG 2:**