# Supplementary material for: The Monothiol Glutaredoxin Grx4 Regulates Iron Homeostasis and Virulence in Cryptococcus neoformans
Source: mBio. 2018 Dec 4;9(6):e02377-18. doi: 10.1128/mBio.02377-18 (PMC6282196; doi:10.1128/mBio.02377-18)

Figure S1

A

```
UhGrx4        1 CKKLMEQSKVMLFMKGDPDTPKCGFSQKTVNLFRQEKVE---FGHYDILKDENVRQGLKK
UmGrx4        1 CKKLMEQSKVMLFMKGDPDTPRCGFSQKTVNLLRQEKVD---FGHYDILKDENVRQGLKK
SpGrx4        1 ---LTNAHNVMLFLKGTPSEPACGFSRKLVGLLREQNVQ---YGFFNILADDSVRQGLKV
ScGrx3        1 --KLVNAAPVMLFMKGSPSEPKCGFSRQLVGILREHQVR---FGFFDILRDESVRQNLKK
ScGrx4        1 --KLVQAAPVMLFMKGSPSEPKCGFSRQLVGILREHQIR---FGFFDILRDENVRQSLKK
CaGrx4        1 -NKLTKAAPIMLFMKGSPSSPQCGFSRQLVAILREHQVR---FGFFDILKDDSVRQGLKK
HsGrx3        1 ---LTNKASVMLFMKGNKQEAKCGFSKQILEILNSTGVE---YETFDILEDEEVRQGLKA
ScGrx5        1 -EDAIESAPVVLFMKGTPEFPKCGFSRATIGLLGNQGVDPAKFAAYNVLEDPELREGIKE
HsGrx5        1 ---LVKKDKVVVFLKGTPEQPQCGFSNAVVQILRLHGVR--DYAAYNVLDDPELRQGIKD
CnGrx4        1 CHELMNKHKVVLFMKGNPTAPKCGFSRQTVGLLREQGVE---FAWFDIFSDEDVRQGLKK
HcGrx4        1 LDELVKAAPVMLFMKGTPSAPQCGFSRQLVGILRENGVK---YGFFNILADEDVRQGLKE
consensus     1    ..  . ...*.**... ..****.........  .    ....... *. .*...*.

UhGrx4       58 LNEWPTFPQIIVNGELIGGLDILKESIESGEFQ
UmGrx4       58 LNEWPTFPQIIVNGELIGGLDILKESIESGEFQ
SpGrx4       55 FSDWPTFPQLYIKGEFVGGLDIVSEMIENGELQ
ScGrx3       56 FSEWPTFPQLYINGEFQGGLDIIKESLE-----
ScGrx4       56 FSDWPTFPQLYINGEFQGGLDIIKESIE-----
CaGrx4       57 FSDWPTFPQLYINGEFQGGLDIIKESIEDDE--
HsGrx3       55 YSNWPTYPQLYVKGELVGGLDIVKELKENGE--
ScGrx5       60 FSEWPTIPQLYVNKEFIGGCDVITSMARSGE--
HsGrx5       56 YSNWPTIPQVYLNGEFVGGCDILLQMHQNGD--
CnGrx4       58 VNDWPTFPQIIVNGELVGGLDILREMIENGEWQ
HcGrx4       58 YADWPTFPQLWVKGELVGGLDIVKEEIATN---
consensus    61 ...***.**.....*..**.*.... .. ..
```

B

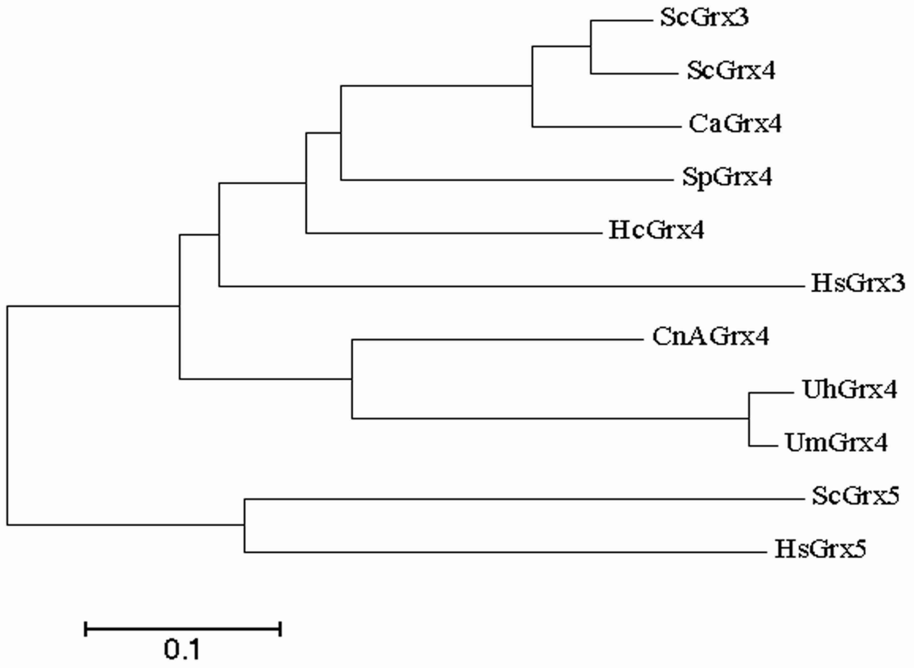

Supplement: FIG S1 [file mbo006184204sf1.pdf]
