# Supplementary figures and images for: The Monothiol Glutaredoxin Grx4 Regulates Iron Homeostasis and Virulence in Cryptococcus neoformans
Source: mBio. 2018 Dec 4;9(6):e02377-18. doi: 10.1128/mBio.02377-18 (PMC6282196; doi:10.1128/mBio.02377-18)

Figure S2

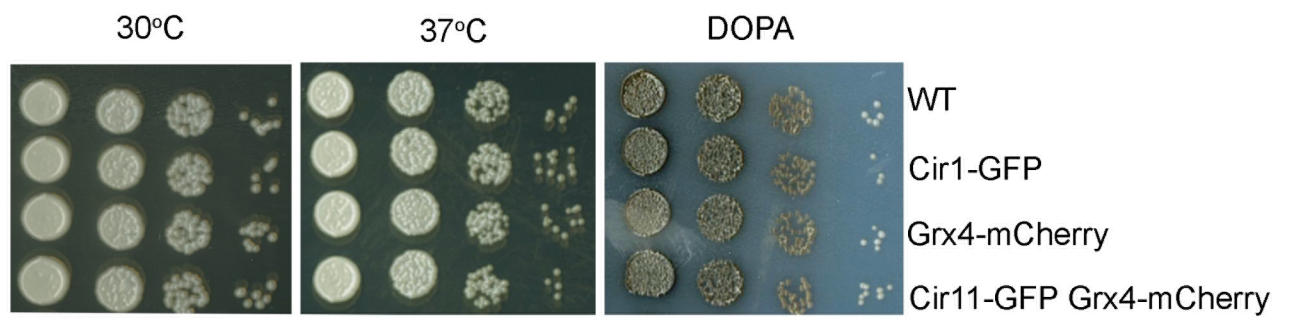

Supplement: FIG S2 [file mbo006184204sf2.pdf]

Figure S3

A

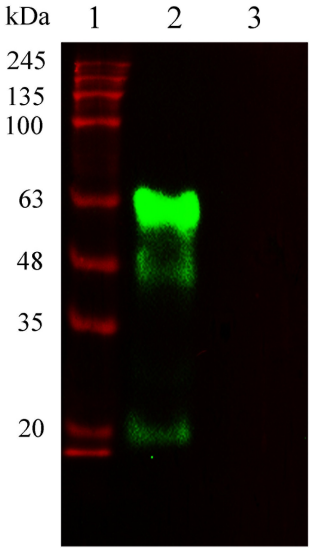

1: protein ladder  
2: Grx4-mCherry  
3: WT (H99)

B

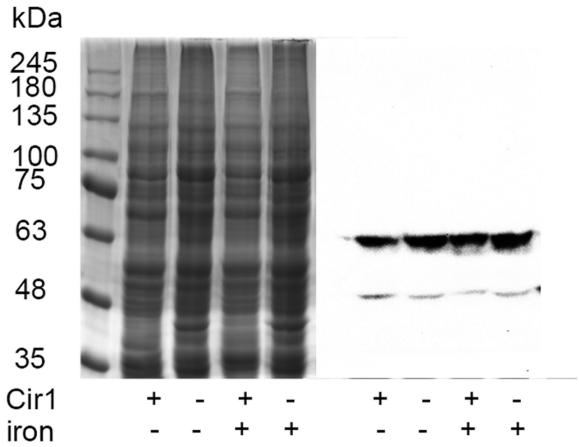

C

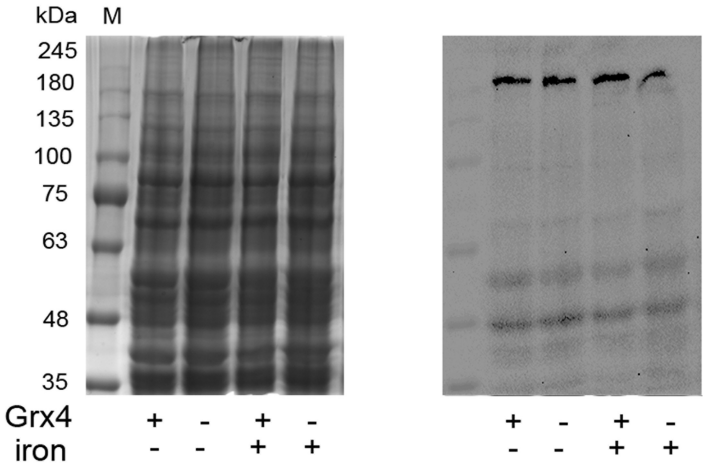

Supplement: FIG S3 [file mbo006184204sf3.pdf]

Figure S4

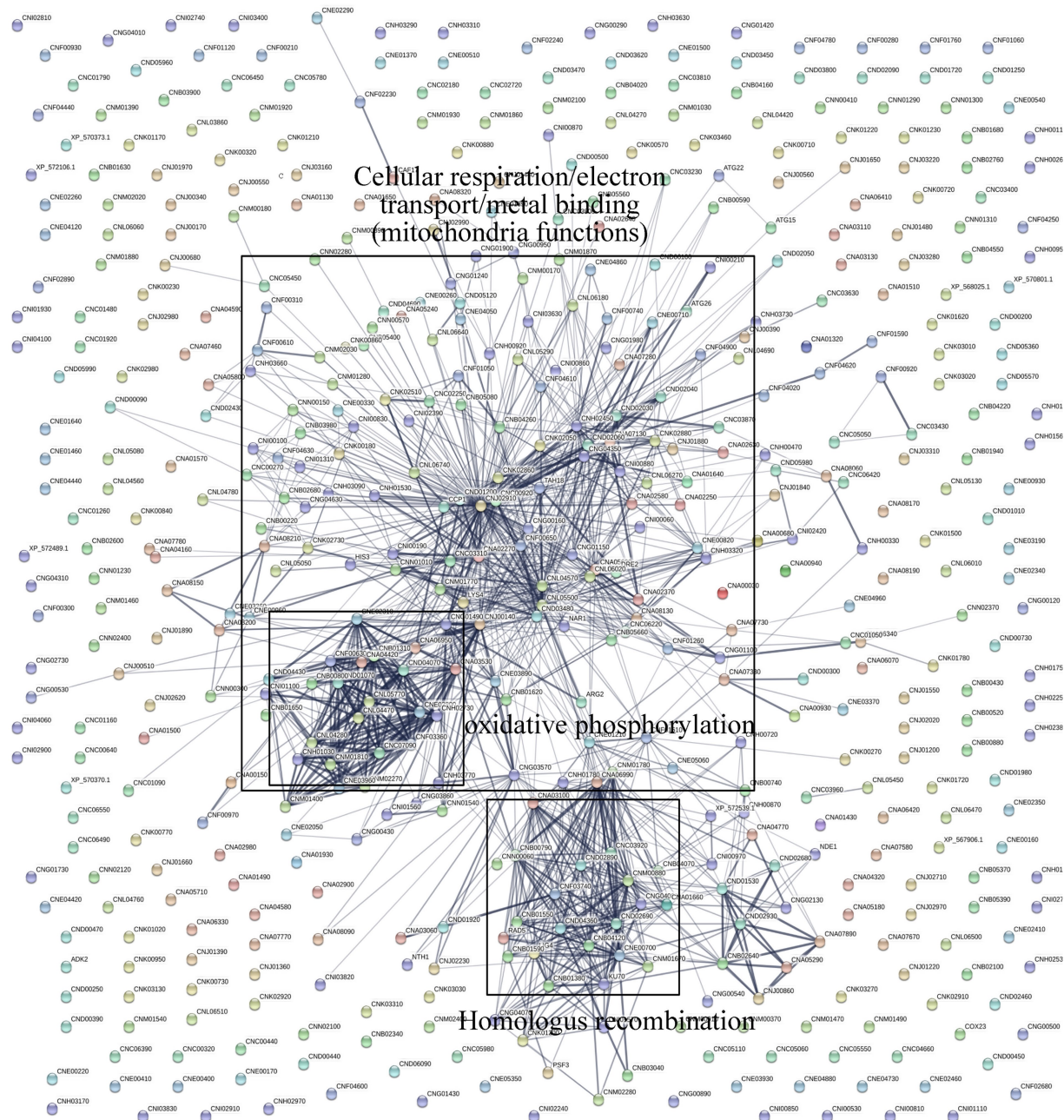

Supplement: FIG S4 [file mbo006184204sf4.pdf]

Figure S5

A

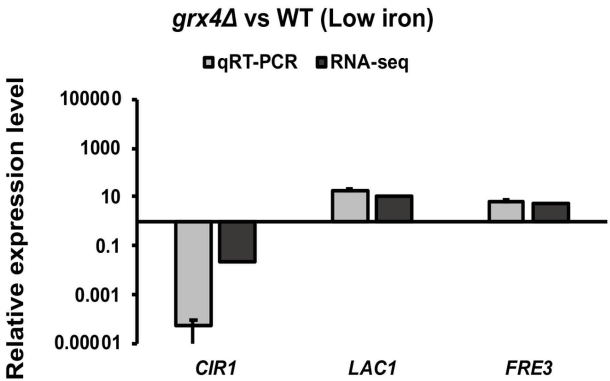

B

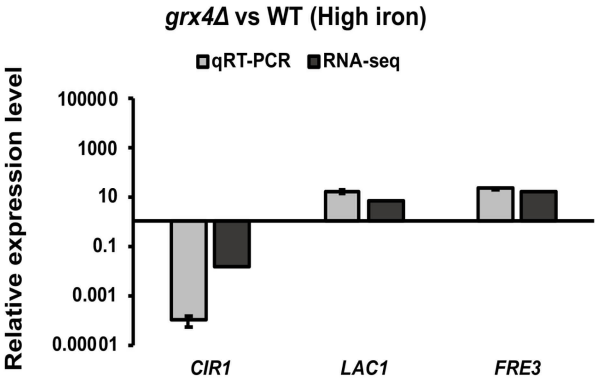

Supplement: FIG S5 [file mbo006184204sf5.pdf]

Figure S6

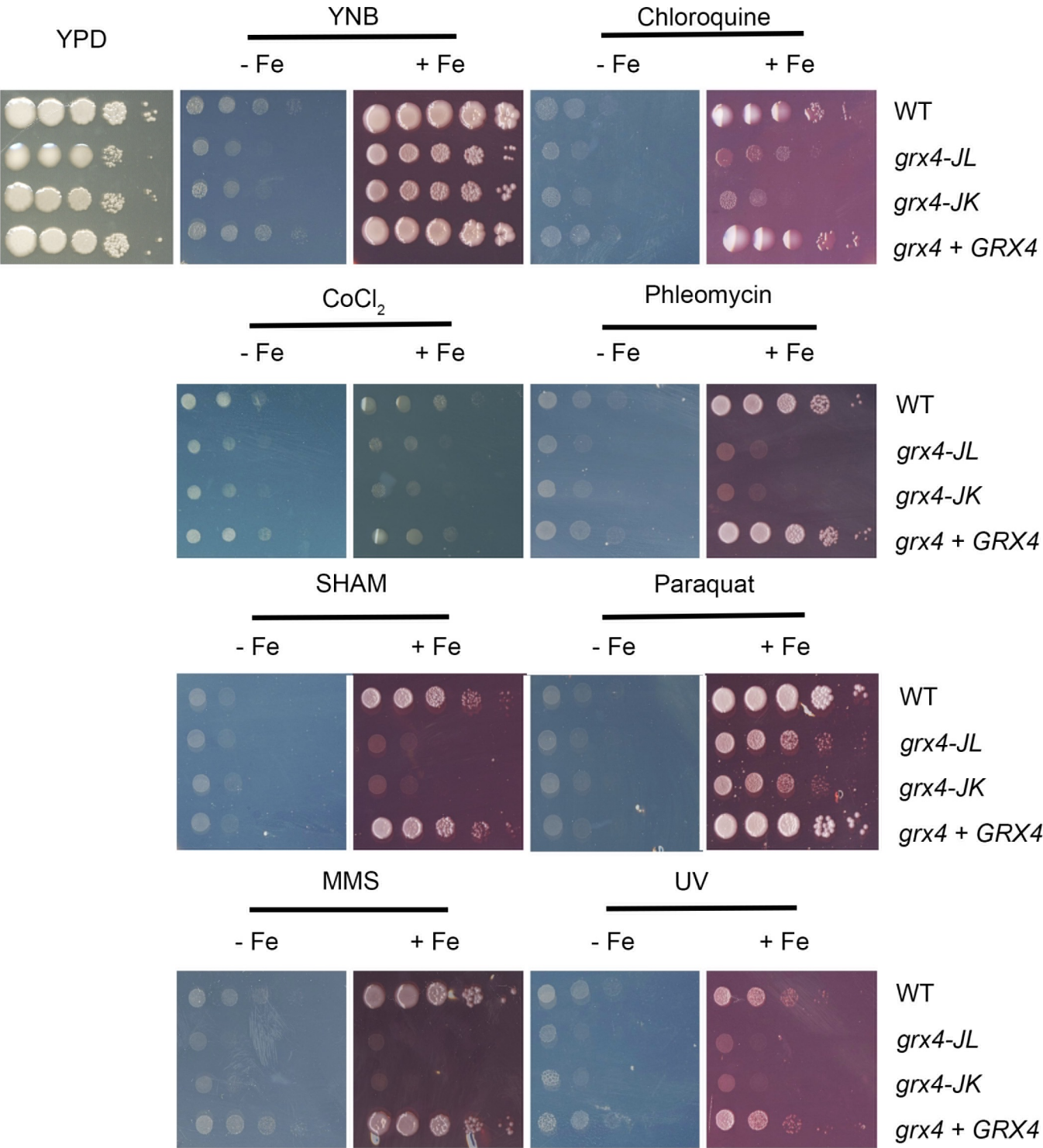

Supplement: FIG S6 [file mbo006184204sf6.pdf]
