# Supplementary material for: The Monothiol Glutaredoxin Grx4 Regulates Iron Homeostasis and Virulence in Cryptococcus neoformans
Source: mBio. 2018 Dec 4;9(6):e02377-18. doi: 10.1128/mBio.02377-18 (PMC6282196; doi:10.1128/mBio.02377-18)
Supplement: TABLE S1 [file mbo006184204st1.docx]

Table S1. Transcripts up-regulated (639) in *grx4* under both low iron and high iron conditions

| **Name** | **Description** | **WT-L vs WT-H** | **grx4-L vs WT-L** | **grx4-H vs WT-H** | **grx4-L vs grx4-H** |
| --- | --- | --- | --- | --- | --- |
| CNAG_01138 | cytochrome c mitochondrial | 0.07 | 181.00 | 27.35 | 0.44 |
| CNAG_07367 | amino acid transporter | 1.38 | 171.48 | 184.74 | 1.27 |
| CNAG_07734 | hypothetical protein CNAG_07734 | #N/A | 162.38 | #N/A | 1.44 |
| CNAG_06312 | hypothetical protein CNAG_06312 | 1.48 | 92.13 | 24.11 | 5.62 |
| CNAG_04890 | hypothetical protein CNAG_04890 | #N/A | 60.46 | 56.28 | 0.87 |
| CNAG_02049 | proline dehydrogenase | 3.87 | 60.35 | 181.08 | 1.28 |
| CNAG_04325 | extradiol ring-cleavage dioxygenase | 0.11 | 58.01 | 16.73 | 0.38 |
| CNAG_00162 | alternative mitochondrial | 0.02 | 52.56 | 1.80 | 0.68 |
| CNAG_01056 | conidiation-specific 6 | 1.52 | 48.48 | 62.44 | 1.17 |
| CNAG_02548 | cobalamin synthesis | 0.04 | 32.91 | 3.45 | 0.35 |
| CNAG_01995 | hypothetical protein CNAG_01995 | 1.67 | 31.85 | 61.04 | 0.86 |
| CNAG_07797 | transcriptional regulator | 0.23 | 29.78 | 7.52 | 0.90 |
| CNAG_07969 | hypothetical protein CNAG_07969 | 2.70 | 26.56 | 48.76 | 1.45 |
| CNAG_02526 | hypothetical protein CNAG_02526 | 4.08 | 26.35 | 72.67 | 1.47 |
| CNAG_04469 | 4-aminobutyrate transaminase | 1.10 | 26.26 | 38.63 | 0.74 |
| CNAG_01965 | hypothetical protein CNAG_01965 | 1.06 | 24.64 | 45.84 | 0.57 |
| CNAG_06557 | membrane protein | 0.61 | 23.19 | 24.33 | 0.57 |
| CNAG_06911 | hypothetical protein CNAG_06911 | 1.40 | 22.15 | 26.62 | 1.16 |
| CNAG_04138 | hypothetical protein CNAG_04138 | #N/A | 21.42 | 69.59 | 0.73 |
| CNAG_00735 | aldehyde dehydrogenase family 7 member A1 | 0.93 | 20.90 | 12.26 | 1.57 |
| CNAG_01075 | methylmalonate-semialdehyde dehydrogenase (acylating) | 0.97 | 18.92 | 20.53 | 0.89 |
| CNAG_07310 | hypothetical protein CNAG_07310 | 0.02 | 18.18 | 1.83 | 0.20 |
| CNAG_04951 | 3-deoxy-7-phosphoheptulonate synthase | 0.09 | 17.72 | 2.51 | 0.63 |
| CNAG_00540 | pantothenate transporter | 0.90 | 17.35 | 24.46 | 0.63 |
| CNAG_01081 | hypothetical protein CNAG_01081 | 1.68 | 17.11 | 24.94 | 1.14 |
| CNAG_07798 | amidohydrolase 3 | 1.27 | 16.91 | 26.40 | 0.81 |
| CNAG_02777 | phosphate:H symporter | 0.25 | 16.59 | 5.88 | 0.71 |
| CNAG_00844 | MFS transporter | 2.55 | 15.74 | 41.31 | 0.96 |
| CNAG_04617 | OPT family small oligopeptide transporter | 0.39 | 15.46 | 12.63 | 0.47 |
| CNAG_06651 | amidohydrolase domain containing | 3.81 | 15.02 | 48.32 | 1.18 |
| CNAG_06194 | hypothetical protein CNAG_06194 | 0.84 | 14.90 | 11.69 | 1.07 |
| CNAG_06556 | oxidoreductase | 2.21 | 14.66 | 27.83 | 1.15 |
| CNAG_03011 | glycerate-and formate-dehydrogenase | 1.01 | 14.52 | 13.40 | 1.08 |
| CNAG_05341 | hypothetical protein CNAG_05341 | 1.37 | 13.91 | 18.90 | 1.00 |
| CNAG_02489 | alcohol propanol-preferring | 3.62 | 13.23 | 29.05 | 1.63 |
| CNAG_05324 | sugar transporter | 1.17 | 13.11 | 32.80 | 0.47 |
| CNAG_07550 | hypothetical protein CNAG_07550 | 0.39 | 13.08 | 4.55 | 1.13 |
| CNAG_06294 | hypothetical protein CNAG_06294 | 0.60 | 12.76 | 4.00 | 1.88 |
| CNAG_06145 | RNA processing-related | 0.99 | 12.66 | 11.83 | 1.05 |
| CNAG_07811 | hypothetical protein CNAG_07811 | #N/A | 12.31 | #N/A | 1.56 |
| CNAG_01971 | hypothetical protein CNAG_01971 | #N/A | 12.24 | 13.33 | 2.36 |
| CNAG_05847 | thioredoxin reductase | 1.24 | 12.12 | 10.13 | 1.47 |
| CNAG_07827 | hypothetical protein CNAG_07827 | 0.39 | 11.87 | 4.53 | 1.02 |
| CNAG_01542 | taurine catabolism dioxygenase | 3.06 | 11.82 | 26.91 | 1.33 |
| CNAG_05763 | hypothetical protein CNAG_05763 | 0.07 | 11.36 | 1.99 | 0.37 |
| CNAG_03906 | hypothetical protein CNAG_03906 | 1.90 | 10.87 | 20.52 | 1.00 |
| CNAG_01908 | uroporphyrinogen-III synthase | 0.04 | 10.60 | 2.72 | 0.14 |
| CNAG_04242 | hypothetical protein CNAG_04242 | 0.09 | 10.36 | 2.87 | 0.31 |
| CNAG_03465 | laccase precursor | 0.99 | 10.22 | 7.55 | 1.32 |
| CNAG_04357 | hypothetical protein CNAG_04357 | 2.58 | 10.16 | 21.14 | 1.23 |
| CNAG_00838 | hypothetical protein CNAG_00838 | 1.23 | 9.84 | 9.00 | 1.33 |
| CNAG_00177 | hypothetical protein CNAG_00177 | 2.93 | 9.73 | 16.82 | 1.68 |
| CNAG_05015 | CAT1 catalase | 0.65 | 9.63 | 5.65 | 1.09 |
| CNAG_00237 | 3-isopropylmalate large subunit | 0.22 | 9.54 | 3.01 | 0.70 |
| CNAG_04988 | Gly-Xaa carboxypeptidase | 0.81 | 9.38 | 10.70 | 0.70 |
| CNAG_04470 | haloacid type II | 1.51 | 9.29 | 14.57 | 0.95 |
| CNAG_07693 | high-affinity methionine permease | 1.62 | 9.20 | 9.04 | 1.63 |
| CNAG_00161 | auxin-induced protein | 0.63 | 9.11 | 5.78 | 0.98 |
| CNAG_02525 | hypothetical protein CNAG_02525 | 1.56 | 9.05 | 7.49 | 1.87 |
| CNAG_02565 | homoaconitate hydratase | 0.08 | 8.81 | 1.64 | 0.40 |
| CNAG_01947 | 2,4-dienoyl- reductase | 0.97 | 8.79 | 7.14 | 1.19 |
| CNAG_04103 | hypothetical protein CNAG_04103 | 0.50 | 8.77 | 4.62 | 0.95 |
| CNAG_04905 | tRNA (uracil-5-)-methyltransferase | 0.03 | 8.67 | 2.14 | 0.14 |
| CNAG_06650 | hypothetical protein CNAG_06650 | 1.25 | 8.62 | 15.95 | 0.67 |
| CNAG_02093 | hypothetical protein CNAG_02093 | 0.33 | 8.54 | 3.26 | 0.86 |
| CNAG_00462 | electron-transferring-flavo dehydrogenase | 0.05 | 8.51 | 1.35 | 0.29 |
| CNAG_06374 | malate dehydrogenase (oxaloacetate-decarboxylating) | 0.95 | 8.28 | 2.99 | 2.59 |
| CNAG_02734 | hypothetical protein CNAG_02734 | #N/A | 8.21 | 14.40 | 1.20 |
| CNAG_04461 | ATP-dependent DNA helicase HFM1 MER3 | 1.38 | 8.11 | 8.97 | 1.24 |
| CNAG_07711 | PLP-dependent transferase | 2.27 | 7.94 | 7.95 | 2.23 |
| CNAG_02771 | DNA repair and recombination RAD54B | 1.10 | 7.91 | 8.79 | 0.98 |
| CNAG_07779 | D-glycerate 3-kinase | 0.57 | 7.83 | 4.17 | 1.05 |
| CNAG_02900 | hypothetical protein CNAG_02900 | 1.88 | 7.78 | 8.73 | 1.66 |
| CNAG_01714 | sulfonate dioxygenase | 1.62 | 7.58 | 14.24 | 0.85 |
| CNAG_07960 | hypothetical protein CNAG_07960 | 1.20 | 7.42 | 9.16 | 0.97 |
| CNAG_04862 | glutamate synthase (NADPH NADH) | 0.22 | 7.36 | 7.15 | 0.22 |
| CNAG_05093 | hypothetical protein CNAG_05093 | #N/A | 7.32 | 6.17 | 1.24 |
| CNAG_00720 | DNA repair RAD51 | 1.51 | 7.11 | 10.72 | 0.99 |
| CNAG_01400 | 3-deoxy-7-phosphoheptulonate synthase | 0.11 | 6.86 | 1.70 | 0.45 |
| CNAG_07552 | DNA repair Rad8 | 0.87 | 6.75 | 4.97 | 1.17 |
| CNAG_01969 | zinc metalloprotease | 1.19 | 6.71 | 3.50 | 2.27 |
| CNAG_00840 | hypothetical protein CNAG_00840 | 1.09 | 6.71 | 7.08 | 1.02 |
| CNAG_01061 | serine threonine kinase | 0.85 | 6.70 | 3.55 | 1.59 |
| CNAG_04691 | hypothetical protein CNAG_04691 | 1.64 | 6.69 | 9.87 | 1.10 |
| CNAG_01949 | chlorophyll synthesis pathway | 1.10 | 6.63 | 4.30 | 1.68 |
| CNAG_00937 | hypothetical protein CNAG_00937 | 2.27 | 6.58 | 12.16 | 1.22 |
| CNAG_00133 | hypothetical protein CNAG_00133 | 1.57 | 6.52 | 8.59 | 1.18 |
| CNAG_06203 | hypothetical protein CNAG_06203 | 1.80 | 6.49 | 11.88 | 0.98 |
| CNAG_04267 | mitochondrial genome maintenance | 0.18 | 6.48 | 1.82 | 0.62 |
| CNAG_05357 | hypothetical protein CNAG_05357 | 0.44 | 6.32 | 10.04 | 0.28 |
| CNAG_03635 | hypothetical protein CNAG_03635 | 2.31 | 6.28 | 8.25 | 1.75 |
| CNAG_06724 | DNA strand annealing | 0.98 | 6.23 | 5.12 | 1.18 |
| CNAG_04025 | transaldolase | 3.30 | 6.23 | 11.60 | 1.76 |
| CNAG_00868 | hypothetical protein CNAG_00868 | #N/A | 6.18 | 28.26 | 0.62 |
| CNAG_01139 | hypothetical protein CNAG_01139 | 1.14 | 6.17 | 8.59 | 0.81 |
| CNAG_02690 | pirin | 0.55 | 6.17 | 3.63 | 0.92 |
| CNAG_00178 | DNA repair REV1 | 1.40 | 6.12 | 7.12 | 1.19 |
| CNAG_07796 | uroporphyrinogen-III C-methyltransferase | 0.16 | 6.11 | 4.07 | 0.23 |
| CNAG_00235 | ammonium transporter MEP1 | 0.39 | 6.10 | 5.39 | 0.44 |
| CNAG_04416 | major facilitator superfamily transporter | 1.96 | 6.02 | 10.87 | 1.07 |
| CNAG_04704 | MFS SHS lactate transporter | 1.14 | 6.02 | 8.87 | 0.77 |
| CNAG_04758 | amt family ammonium transporter | 1.01 | 5.94 | 7.27 | 0.82 |
| CNAG_00549 | hypothetical protein CNAG_00549 | 1.68 | 5.91 | 7.35 | 1.33 |
| CNAG_06166 | ATP-dependent DNA helicase MPH1 | 1.00 | 5.89 | 5.13 | 1.14 |
| CNAG_05497 | dihydroxy-acid dehydratase | 0.10 | 5.86 | 1.24 | 0.48 |
| CNAG_01865 | hypothetical protein CNAG_01865 | 1.10 | 5.84 | 3.51 | 1.81 |
| CNAG_05509 | imidazoleglycerol-phosphate dehydratase | 0.15 | 5.77 | 1.58 | 0.55 |
| CNAG_06259 | alpha-glucoside:hydrogen symporter | 2.21 | 5.74 | 7.70 | 1.63 |
| CNAG_02147 | cytochrome c peroxidase | 0.07 | 5.71 | 1.94 | 0.20 |
| CNAG_07309 | mRNA surveillance pelota | 0.06 | 5.69 | 1.24 | 0.28 |
| CNAG_04055 | cell cycle checkpoint | 1.16 | 5.66 | 6.47 | 1.01 |
| CNAG_07908 | aconitate mitochondrial | 0.20 | 5.64 | 2.13 | 0.53 |
| CNAG_06152 | hypothetical protein CNAG_06152 | 1.10 | 5.62 | 5.94 | 1.03 |
| CNAG_02312 | patatin-like phospholipase domain-containing | 0.53 | 5.62 | 2.95 | 1.01 |
| CNAG_01668 | hypothetical protein CNAG_01668 | 1.37 | 5.61 | 8.81 | 0.86 |
| CNAG_00275 | hypothetical protein CNAG_00275 | 2.24 | 5.61 | 7.10 | 1.75 |
| CNAG_06910 | Metallo-hydrolase oxidoreductase | 1.08 | 5.60 | 9.95 | 0.60 |
| CNAG_05875 | cytochrome c heme-lyase | 0.17 | 5.60 | 2.99 | 0.31 |
| CNAG_00575 | catalase A | 0.48 | 5.59 | 3.31 | 0.81 |
| CNAG_05147 | hypothetical protein CNAG_05147 | 2.22 | 5.50 | 5.00 | 2.42 |
| CNAG_05198 | DNA repair RAD7 | 1.27 | 5.49 | 7.27 | 0.95 |
| CNAG_07766 | DNA polymerase lambda subunit | 1.87 | 5.49 | 9.21 | 1.10 |
| CNAG_03696 | hypothetical protein CNAG_03696 | 2.04 | 5.49 | 7.09 | 1.56 |
| CNAG_05434 | NADH dehydrogenase (ubiquinone) 1 alpha subcomplex 2 | 0.12 | 5.45 | 2.09 | 0.32 |
| CNAG_06404 | hypothetical protein CNAG_06404 | 0.05 | 5.44 | 2.20 | 0.12 |
| CNAG_00587 | hypothetical protein CNAG_00587 | 3.01 | 5.44 | 19.04 | 0.85 |
| CNAG_06817 | NCS2 family nucleobase:cation symporter-2 | 1.28 | 5.44 | 6.05 | 1.14 |
| CNAG_00331 | alpha beta hydrolase | 1.27 | 5.42 | 4.78 | 1.43 |
| CNAG_05631 | NADH-ubiquinone oxidoreductase 49 kDa mitochondrial | 0.07 | 5.40 | 1.29 | 0.30 |
| CNAG_05079 | hypothetical protein CNAG_05079 | 0.36 | 5.37 | 1.35 | 1.41 |
| CNAG_07476 | hypothetical protein CNAG_07476 | 1.14 | 5.34 | 4.92 | 1.22 |
| CNAG_06946 | hypothetical protein CNAG_06946 | 0.06 | 5.34 | 1.44 | 0.23 |
| CNAG_01980 | hypothetical protein CNAG_01980 | #N/A | 5.33 | #N/A | 4.96 |
| CNAG_01354 | hypothetical protein CNAG_01354 | 0.76 | 5.29 | 4.86 | 0.82 |
| CNAG_00033 | hypothetical protein CNAG_00033 | #N/A | 5.24 | 9.50 | 0.81 |
| CNAG_06180 | NADH dehydrogenase (ubiquinone) Fe-S 4 | 0.09 | 5.24 | 1.15 | 0.41 |
| CNAG_05253 | hypothetical protein CNAG_05253 | 0.21 | 5.21 | 2.62 | 0.42 |
| CNAG_05154 | membrane fraction | 0.57 | 5.20 | 5.50 | 0.54 |
| CNAG_05313 | hypothetical protein CNAG_05313 | 1.02 | 5.17 | 3.70 | 1.42 |
| CNAG_06096 | tricarboxylate carrier | 0.12 | 5.14 | 1.17 | 0.53 |
| CNAG_01846 | flavoprotein | 0.04 | 5.11 | 1.89 | 0.10 |
| CNAG_03482 | thioredoxin-dependent peroxide reductase | 1.31 | 5.10 | 4.62 | 1.44 |
| CNAG_05358 | hypothetical protein CNAG_05358 | 1.22 | 5.07 | 5.32 | 1.15 |
| CNAG_06524 | hypothetical protein CNAG_06524 | 2.50 | 5.07 | 16.25 | 0.77 |
| CNAG_02544 | DNA repair Swi5 Sae3 | 1.56 | 5.06 | 7.04 | 1.10 |
| CNAG_05090 | quinone oxidoreductase | 0.43 | 5.04 | 2.98 | 0.71 |
| CNAG_03552 | hypothetical protein CNAG_03552 | 0.77 | 5.03 | 4.90 | 0.78 |
| CNAG_00597 | amino acid transporter | 1.34 | 5.01 | 5.56 | 1.20 |
| CNAG_04056 | hypothetical protein CNAG_04056 | 0.14 | 5.01 | 1.59 | 0.44 |
| CNAG_04241 | cytoplasmic variant | 0.36 | 4.98 | 4.20 | 0.42 |
| CNAG_05170 | hypothetical protein CNAG_05170 | 0.96 | 4.94 | 2.98 | 1.57 |
| CNAG_01040 | carboxypeptidase D | 2.32 | 4.90 | 5.68 | 1.98 |
| CNAG_03481 | ribonuclease P component | 0.66 | 4.89 | 2.79 | 1.15 |
| CNAG_01076 | 4-aminobutyrate aminotransferase | 1.93 | 4.87 | 8.98 | 1.04 |
| CNAG_00654 | sulfiredoxin | 4.34 | 4.85 | 14.41 | 1.45 |
| CNAG_03654 | ATP-dependent DNA helicase | 1.07 | 4.85 | 4.91 | 1.04 |
| CNAG_02512 | DNA repair RAD16 | 1.97 | 4.84 | 8.86 | 1.06 |
| CNAG_03133 | UDP-glucose,sterol transferase | 1.41 | 4.84 | 4.58 | 1.47 |
| CNAG_05515 | hypothetical protein CNAG_05515 | 1.57 | 4.80 | 6.67 | 1.12 |
| CNAG_02589 | hypothetical protein CNAG_02589 | 2.03 | 4.76 | 9.53 | 1.01 |
| CNAG_05316 | inositol oxygenase | 0.40 | 4.75 | 3.05 | 0.62 |
| CNAG_01287 | NADH-ubiquinone oxidoreductase 51 kDa subunit | 0.05 | 4.74 | 0.98 | 0.25 |
| CNAG_02893 | hypothetical protein CNAG_02893 | 2.70 | 4.73 | 3.90 | 3.25 |
| CNAG_05258 | glucose-methanol-choline (GMC) oxidoreductase | 5.68 | 4.72 | 17.36 | 1.53 |
| CNAG_02143 | hypothetical protein CNAG_02143 | 1.64 | 4.71 | 2.99 | 2.55 |
| CNAG_02581 | hypothetical protein CNAG_02581 | 0.14 | 4.68 | 1.98 | 0.33 |
| CNAG_02463 | GTP binding and negative regulator of the Ran Tc4 GTPase cycle Gtr1p | 0.74 | 4.66 | 3.43 | 1.00 |
| CNAG_05706 | hypothetical protein CNAG_05706 | 0.12 | 4.63 | 2.08 | 0.28 |
| CNAG_05729 | hypothetical protein CNAG_05729 | #N/A | 4.63 | 2.45 | 2.85 |
| CNAG_00978 | NADH dehydrogenase (ubiquinone) 1 alpha subcomplex 9 | 0.09 | 4.62 | 1.20 | 0.34 |
| CNAG_00163 | general transcription factor 3C polypeptide 4 | 0.45 | 4.60 | 1.95 | 1.06 |
| CNAG_03128 | lincomycin-condensing lmbA | 0.33 | 4.57 | 2.47 | 0.60 |
| CNAG_02016 | hypothetical protein CNAG_02016 | 3.23 | 4.57 | 8.98 | 1.63 |
| CNAG_07844 | amino acid transporter | 2.02 | 4.55 | 10.75 | 0.85 |
| CNAG_06029 | peptidyl-tRNA hydrolase ICT1 | 0.23 | 4.54 | 2.70 | 0.38 |
| CNAG_05169 | L-lactate dehydrogenase (cytochrome) | 0.21 | 4.54 | 1.86 | 0.50 |
| CNAG_06204 | high-affinity nicotinic acid transporter | 0.71 | 4.53 | 6.99 | 0.45 |
| CNAG_05010 | hypothetical protein CNAG_05010 | 1.27 | 4.51 | 4.69 | 1.21 |
| CNAG_01470 | NADH dehydrogenase (ubiquinone) flavo 2 | 0.08 | 4.49 | 1.52 | 0.24 |
| CNAG_01118 | AAT family amino acid transporter | 1.64 | 4.48 | 5.12 | 1.42 |
| CNAG_00905 | MFS transporter | 0.92 | 4.40 | 2.06 | 1.94 |
| CNAG_05868 | glutamate-1-semialdehyde 2,1-aminomutase | 2.05 | 4.39 | 9.71 | 0.92 |
| CNAG_01144 | damaged DNA binding | 1.44 | 4.37 | 6.41 | 0.97 |
| CNAG_00433 | hypothetical protein CNAG_00433 | 1.18 | 4.33 | 3.67 | 1.38 |
| CNAG_06818 | fungal Zn(2)-Cys(6) binuclear cluster domain-containing | 0.94 | 4.33 | 3.98 | 1.01 |
| CNAG_02579 | hypothetical protein CNAG_02579 | 0.61 | 4.31 | 2.39 | 1.10 |
| CNAG_00123 | hypothetical protein CNAG_00123 | 2.15 | 4.31 | 2.15 | 4.27 |
| CNAG_06539 | monocarboxylic acid transporter | 2.43 | 4.31 | 5.32 | 1.95 |
| CNAG_03629 | NADH dehydrogenase (quinone) G subunit | 0.05 | 4.30 | 1.15 | 0.20 |
| CNAG_06503 | uridine permease | 2.83 | 4.30 | 8.70 | 1.38 |
| CNAG_03813 | replication factor A3 | 1.35 | 4.29 | 6.02 | 0.95 |
| CNAG_03912 | AE016780 membrane | 1.58 | 4.27 | 9.94 | 0.67 |
| CNAG_00010 | cation transporter | 0.37 | 4.23 | 1.69 | 0.92 |
| CNAG_03363 | hypothetical protein CNAG_03363 | 0.11 | 4.23 | 1.98 | 0.24 |
| CNAG_06555 | aromatic amino acid aminotransferase I | 0.53 | 4.23 | 2.01 | 1.10 |
| CNAG_07862 | fumarate reductase (NADH) | 0.15 | 4.23 | 2.55 | 0.24 |
| CNAG_07924 | RNA polymerase II transcription factor | 0.69 | 4.22 | 2.37 | 1.21 |
| CNAG_02028 | CMGC SRPK kinase | 0.12 | 4.22 | 1.00 | 0.51 |
| CNAG_07324 | hypothetical protein CNAG_07324 | 1.61 | 4.21 | 4.28 | 1.57 |
| CNAG_06512 | pirin domain | 1.02 | 4.21 | 5.76 | 0.74 |
| CNAG_03551 | hypothetical protein CNAG_03551 | #N/A | 4.20 | 8.42 | 0.83 |
| CNAG_02849 | glutathione transferase | 1.17 | 4.20 | 2.25 | 2.16 |
| CNAG_07177 | NADH dehydrogenase (ubiquinone) Fe-S 3 | 0.13 | 4.19 | 1.19 | 0.44 |
| CNAG_02684 | hypothetical protein CNAG_02684 | 0.95 | 4.18 | 2.89 | 1.36 |
| CNAG_00078 | vacuolar protein | 0.50 | 4.16 | 2.80 | 0.73 |
| CNAG_07661 | hypothetical protein CNAG_07661 | 1.18 | 4.16 | 4.40 | 1.11 |
| CNAG_01816 | hypothetical protein, variant | 2.11 | 4.15 | 7.85 | 1.10 |
| CNAG_04960 | hypothetical protein CNAG_04960 | 0.71 | 4.14 | 3.63 | 0.80 |
| CNAG_02387 | hypothetical protein CNAG_02387 | 0.50 | 4.12 | 2.57 | 0.80 |
| CNAG_04707 | hypothetical protein CNAG_04707 | 2.05 | 4.12 | 6.18 | 1.35 |
| CNAG_07564 | hypothetical protein CNAG_07564 | 1.58 | 4.11 | 5.48 | 1.18 |
| CNAG_06227 | fanconi-associated nuclease 1 | 1.09 | 4.10 | 4.30 | 1.03 |
| CNAG_00664 | hypothetical protein CNAG_00664 | 1.13 | 4.10 | 4.44 | 1.03 |
| CNAG_05041 | NADH-ubiquinone oxidoreductase subunit 8 | 0.06 | 4.09 | 1.12 | 0.21 |
| CNAG_06876 | alpha-ketoglutarate-dependent taurine dioxygenase | 0.95 | 4.07 | 3.19 | 1.20 |
| CNAG_05899 | pyrroline-5-carboxylate reductase | 0.41 | 4.05 | 1.41 | 1.18 |
| CNAG_02966 | carboxypeptidase D | 3.22 | 4.04 | 3.27 | 3.94 |
| CNAG_00891 | ATP-binding subfamily member 2 | 0.20 | 4.01 | 2.03 | 0.39 |
| CNAG_04837 | hypothetical protein CNAG_04837 | 1.94 | 4.00 | 4.01 | 1.91 |
| CNAG_03923 | crossover junction endonuclease MUS81 | 0.65 | 3.97 | 2.50 | 1.02 |
| CNAG_04753 | gluconolactonase | 0.81 | 3.97 | 3.24 | 0.99 |
| CNAG_00796 | ATP-binding subfamily B (MDR TAP) member 1 | 0.61 | 3.95 | 3.11 | 0.76 |
| CNAG_00247 | alpha-aminoadipic semialdehyde synthase | 1.35 | 3.95 | 1.77 | 2.98 |
| CNAG_00663 | hypothetical protein CNAG_00663 | 0.73 | 3.92 | 3.24 | 0.87 |
| CNAG_07387 | siderophore-iron transporter Str3 | 2.81 | 3.92 | 10.55 | 1.04 |
| CNAG_01229 | L-mandelate dehydrogenase | 0.13 | 3.91 | 2.05 | 0.25 |
| CNAG_05264 | alpha-amylase variant | 0.84 | 3.90 | 2.91 | 1.12 |
| CNAG_03122 | hypothetical protein CNAG_03122 | 1.64 | 3.89 | 3.46 | 1.83 |
| CNAG_05913 | alpha-glucosidase | 0.90 | 3.89 | 3.87 | 0.90 |
| CNAG_00898 | multidrug efflux pump | 2.23 | 3.88 | 7.40 | 1.15 |
| CNAG_04872 | mitochondrial protein | 0.28 | 3.88 | 1.48 | 0.74 |
| CNAG_04870 | hypothetical protein CNAG_04870 | 0.35 | 3.86 | 2.40 | 0.56 |
| CNAG_00992 | homocitrate mitochondrial | 0.48 | 3.84 | 1.39 | 1.32 |
| CNAG_01612 | SNF1 family kinase | 2.42 | 3.81 | 8.98 | 1.02 |
| CNAG_03614 | hypothetical protein CNAG_03614 | 1.83 | 3.80 | 2.76 | 2.50 |
| CNAG_07573 | DNA ligase (ATP) | 1.70 | 3.80 | 5.97 | 1.07 |
| CNAG_03395 | iron ion homeostasis-related | 0.10 | 3.78 | 1.59 | 0.25 |
| CNAG_05114 | peroxisomal copper amine oxidase | 1.69 | 3.78 | 4.32 | 1.47 |
| CNAG_04869 | carboxylesterase | 1.93 | 3.76 | 4.25 | 1.69 |
| CNAG_05330 | MFS SP general alpha glucoside:H symporter | 4.41 | 3.74 | 11.23 | 1.46 |
| CNAG_07549 | hypothetical protein CNAG_07549 | 0.31 | 3.73 | 2.94 | 0.39 |
| CNAG_00315 | HHE domain-containing | 0.12 | 3.71 | 3.31 | 0.13 |
| CNAG_06373 | mitotic spindle assembly checkpoint MAD2B | 0.41 | 3.71 | 1.24 | 1.21 |
| CNAG_06226 | NADH dehydrogenase (ubiquinone) 1 alpha subcomplex 5 | 0.10 | 3.71 | 1.52 | 0.25 |
| CNAG_04486 | hypothetical protein CNAG_04486 | 1.83 | 3.71 | 6.29 | 1.07 |
| CNAG_04085 | oxidoreductase | 1.48 | 3.70 | 4.70 | 1.16 |
| CNAG_05075 | sodium:inorganic phosphate symporter | 1.24 | 3.69 | 3.32 | 1.36 |
| CNAG_04901 | hypothetical protein CNAG_04901 | 0.31 | 3.69 | 3.22 | 0.36 |
| CNAG_02266 | NADH-quinone oxidoreductase subunit B 2 | 0.14 | 3.69 | 1.49 | 0.33 |
| CNAG_00812 | cohesin complex subunit SA-1 2 | 0.90 | 3.68 | 3.28 | 1.00 |
| CNAG_06828 | solute carrier family 36 (proton-coupled amino acid transporter) | 0.15 | 3.67 | 1.39 | 0.38 |
| CNAG_06802 | hypothetical protein CNAG_06802 | 3.84 | 3.66 | 10.58 | 1.31 |
| CNAG_00451 | cytoplasmic protein | 0.70 | 3.65 | 3.12 | 0.82 |
| CNAG_02765 | 3-hydroxyisobutyrate dehydrogenase | 0.98 | 3.65 | 5.49 | 0.64 |
| CNAG_06188 | hypothetical protein CNAG_06188 | 0.76 | 3.65 | 2.84 | 0.97 |
| CNAG_06027 | aryl-alcohol dehydrogenase | 2.26 | 3.64 | 7.58 | 1.07 |
| CNAG_07802 | class III aminotransferase | 9.69 | 3.61 | 18.53 | 1.87 |
| CNAG_03637 | damaged DNA binding | 1.09 | 3.61 | 3.71 | 1.05 |
| CNAG_05070 | sulfite reductase (NADPH) beta-component | 0.14 | 3.60 | 2.13 | 0.23 |
| CNAG_01316 | replication factor A2 | 0.86 | 3.59 | 3.26 | 0.94 |
| CNAG_05112 | hypothetical protein CNAG_05112 | 0.44 | 3.59 | 2.26 | 0.70 |
| CNAG_00545 | cohesin loading factor subunit SCC2 | 0.84 | 3.57 | 2.82 | 1.05 |
| CNAG_01725 | hypothetical protein CNAG_01725 | 1.38 | 3.55 | 3.42 | 1.42 |
| CNAG_03659 | DNA replication regulator DPB11 | 1.09 | 3.55 | 3.51 | 1.09 |
| CNAG_03101 | efflux protein EncT | 1.67 | 3.54 | 3.66 | 1.59 |
| CNAG_06573 | hypothetical protein CNAG_06573 | 1.02 | 3.52 | 3.03 | 1.17 |
| CNAG_02715 | alpha-1,6-mannosyltransferase | 1.07 | 3.52 | 3.15 | 1.18 |
| CNAG_03206 | endonuclease III | 0.23 | 3.50 | 1.89 | 0.42 |
| CNAG_07361 | hypothetical protein CNAG_07361 | 0.22 | 3.49 | 1.72 | 0.44 |
| CNAG_04504 | hypothetical protein CNAG_04504 | 1.06 | 3.49 | 4.09 | 0.90 |
| CNAG_02586 | sugar transporter | 2.15 | 3.48 | 5.42 | 1.37 |
| CNAG_05578 | hypothetical protein CNAG_05578 | 0.21 | 3.48 | 2.05 | 0.36 |
| CNAG_02317 | arginine N-methyltransferase 3 | 0.26 | 3.47 | 2.34 | 0.38 |
| CNAG_03167 | CAMK CAMKL Chk1 kinase | 1.34 | 3.47 | 4.78 | 0.97 |
| CNAG_02043 | hypothetical protein CNAG_02043 | 1.54 | 3.46 | 2.85 | 1.86 |
| CNAG_00536 | hypothetical protein CNAG_00536 | 0.24 | 3.44 | 2.20 | 0.37 |
| CNAG_04631 | ribitol kinase | 0.60 | 3.42 | 2.62 | 0.78 |
| CNAG_01163 | DNA repair and recombination RAD54 | 1.24 | 3.42 | 3.98 | 1.06 |
| CNAG_07691 | hypothetical protein CNAG_07691 | 1.36 | 3.41 | 4.35 | 1.05 |
| CNAG_07938 | hypothetical protein CNAG_07938 | 0.78 | 3.41 | 2.45 | 1.08 |
| CNAG_06593 | rhamnogalacturonan lyase | 1.60 | 3.41 | 4.67 | 1.16 |
| CNAG_06311 | cytoplasmic variant | 1.38 | 3.41 | 2.99 | 1.56 |
| CNAG_03461 | hypothetical protein CNAG_03461 | 2.31 | 3.40 | 7.54 | 1.03 |
| CNAG_01244 | hypothetical protein CNAG_01244 | 2.30 | 3.39 | 4.81 | 1.61 |
| CNAG_02062 | glycoside hydrolase family 2 | 0.64 | 3.39 | 2.78 | 0.78 |
| CNAG_04220 | ATP-dependent DNA helicase II subunit 1 | 1.00 | 3.38 | 3.38 | 0.99 |
| CNAG_06743 | mitochondrial genome maintenance-related | 1.36 | 3.37 | 4.72 | 0.97 |
| CNAG_01953 | hypothetical protein CNAG_01953 | 1.38 | 3.37 | 2.94 | 1.56 |
| CNAG_03927 | hypothetical protein CNAG_03927 | 0.27 | 3.36 | 2.15 | 0.42 |
| CNAG_00843 | salicylate hydroxylase | 1.12 | 3.36 | 3.72 | 1.00 |
| CNAG_04271 | hypothetical protein CNAG_04271 | 0.72 | 3.33 | 2.35 | 1.01 |
| CNAG_00869 | ATP-binding cassette (ABC) transporter | 0.62 | 3.30 | 2.89 | 0.71 |
| CNAG_05449 | hypothetical protein CNAG_05449 | 0.62 | 3.29 | 3.42 | 0.59 |
| CNAG_05404 | histone-lysine n-methyltransferase | 0.46 | 3.29 | 1.58 | 0.95 |
| CNAG_04471 | FAD dependent oxidoreductase superfamily | 1.36 | 3.28 | 4.36 | 1.02 |
| CNAG_04675 | hypothetical protein CNAG_04675 | 2.74 | 3.27 | 5.17 | 1.72 |
| CNAG_08014 | hypothetical protein CNAG_08014 | 0.88 | 3.25 | 3.22 | 0.88 |
| CNAG_01527 | hypothetical protein CNAG_01527 | 1.31 | 3.24 | 3.14 | 1.34 |
| CNAG_03183 | nuclear protein | 0.14 | 3.23 | 1.13 | 0.40 |
| CNAG_05881 | alpha-1,2-mannosyltransferase | 0.27 | 3.23 | 1.90 | 0.45 |
| CNAG_02968 | hypothetical protein CNAG_02968 | 1.63 | 3.22 | 3.88 | 1.34 |
| CNAG_02204 | endonuclease mitochondrial | 0.26 | 3.22 | 2.07 | 0.40 |
| CNAG_02540 | hypothetical protein CNAG_02540 | 1.57 | 3.22 | 1.50 | 3.34 |
| CNAG_06904 | hypothetical protein CNAG_06904 | 1.34 | 3.21 | 2.02 | 2.11 |
| CNAG_06558 | NADPH-ferrihemo reductase | 0.81 | 3.20 | 1.82 | 1.41 |
| CNAG_07909 | meiotic recombinase Dmc1 | 0.68 | 3.20 | 3.38 | 0.64 |
| CNAG_01323 | ubiquinol-cytochrome c reductase subunit 7 | 0.27 | 3.20 | 1.96 | 0.43 |
| CNAG_03544 | hypothetical protein CNAG_03544 | 0.20 | 3.19 | 1.47 | 0.43 |
| CNAG_05909 | cytochrome heme mitochondrial | 0.12 | 3.18 | 1.18 | 0.32 |
| CNAG_03934 | hypothetical protein CNAG_03934 | #N/A | 3.17 | 5.98 | 1.02 |
| CNAG_03910 | D-xylose-proton symporter | 1.18 | 3.15 | 3.89 | 0.95 |
| CNAG_04546 | multidrug transporter | 2.29 | 3.14 | 6.17 | 1.16 |
| CNAG_04759 | hypothetical protein CNAG_04759 | 2.03 | 3.14 | 3.94 | 1.60 |
| CNAG_03085 | hypothetical protein CNAG_03085 | 2.04 | 3.12 | 2.70 | 2.33 |
| CNAG_02549 | hypothetical protein CNAG_02549 | 1.00 | 3.12 | 3.16 | 0.98 |
| CNAG_05115 | sarcosine oxidase | 0.63 | 3.12 | 2.82 | 0.69 |
| CNAG_01944 | hypothetical protein CNAG_01944 | 0.56 | 3.12 | 1.64 | 1.06 |
| CNAG_06642 | Atypical PIKK FRAP kinase | 0.66 | 3.12 | 1.88 | 1.08 |
| CNAG_05016 | hypothetical protein CNAG_05016 | 1.82 | 3.11 | 4.00 | 1.40 |
| CNAG_02580 | hypothetical protein CNAG_02580 | 0.45 | 3.11 | 2.57 | 0.53 |
| CNAG_01802 | Fe-S cluster assembly DRE2 | 0.50 | 3.10 | 2.41 | 0.63 |
| CNAG_07188 | hypothetical protein CNAG_07188 | 1.72 | 3.10 | 4.35 | 1.22 |
| CNAG_05138 | glucan 1,3-beta-glucosidase | 1.42 | 3.10 | 3.97 | 1.09 |
| CNAG_01545 | hypothetical protein, variant | 0.30 | 3.07 | 2.35 | 0.39 |
| CNAG_03755 | hypothetical protein CNAG_03755 | 0.23 | 3.06 | 1.98 | 0.36 |
| CNAG_04599 | 3-methyl-2-oxobutanoate hydroxymethyltransferase | 0.27 | 3.06 | 1.74 | 0.46 |
| CNAG_02047 | hypothetical protein CNAG_02047 | 1.65 | 3.05 | 4.84 | 1.03 |
| CNAG_03166 | hypothetical protein CNAG_03166 | 1.58 | 3.05 | 4.01 | 1.19 |
| CNAG_04202 | cytosolic Fe-S cluster assembly factor NAR1 | 0.15 | 3.04 | 1.61 | 0.28 |
| CNAG_04768 | hypothetical protein CNAG_04768 | 0.38 | 3.04 | 1.73 | 0.66 |
| CNAG_05267 | NADH dehydrogenase (ubiquinone) Fe-S 5 | 0.21 | 3.03 | 1.34 | 0.47 |
| CNAG_02221 | solute carrier family 39 (zinc transporter) member 9 | 0.30 | 3.03 | 1.53 | 0.59 |
| CNAG_05508 | hypothetical protein CNAG_05508 | 1.20 | 3.03 | 3.71 | 0.97 |
| CNAG_00022 | cytochrome c heme-lyase | 0.15 | 3.03 | 1.66 | 0.28 |
| CNAG_02892 | phosphatidylinositol class B | 1.63 | 3.03 | 4.38 | 1.12 |
| CNAG_03663 | L-lactate dehydrogenase | 0.10 | 3.02 | 1.31 | 0.24 |
| CNAG_07869 | hypothetical protein CNAG_07869 | 3.18 | 3.00 | 4.11 | 2.30 |
| CNAG_02140 | NADH dehydrogenase (ubiquinone) 1 alpha subcomplex 12 | 0.21 | 3.00 | 1.61 | 0.38 |
| CNAG_04446 | hypothetical protein CNAG_04446 | 0.65 | 3.00 | 3.15 | 0.62 |
| CNAG_04681 | hypothetical protein CNAG_04681 | 1.64 | 2.99 | 3.25 | 1.50 |
| CNAG_00627 | specific transcriptional repressor | 1.96 | 2.97 | 4.35 | 1.33 |
| CNAG_03695 | hypothetical protein CNAG_03695 | 1.14 | 2.97 | 3.50 | 0.96 |
| CNAG_05867 | L-fucose transporter | 0.97 | 2.97 | 2.22 | 1.28 |
| CNAG_07314 | hypothetical protein CNAG_07314 | 1.94 | 2.97 | 3.75 | 1.52 |
| CNAG_04689 | hypothetical protein CNAG_04689 | 0.49 | 2.96 | 3.30 | 0.43 |
| CNAG_03386 | solute carrier family 25 (mitochondrial carnitine acylcarnitine transporter) member 20 29 | 0.85 | 2.96 | 1.22 | 2.03 |
| CNAG_00328 | single-stranded DNA specific endodeoxyribonuclease | 0.52 | 2.96 | 1.60 | 0.95 |
| CNAG_04975 | hypothetical protein CNAG_04975 | 0.80 | 2.96 | 1.56 | 1.50 |
| CNAG_03830 | hypothetical protein CNAG_03830 | 1.45 | 2.95 | 4.85 | 0.88 |
| CNAG_03781 | hypothetical protein CNAG_03781 | 0.91 | 2.95 | 2.62 | 1.02 |
| CNAG_04725 | hypothetical protein CNAG_04725 | 0.86 | 2.94 | 2.40 | 1.04 |
| CNAG_02826 | amino-acid mitochondrial | 0.84 | 2.94 | 1.99 | 1.23 |
| CNAG_07911 | streptomycin biosynthesis | 0.19 | 2.93 | 2.58 | 0.21 |
| CNAG_05660 | hypothetical protein CNAG_05660 | 1.00 | 2.93 | 2.73 | 1.07 |
| CNAG_03767 | cohesin complex subunit psm1 | 0.77 | 2.93 | 2.18 | 1.02 |
| CNAG_04392 | sterol-binding protein | 1.33 | 2.92 | 2.99 | 1.29 |
| CNAG_00269 | sorbitol dehydrogenase | 1.68 | 2.92 | 4.14 | 1.17 |
| CNAG_00276 | hypothetical protein CNAG_00276 | 1.83 | 2.92 | 4.21 | 1.26 |
| CNAG_00498 | cell division cycle 14 | 1.51 | 2.92 | 3.71 | 1.18 |
| CNAG_05501 | hypothetical protein, variant | 1.71 | 2.91 | 5.41 | 0.91 |
| CNAG_01167 | chromosome associated | 0.92 | 2.91 | 2.43 | 1.09 |
| CNAG_02039 | integral to membrane | 1.84 | 2.91 | 2.20 | 2.41 |
| CNAG_03966 | hypothetical protein CNAG_03966 | 0.28 | 2.91 | 1.64 | 0.50 |
| CNAG_02203 | karyopherin importin that interacts with the nuclear pore complex | 0.21 | 2.90 | 1.13 | 0.53 |
| CNAG_07572 | elongator complex 3 | 0.27 | 2.90 | 1.76 | 0.44 |
| CNAG_00144 | hypothetical protein CNAG_00144 | 2.17 | 2.90 | 3.09 | 2.01 |
| CNAG_03154 | hypothetical protein CNAG_03154 | 0.55 | 2.90 | 2.22 | 0.71 |
| CNAG_00321 | dRaptor | 1.50 | 2.89 | 3.61 | 1.19 |
| CNAG_02490 | meiotic DNA double-strand break processing-related | 1.07 | 2.88 | 2.75 | 1.11 |
| CNAG_00653 | hypothetical protein CNAG_00653 | 1.82 | 2.87 | 3.64 | 1.42 |
| CNAG_00306 | hypothetical protein CNAG_00306 | 0.70 | 2.87 | 2.35 | 0.84 |
| CNAG_05612 | hypothetical protein CNAG_05612 | 1.31 | 2.86 | 3.82 | 0.97 |
| CNAG_07388 | hypothetical protein CNAG_07388 | 5.24 | 2.86 | 9.64 | 1.53 |
| CNAG_00176 | glutamate carboxypeptidase | 0.26 | 2.85 | 0.98 | 0.74 |
| CNAG_07386 | GTP binding and negative regulator of the Ran Tc4 GTPase cycle Gtr1p | 1.00 | 2.85 | 2.50 | 1.13 |
| CNAG_04735 | extracellular elastinolytic metallo ase | 1.88 | 2.85 | 3.66 | 1.45 |
| CNAG_03666 | acyl- dehydrogenase | 0.06 | 2.83 | 1.16 | 0.14 |
| CNAG_07512 | hypothetical protein CNAG_07512 | 2.90 | 2.83 | 6.21 | 1.31 |
| CNAG_06715 | hypothetical protein CNAG_06715 | 1.36 | 2.82 | 3.56 | 1.06 |
| CNAG_00754 | ATP-binding sub-family member 1 | 0.13 | 2.82 | 1.38 | 0.27 |
| CNAG_00299 | DNA repair RAD5 | 0.75 | 2.82 | 2.19 | 0.96 |
| CNAG_02596 | hypothetical protein CNAG_02596 | 1.76 | 2.82 | 4.45 | 1.10 |
| CNAG_00933 | hypothetical protein CNAG_00933 | 2.01 | 2.82 | 3.99 | 1.41 |
| CNAG_03436 | alanine transaminase | 0.33 | 2.81 | 0.90 | 1.03 |
| CNAG_04201 | hypothetical protein CNAG_04201 | 1.54 | 2.81 | 2.41 | 1.78 |
| CNAG_04713 | citrate lyase subunit beta | 0.48 | 2.80 | 1.70 | 0.78 |
| CNAG_03543 | hypothetical protein CNAG_03543 | 0.43 | 2.80 | 1.85 | 0.65 |
| CNAG_01601 | lipase | 1.58 | 2.79 | 3.25 | 1.34 |
| CNAG_00425 | hypothetical protein CNAG_00425 | 1.08 | 2.78 | 1.63 | 1.82 |
| CNAG_05300 | mitochondrial carrier | 0.90 | 2.78 | 2.14 | 1.16 |
| CNAG_01952 | aryl-alcohol dehydrogenase | 1.45 | 2.78 | 3.43 | 1.17 |
| CNAG_05318 | L-mandelate dehydrogenase | 0.41 | 2.78 | 1.84 | 0.61 |
| CNAG_05514 | hypothetical protein CNAG_05514 | 3.18 | 2.78 | 5.92 | 1.47 |
| CNAG_02343 | hypothetical protein CNAG_02343 | 1.11 | 2.76 | 3.18 | 0.95 |
| CNAG_06517 | C2 domain | 1.14 | 2.76 | 2.22 | 1.41 |
| CNAG_02448 | hypothetical protein CNAG_02448 | 1.93 | 2.75 | 4.14 | 1.27 |
| CNAG_05820 | aromatic-amino-acid transaminase | 1.02 | 2.75 | 1.24 | 2.24 |
| CNAG_02738 | hypothetical protein CNAG_02738 | 0.36 | 2.74 | 2.02 | 0.49 |
| CNAG_07698 | hypothetical protein CNAG_07698 | 0.73 | 2.74 | 2.53 | 0.79 |
| CNAG_07943 | hypothetical protein CNAG_07943 | 1.30 | 2.74 | 3.42 | 1.03 |
| CNAG_07782 | oxidoreductase | 0.68 | 2.74 | 2.13 | 0.86 |
| CNAG_00799 | cellulase | 0.68 | 2.74 | 1.92 | 0.97 |
| CNAG_01577 | glutamate dehydrogenase (NADP+) | 0.27 | 2.73 | 1.33 | 0.55 |
| CNAG_06901 | UDP-N-acetylglucosamine-dolichyl-phosphate N-acetylglucosaminephosphotransferase | 0.92 | 2.73 | 2.72 | 0.92 |
| CNAG_05861 | hypothetical protein CNAG_05861 | 1.53 | 2.72 | 2.83 | 1.46 |
| CNAG_05602 | 1-pyrroline-5-carboxylate dehydrogenase | 0.75 | 2.72 | 1.49 | 1.35 |
| CNAG_01983 | cAMP-independent regulatory | 0.94 | 2.71 | 1.94 | 1.30 |
| CNAG_05755 | Glutathione S-transferase 6 | 0.30 | 2.71 | 2.14 | 0.37 |
| CNAG_03476 | spermidine synthase | 0.49 | 2.71 | 1.16 | 1.13 |
| CNAG_04717 | hypothetical protein CNAG_04717 | 2.20 | 2.71 | 3.96 | 1.49 |
| CNAG_04472 | membrane protein | 1.63 | 2.70 | 3.87 | 1.12 |
| CNAG_02305 | hypothetical protein CNAG_02305 | 0.60 | 2.70 | 1.94 | 0.82 |
| CNAG_01836 | long-chain acyl- synthetase | 2.14 | 2.70 | 3.69 | 1.55 |
| CNAG_07933 | hypothetical protein CNAG_07933 | 1.17 | 2.69 | 3.18 | 0.98 |
| CNAG_04595 | hypothetical protein CNAG_04595 | 0.38 | 2.69 | 1.28 | 0.80 |
| CNAG_04648 | sister chromatid cohesion PDS5 | 0.84 | 2.69 | 1.94 | 1.15 |
| CNAG_02299 | hypothetical protein CNAG_02299 | 1.69 | 2.68 | 4.52 | 0.99 |
| CNAG_02318 | hypothetical protein CNAG_02318 | 0.62 | 2.66 | 1.60 | 1.03 |
| CNAG_06897 | hypothetical protein CNAG_06897 | 0.91 | 2.65 | 1.98 | 1.21 |
| CNAG_06554 | FAD dependent oxidoreductase superfamily | 1.74 | 2.63 | 3.14 | 1.44 |
| CNAG_05379 | regucalcin | 0.45 | 2.62 | 1.39 | 0.84 |
| CNAG_05365 | ribose-phosphate pyrophosphokinase | 0.36 | 2.62 | 1.82 | 0.51 |
| CNAG_07758 | hypothetical protein CNAG_07758 | 1.04 | 2.62 | 2.75 | 0.99 |
| CNAG_04476 | hypothetical protein CNAG_04476 | 1.65 | 2.62 | 3.65 | 1.17 |
| CNAG_01721 | porphobilinogen deaminase | 0.15 | 2.62 | 1.71 | 0.22 |
| CNAG_06008 | asparaginase | 0.83 | 2.62 | 1.69 | 1.27 |
| CNAG_03838 | MFS transporter | 2.03 | 2.61 | 2.42 | 2.17 |
| CNAG_05907 | pyruvate carboxylase | 0.35 | 2.61 | 1.18 | 0.76 |
| CNAG_00883 | transcription factor | 0.77 | 2.60 | 1.92 | 1.03 |
| CNAG_03411 | hypothetical protein CNAG_03411 | 1.13 | 2.59 | 3.12 | 0.93 |
| CNAG_01856 | hypothetical protein CNAG_01856 | 1.92 | 2.59 | 4.70 | 1.05 |
| CNAG_06777 | fructosyl amino acid oxidase | 1.13 | 2.59 | 1.94 | 1.50 |
| CNAG_00936 | lipid particle | 0.85 | 2.58 | 2.08 | 1.05 |
| CNAG_00329 | NADH dehydrogenase (ubiquinone) 1 beta subcomplex 8 | 0.20 | 2.58 | 1.06 | 0.48 |
| CNAG_05250 | telomere maintenance | 0.76 | 2.57 | 1.70 | 1.14 |
| CNAG_07912 | hypothetical protein CNAG_07912 | 0.56 | 2.57 | 1.96 | 0.73 |
| CNAG_06815 | hypothetical protein CNAG_06815 | 1.29 | 2.56 | 3.10 | 1.06 |
| CNAG_05821 | hypothetical protein CNAG_05821 | 1.46 | 2.55 | 2.82 | 1.31 |
| CNAG_06147 | hypothetical protein CNAG_06147 | 0.19 | 2.55 | 0.96 | 0.49 |
| CNAG_02660 | hypothetical protein CNAG_02660 | 2.99 | 2.55 | 5.17 | 1.46 |
| CNAG_04871 | hypothetical protein CNAG_04871 | 0.82 | 2.55 | 1.98 | 1.04 |
| CNAG_04576 | hypothetical protein CNAG_04576 | 1.46 | 2.54 | 2.73 | 1.34 |
| CNAG_00972 | hypothetical protein CNAG_00972 | 0.15 | 2.54 | 1.60 | 0.24 |
| CNAG_02600 | tartrate transporter | 0.64 | 2.54 | 2.31 | 0.70 |
| CNAG_04612 | hypothetical protein CNAG_04612 | 0.14 | 2.54 | 0.84 | 0.42 |
| CNAG_04968 | hypothetical protein CNAG_04968 | 0.32 | 2.54 | 1.63 | 0.49 |
| CNAG_03772 | glucose transporter | 2.00 | 2.54 | 3.90 | 1.29 |
| CNAG_04153 | hypothetical protein CNAG_04153 | 1.65 | 2.53 | 4.50 | 0.92 |
| CNAG_00791 | hypothetical protein CNAG_00791 | 1.09 | 2.53 | 2.06 | 1.33 |
| CNAG_06403 | hypothetical protein CNAG_06403 | 0.67 | 2.53 | 1.85 | 0.90 |
| CNAG_06009 | cyclohydrolase | 2.28 | 2.52 | 2.78 | 2.05 |
| CNAG_03939 | 5-aminolevulinic acid synthase | 0.19 | 2.52 | 1.10 | 0.42 |
| CNAG_03578 | hypothetical protein CNAG_03578 | 0.40 | 2.51 | 2.32 | 0.43 |
| CNAG_03252 | hypothetical protein CNAG_03252 | #N/A | 2.51 | 4.43 | 1.44 |
| CNAG_05329 | myo-inositol 2-dehydrogenase | 3.31 | 2.51 | 6.79 | 1.21 |
| CNAG_04307 | urate oxidase | 3.12 | 2.49 | 4.42 | 1.74 |
| CNAG_01936 | sugar transporter | 1.86 | 2.49 | 2.27 | 2.01 |
| CNAG_00877 | adenylate kinase | 0.34 | 2.49 | 1.25 | 0.67 |
| CNAG_05002 | hypothetical protein CNAG_05002 | 2.14 | 2.48 | 3.83 | 1.37 |
| CNAG_01681 | cytosine permease | 0.60 | 2.47 | 1.61 | 0.92 |
| CNAG_02315 | ubiquinol-cytochrome c iron-sulfur subunit | 0.22 | 2.47 | 1.30 | 0.42 |
| CNAG_06529 | hypothetical protein CNAG_06529 | 1.28 | 2.47 | 2.38 | 1.32 |
| CNAG_00830 | hypothetical protein CNAG_00830 | 0.43 | 2.47 | 1.11 | 0.96 |
| CNAG_03504 | hypothetical protein CNAG_03504 | 1.34 | 2.47 | 2.97 | 1.10 |
| CNAG_06486 | hypothetical protein CNAG_06486 | 0.96 | 2.46 | 2.44 | 0.96 |
| CNAG_02311 | hypothetical protein CNAG_02311 | 1.15 | 2.45 | 2.12 | 1.32 |
| CNAG_00800 | nicotinamidase | 0.12 | 2.44 | 1.38 | 0.21 |
| CNAG_06079 | proliferating cell nuclear antigen (pcna) | 0.62 | 2.44 | 1.78 | 0.84 |
| CNAG_05244 | hypothetical protein CNAG_05244 | 1.47 | 2.44 | 2.01 | 1.77 |
| CNAG_01960 | efflux protein EncT | 2.94 | 2.44 | 4.55 | 1.56 |
| CNAG_03445 | hypothetical protein CNAG_03445 | 1.42 | 2.44 | 2.68 | 1.28 |
| CNAG_05803 | exo-beta-1,3-glucanase | 1.06 | 2.43 | 2.71 | 0.94 |
| CNAG_06372 | hypothetical protein CNAG_06372 | 2.85 | 2.43 | 5.54 | 1.24 |
| CNAG_01603 | hypothetical protein CNAG_01603 | 2.68 | 2.43 | 3.80 | 1.70 |
| CNAG_01443 | hypothetical protein CNAG_01443 | 0.15 | 2.42 | 1.19 | 0.29 |
| CNAG_03764 | integral to membrane | 1.66 | 2.42 | 4.83 | 0.82 |
| CNAG_00389 | mitochondrial protein | 0.25 | 2.42 | 1.17 | 0.52 |
| CNAG_02598 | chitinase | 0.68 | 2.42 | 2.42 | 0.68 |
| CNAG_06751 | hypothetical protein CNAG_06751 | 0.99 | 2.41 | 2.09 | 1.13 |
| CNAG_01994 | hypothetical protein CNAG_01994 | 3.32 | 2.40 | 4.88 | 1.62 |
| CNAG_03713 | efflux variant | 1.87 | 2.40 | 3.05 | 1.46 |
| CNAG_02935 | malonic semialdehyde reductase | 0.59 | 2.40 | 1.56 | 0.90 |
| CNAG_07685 | UMF1 family MFS transporter | 0.37 | 2.40 | 1.66 | 0.53 |
| CNAG_04796 | serine threonine- phosphatase 2B catalytic subunit A1 | 0.29 | 2.40 | 1.15 | 0.60 |
| CNAG_01078 | Aldehyde dehydrogenase (ALDDH) | 1.51 | 2.39 | 3.00 | 1.20 |
| CNAG_00798 | hypothetical protein CNAG_00798 | 1.98 | 2.39 | 3.14 | 1.49 |
| CNAG_01223 | hypothetical protein CNAG_01223 | 2.18 | 2.39 | 4.50 | 1.15 |
| CNAG_06136 | hypothetical protein CNAG_06136 | 0.96 | 2.39 | 1.20 | 1.89 |
| CNAG_02319 | hypothetical protein CNAG_02319 | 1.07 | 2.39 | 2.86 | 0.89 |
| CNAG_03369 | Wee kinase | 0.78 | 2.38 | 2.32 | 0.79 |
| CNAG_01237 | hypothetical protein CNAG_01237 | 0.36 | 2.38 | 1.29 | 0.66 |
| CNAG_04908 | hypothetical protein CNAG_04908 | 0.84 | 2.38 | 1.70 | 1.17 |
| CNAG_05144 | carbonic anhydrase | 0.17 | 2.38 | 0.85 | 0.47 |
| CNAG_01635 | hypothetical protein CNAG_01635 | 0.72 | 2.37 | 1.87 | 0.91 |
| CNAG_04846 | membrane protein | 0.47 | 2.37 | 1.84 | 0.60 |
| CNAG_07625 | plasma membrane | 0.35 | 2.37 | 1.71 | 0.48 |
| CNAG_02508 | hypothetical protein CNAG_02508 | 2.47 | 2.37 | 1.19 | 4.85 |
| CNAG_05595 | hypothetical protein CNAG_05595 | 0.85 | 2.36 | 3.41 | 0.59 |
| CNAG_05523 | hypothetical protein CNAG_05523 | 1.63 | 2.35 | 3.73 | 1.02 |
| CNAG_01424 | myosin heavy chain (Zipper ) (Myosin II) (Non-muscle MHC) | 1.29 | 2.35 | 2.39 | 1.26 |
| CNAG_05818 | chitin synthase | 1.45 | 2.35 | 2.70 | 1.25 |
| CNAG_03076 | hypothetical protein CNAG_03076 | 1.44 | 2.35 | 2.39 | 1.41 |
| CNAG_02759 | hypothetical protein CNAG_02759 | 2.33 | 2.35 | 4.22 | 1.28 |
| CNAG_00911 | phytanoyl- dioxygenase | 1.16 | 2.34 | 2.80 | 0.96 |
| CNAG_05997 | hypothetical protein CNAG_05997 | 0.28 | 2.34 | 1.41 | 0.45 |
| CNAG_00768 | hypothetical protein CNAG_00768 | 1.35 | 2.34 | 2.93 | 1.06 |
| CNAG_06932 | sugar transporter | 1.13 | 2.33 | 6.17 | 0.42 |
| CNAG_05299 | oxidoreductase | 1.85 | 2.33 | 3.23 | 1.32 |
| CNAG_03304 | hypothetical protein CNAG_03304 | 0.56 | 2.33 | 1.85 | 0.69 |
| CNAG_02990 | nuclear protein | 0.64 | 2.32 | 1.29 | 1.13 |
| CNAG_02436 | hypothetical protein CNAG_02436 | 1.14 | 2.32 | 2.32 | 1.12 |
| CNAG_04891 | hypothetical protein CNAG_04891 | 1.93 | 2.32 | 8.01 | 0.55 |
| CNAG_00780 | hypothetical protein CNAG_00780 | 0.88 | 2.32 | 1.80 | 1.13 |
| CNAG_07751 | siderophore iron transporter | 3.63 | 2.32 | 8.35 | 1.00 |
| CNAG_02360 | hypothetical protein CNAG_02360 | 1.34 | 2.31 | 3.22 | 0.96 |
| CNAG_05685 | neutral amino acid transporter | 0.99 | 2.31 | 2.24 | 1.01 |
| CNAG_00155 | hypothetical protein CNAG_00155 | #N/A | 2.31 | #N/A | 1.88 |
| CNAG_02758 | NADH:flavin oxidoreductase NADH oxidase | 1.52 | 2.31 | 3.99 | 0.87 |
| CNAG_05197 | NADH dehydrogenase (ubiquinone) 1 alpha subcomplex 8 | 0.29 | 2.31 | 1.35 | 0.50 |
| CNAG_01592 | Hypothetical protein | 1.41 | 2.30 | 4.33 | 0.74 |
| CNAG_04302 | ER to Golgi transport-related | 1.27 | 2.29 | 2.70 | 1.07 |
| CNAG_01533 | rho GTPase activator | 0.69 | 2.29 | 1.53 | 1.03 |
| CNAG_04885 | phytanoyl- dioxygenase | 0.72 | 2.29 | 2.01 | 0.81 |
| CNAG_04982 | cytosine-purine permease | 1.02 | 2.29 | 2.25 | 1.03 |
| CNAG_04221 | 6-phosphofructo-2-kinase fructose-2,6-bisphosphatase | 0.35 | 2.28 | 1.10 | 0.72 |
| CNAG_01883 | hypothetical protein CNAG_01883 | 2.32 | 2.28 | 3.22 | 1.62 |
| CNAG_07936 | hypothetical protein CNAG_07936 | 1.16 | 2.28 | 2.36 | 1.11 |
| CNAG_00749 | alternative sulfate transporter | 0.53 | 2.28 | 2.84 | 0.42 |
| CNAG_03057 | hypothetical protein CNAG_03057 | 1.36 | 2.28 | 2.97 | 1.03 |
| CNAG_00476 | hypothetical protein CNAG_00476 | 1.27 | 2.27 | 4.87 | 0.59 |
| CNAG_06329 | high-affinity nicotinic acid transporter | 1.24 | 2.27 | 2.64 | 1.06 |
| CNAG_01772 | hypothetical protein CNAG_01772 | 0.20 | 2.26 | 1.52 | 0.30 |
| CNAG_04773 | hypothetical protein CNAG_04773 | 2.14 | 2.26 | 3.59 | 1.33 |
| CNAG_04736 | hypothetical protein CNAG_04736 | 1.11 | 2.26 | 2.82 | 0.88 |
| CNAG_01077 | hypothetical protein CNAG_01077 | 2.04 | 2.25 | 3.27 | 1.39 |
| CNAG_00997 | hypothetical protein CNAG_00997 | 0.23 | 2.25 | 1.37 | 0.37 |
| CNAG_05302 | amine oxidase | 2.32 | 2.25 | 2.86 | 1.80 |
| CNAG_02125 | hypothetical protein CNAG_02125 | 1.85 | 2.25 | 2.57 | 1.60 |
| CNAG_03444 | hypothetical protein CNAG_03444 | 1.46 | 2.24 | 3.05 | 1.06 |
| CNAG_01596 | hypothetical protein CNAG_01596 | 0.19 | 2.24 | 1.15 | 0.37 |
| CNAG_03589 | adrenodoxin-type ferredoxin | 0.54 | 2.24 | 2.22 | 0.54 |
| CNAG_00492 | hypothetical protein CNAG_00492 | 1.72 | 2.23 | 3.09 | 1.23 |
| CNAG_04474 | monocarboxylic acid transporter | 1.82 | 2.23 | 4.18 | 0.96 |
| CNAG_07687 | hypothetical protein CNAG_07687 | 1.53 | 2.23 | 2.35 | 1.44 |
| CNAG_06414 | cytochrome c oxidase-assembly factor mitochondrial | 0.64 | 2.22 | 1.98 | 0.71 |
| CNAG_03160 | DNA cross-link repair 1A | 1.55 | 2.22 | 2.78 | 1.23 |
| CNAG_07858 | hypothetical protein CNAG_07858 | #N/A | 2.22 | #N/A | 1.92 |
| CNAG_01963 | CMP dCMP deaminase zinc-binding | 1.76 | 2.22 | 3.90 | 0.99 |
| CNAG_02834 | UDP-glucose:sterol glucosyltransferase | 1.81 | 2.22 | 3.00 | 1.32 |
| CNAG_02532 | D-amino-acid oxidase | 1.14 | 2.21 | 2.07 | 1.20 |
| CNAG_05880 | hypothetical protein CNAG_05880 | 0.34 | 2.21 | 1.55 | 0.48 |
| CNAG_02418 | asparagine-tRNA ligase | 0.71 | 2.20 | 0.59 | 2.64 |
| CNAG_02564 | tRNA-splicing endonuclease subunit Sen54 | 0.37 | 2.20 | 1.70 | 0.47 |
| CNAG_02131 | iron-sulfur cluster assembly | 0.32 | 2.20 | 1.58 | 0.44 |
| CNAG_03664 | high-affinity nickel-transporter | 1.11 | 2.19 | 2.45 | 0.98 |
| CNAG_07356 | succinate cytochrome b556 subunit | 0.16 | 2.19 | 1.13 | 0.32 |
| CNAG_01023 | nuclear cohesin complex | 0.94 | 2.19 | 1.88 | 1.08 |
| CNAG_01964 | OPT family small oligopeptide transporter | 0.91 | 2.19 | 2.00 | 0.98 |
| CNAG_07672 | hypothetical protein CNAG_07672 | 0.64 | 2.18 | 2.32 | 0.59 |
| CNAG_04073 | hypothetical protein CNAG_04073 | 0.31 | 2.18 | 1.31 | 0.52 |
| CNAG_00904 | aflatoxin efflux pump AFLT | 0.61 | 2.17 | 1.63 | 0.80 |
| CNAG_02409 | hypothetical protein CNAG_02409 | 1.23 | 2.17 | 2.40 | 1.10 |
| CNAG_07423 | hypothetical protein CNAG_07423 | 1.32 | 2.17 | 2.91 | 0.97 |
| CNAG_01993 | hypothetical protein CNAG_01993 | 1.42 | 2.17 | 2.16 | 1.41 |
| CNAG_02537 | hypothetical protein CNAG_02537 | #N/A | 2.16 | #N/A | 2.15 |
| CNAG_00827 | ribose 5-phosphate isomerase | 2.75 | 2.16 | 4.89 | 1.20 |
| CNAG_00887 | hypothetical protein CNAG_00887 | 2.07 | 2.16 | 2.34 | 1.90 |
| CNAG_07547 | hypothetical protein CNAG_07547 | 1.97 | 2.15 | 1.59 | 2.65 |
| CNAG_00908 | hypothetical protein CNAG_00908 | 2.46 | 2.15 | 2.25 | 2.33 |
| CNAG_05425 | asparagine synthase (glutamine-hydrolyzing) | 0.62 | 2.15 | 0.82 | 1.61 |
| CNAG_00836 | 2-hydroxyacid dehydrogenase | 1.20 | 2.15 | 2.57 | 1.00 |
| CNAG_00751 | hypothetical protein CNAG_00751 | 0.85 | 2.15 | 1.42 | 1.27 |
| CNAG_05527 | senataxin | 0.85 | 2.14 | 1.75 | 1.04 |
| CNAG_02420 | hypothetical protein CNAG_02420 | 1.96 | 2.14 | 4.41 | 0.94 |
| CNAG_00716 | cytochrome c | 0.22 | 2.14 | 1.96 | 0.23 |
| CNAG_03044 | hypothetical protein CNAG_03044 | 0.56 | 2.14 | 1.66 | 0.71 |
| CNAG_03464 | laccase precursor | 1.69 | 2.12 | 3.16 | 1.12 |
| CNAG_05179 | ubiquinol-cytochrome c reductase core subunit 2 | 0.17 | 2.12 | 0.95 | 0.37 |
| CNAG_06827 | hypothetical protein CNAG_06827 | 2.67 | 2.12 | 4.05 | 1.38 |
| CNAG_07701 | hypothetical protein CNAG_07701 | 0.52 | 2.11 | 1.70 | 0.63 |
| CNAG_02872 | hypothetical protein CNAG_02872 | #N/A | 2.11 | #N/A | 1.84 |
| CNAG_04457 | hypothetical protein CNAG_04457 | 4.50 | 2.11 | 6.74 | 1.40 |
| CNAG_05184 | glycosyl transferase family 8 | 2.68 | 2.11 | 2.92 | 1.92 |
| CNAG_04077 | hypothetical protein CNAG_04077 | 2.04 | 2.11 | 3.37 | 1.27 |
| CNAG_02602 | flavonol synthase | 1.52 | 2.10 | 2.41 | 1.32 |
| CNAG_04898 | MFS transporter | 2.11 | 2.10 | 2.71 | 1.63 |
| CNAG_03669 | hypothetical protein CNAG_03669 | 1.05 | 2.10 | 1.55 | 1.41 |
| CNAG_06723 | succinate dehydrogenase (ubiquinone) membrane anchor subunit | 0.14 | 2.10 | 1.51 | 0.19 |
| CNAG_07443 | hypothetical protein CNAG_07443 | 2.43 | 2.10 | 3.59 | 1.40 |
| CNAG_04154 | hypothetical protein CNAG_04154 | 3.14 | 2.09 | 5.25 | 1.24 |
| CNAG_05420 | RNA polymerase II transcription factor | 0.85 | 2.09 | 1.14 | 1.54 |
| CNAG_03179 | hypothetical protein CNAG_03179 | 0.90 | 2.09 | 1.37 | 1.37 |
| CNAG_05429 | hypothetical protein CNAG_05429 | 1.00 | 2.09 | 1.91 | 1.08 |
| CNAG_04310 | hypothetical protein CNAG_04310 | 0.80 | 2.09 | 0.54 | 3.05 |
| CNAG_04053 | hypothetical protein CNAG_04053 | 1.53 | 2.08 | 3.70 | 0.85 |
| CNAG_07823 | solute carrier family 35 (UDP-galactose transporter) member B1 | 2.41 | 2.08 | 2.52 | 1.97 |
| CNAG_06186 | DUF895 domain membrane | 0.81 | 2.08 | 1.69 | 0.99 |
| CNAG_07432 | hypothetical protein CNAG_07432 | 1.38 | 2.08 | 1.97 | 1.44 |
| CNAG_03918 | hypothetical protein CNAG_03918 | 1.22 | 2.07 | 2.11 | 1.19 |
| CNAG_00662 | carboxymethylenebutenolidase | 1.16 | 2.07 | 2.46 | 0.97 |
| CNAG_02466 | hypothetical protein CNAG_02466 | 0.73 | 2.07 | 1.45 | 1.03 |
| CNAG_06617 | hypothetical protein CNAG_06617 | 0.33 | 2.07 | 1.37 | 0.49 |
| CNAG_01121 | hypothetical protein CNAG_01121 | 3.19 | 2.07 | 3.81 | 1.72 |
| CNAG_04616 | hypothetical protein CNAG_04616 | 2.19 | 2.07 | 3.96 | 1.14 |
| CNAG_06405 | hypothetical protein CNAG_06405 | 0.93 | 2.07 | 1.96 | 0.97 |
| CNAG_03195 | hypothetical protein CNAG_03195 | 1.69 | 2.06 | 2.79 | 1.24 |
| CNAG_06122 | glycerol-1-phosphatase | 0.54 | 2.06 | 1.55 | 0.70 |
| CNAG_06193 | CMGC RCK kinase | 0.71 | 2.06 | 1.07 | 1.35 |
| CNAG_01143 | hypothetical protein CNAG_01143 | 1.26 | 2.06 | 3.01 | 0.85 |
| CNAG_03216 | CAMK kinase | 0.38 | 2.06 | 1.13 | 0.69 |
| CNAG_03498 | variant 1 | 1.45 | 2.06 | 3.39 | 0.88 |
| CNAG_03548 | hypothetical protein CNAG_03548 | 1.58 | 2.05 | 2.22 | 1.45 |
| CNAG_06913 | L-serine ammonia-lyase | 2.68 | 2.05 | 3.35 | 1.63 |
| CNAG_04921 | hypothetical protein CNAG_04921 | 0.76 | 2.05 | 1.13 | 1.37 |
| CNAG_06138 | NADH dehydrogenase (ubiquinone) Fe-S 6 | 0.22 | 2.05 | 1.01 | 0.45 |
| CNAG_07757 | hypothetical protein CNAG_07757 | 1.30 | 2.05 | 1.99 | 1.32 |
| CNAG_06728 | kinesin | 0.83 | 2.05 | 1.73 | 0.98 |
| CNAG_03021 | hypothetical protein CNAG_03021 | 1.54 | 2.05 | 2.54 | 1.23 |
| CNAG_06758 | efflux protein | 1.44 | 2.05 | 4.98 | 0.59 |
| CNAG_06144 | E167 tumor | 0.74 | 2.05 | 2.26 | 0.66 |
| CNAG_02405 | hypothetical protein CNAG_02405 | 0.96 | 2.04 | 2.13 | 0.91 |
| CNAG_04535 | tartrate dehydrogenase | 0.64 | 2.04 | 0.80 | 1.63 |
| CNAG_03560 | hypothetical protein CNAG_03560 | 1.26 | 2.04 | 1.98 | 1.29 |
| CNAG_06082 | delayed-type hypersensitivity antigen - related | 0.09 | 2.04 | 0.34 | 0.52 |
| CNAG_02091 | nucleosome assembly I | 1.03 | 2.04 | 1.95 | 1.06 |
| CNAG_03061 | multiple drug resistance | 0.79 | 2.03 | 1.36 | 1.17 |
| CNAG_06239 | hypothetical protein CNAG_06239 | 1.39 | 2.03 | 2.70 | 1.04 |
| CNAG_03902 | transcriptional regulatory | 0.19 | 2.02 | 0.84 | 0.46 |
| CNAG_05345 | amino acid transporter | 1.73 | 2.02 | 2.34 | 1.49 |
| CNAG_06973 | hypothetical protein CNAG_06973 | 2.44 | 2.02 | 3.01 | 1.63 |
| CNAG_02478 | glycerol dehydrogenase | 1.16 | 2.02 | 1.94 | 1.19 |
| CNAG_07548 | cytoplasmic protein | 0.59 | 2.02 | 1.48 | 0.80 |
| CNAG_02011 | PQQ enzyme repeat | 1.16 | 2.02 | 2.69 | 0.86 |
| CNAG_01538 | ER to Golgi transport-related | 2.18 | 2.01 | 2.68 | 1.62 |
| CNAG_06209 | hypothetical protein CNAG_06209 | 1.30 | 2.01 | 2.26 | 1.14 |
|  |  |  |  |  |  |
|  |  |  |  |  |  |
|  |  |  |  |  |  |
|  |  |  |  |  |  |

The numbers in blue are those measurements where the log2 value is greater than or equal to 2; the numbers in green are log2 values of less than 0.5.
