# Supplementary material for: The Monothiol Glutaredoxin Grx4 Regulates Iron Homeostasis and Virulence in Cryptococcus neoformans
Source: mBio. 2018 Dec 4;9(6):e02377-18. doi: 10.1128/mBio.02377-18 (PMC6282196; doi:10.1128/mBio.02377-18)
Supplement: TABLE S2 [file mbo006184204st2.docx]

Table S2. Transcripts down-regulated in *grx4* under both low iron and high iron conditions

| **Name** | **Description** | **WT-L vs WT-H** | **grx4-L vs WT-L** | **grx4-H vs WT-H** | **grx4-L vs grx4-H** |
| --- | --- | --- | --- | --- | --- |
| CNAG_04865 | mannitol dehydrogenase | 2.34 | 0.00 | 0.01 | #N/A |
| CNAG_04516 | WSC domain-containing | 0.22 | 0.00 | 0.00 | #N/A |
| CNAG_07765 | hypothetical protein CNAG_07765 | 2.21 | 0.02 | 0.05 | 0.94 |
| CNAG_04864 | iron regulator 1 | 0.81 | 0.02 | 0.01 | 1.23 |
| CNAG_00052 | hypothetical protein CNAG_00052 | 2.13 | 0.03 | 0.06 | 1.03 |
| CNAG_00091 | hypothetical protein CNAG_00091 | 2.87 | 0.03 | 0.08 | 1.12 |
| CNAG_00834 | phosphatidylserine decarboxylase | 0.84 | 0.04 | 0.07 | 0.55 |
| CNAG_00539 | membrane transporter | 2.02 | 0.04 | 0.16 | 0.57 |
| CNAG_01052 | hypothetical protein CNAG_01052 | 1.63 | 0.05 | 0.09 | 0.81 |
| CNAG_04331 | hypothetical protein CNAG_04331 | 5.00 | 0.06 | 0.29 | 0.96 |
| CNAG_00848 | LEA domain | 3.50 | 0.06 | 0.21 | 0.95 |
| CNAG_03495 | hypothetical protein CNAG_03495 | 2.80 | 0.07 | 0.10 | 1.88 |
| CNAG_00984 | glucose and ribitol dehydrogenase | 4.12 | 0.07 | 0.16 | 1.69 |
| CNAG_03408 | hypothetical protein CNAG_03408 | 1.03 | 0.07 | 0.07 | 1.12 |
| CNAG_04105 | LEA domain | 1.95 | 0.07 | 0.15 | 0.98 |
| CNAG_07939 | hypothetical protein CNAG_07939 | 1.30 | 0.07 | 0.14 | 0.67 |
| CNAG_01047 | hypothetical protein CNAG_01047 | 2.49 | 0.08 | 0.17 | 1.09 |
| CNAG_03873 | hypothetical protein CNAG_03873 | 0.90 | 0.08 | 0.07 | 0.98 |
| CNAG_02706 | hypothetical protein CNAG_02706 | 2.48 | 0.08 | 0.12 | 1.64 |
| CNAG_03238 | dioxygenase subfamily | 4.31 | 0.08 | 0.31 | 1.16 |
| CNAG_02070 | hypothetical protein CNAG_02070 | 0.71 | 0.08 | 0.10 | 0.62 |
| CNAG_06267 | rds1 stress response related | 4.18 | 0.09 | 0.25 | 1.45 |
| CNAG_04903 | hypothetical protein CNAG_04903 | 1.83 | 0.09 | 0.24 | 0.66 |
| CNAG_02701 | hypothetical protein CNAG_02701 | 0.31 | 0.09 | 0.08 | 0.33 |
| CNAG_05683 | hypothetical protein CNAG_05683 | 1.19 | 0.09 | 0.11 | 1.02 |
| CNAG_07022 | hypothetical protein CNAG_07022 | 1.20 | 0.10 | 0.07 | #N/A |
| CNAG_02815 | glycerol-3-phosphate dehydrogenase | 1.05 | 0.10 | 0.15 | 0.69 |
| CNAG_04386 | hypothetical protein CNAG_04386 | 1.78 | 0.10 | 0.15 | 1.24 |
| CNAG_02950 | Grx4 family monothiol glutaredoxin | 1.72 | 0.11 | 0.15 | 1.21 |
| CNAG_04106 | hypothetical protein CNAG_04106 | 1.34 | 0.11 | 0.15 | 0.99 |
| CNAG_00093 | hypothetical protein CNAG_00093 | 1.11 | 0.11 | 0.12 | 0.99 |
| CNAG_03143 | 12 kda heat shock (glucose and lipid-regulated ) | 1.88 | 0.11 | 0.21 | 0.99 |
| CNAG_02129 | hypothetical protein CNAG_02129 | 1.16 | 0.11 | 0.13 | 1.00 |
| CNAG_01031 | hypothetical protein CNAG_01031 | 0.98 | 0.11 | 0.11 | 1.05 |
| CNAG_04459 | hypothetical protein CNAG_04459 | 6.16 | 0.12 | 1.64 | 0.43 |
| CNAG_06169 | (R,R)-butanediol dehydrogenase | 4.18 | 0.12 | 0.40 | 1.20 |
| CNAG_07319 | hypothetical protein CNAG_07319 | 1.07 | 0.12 | 0.10 | 1.21 |
| CNAG_06109 | hypothetical protein CNAG_06109 | 1.19 | 0.12 | 0.12 | 1.17 |
| CNAG_07868 | hypothetical protein CNAG_07868 | 0.90 | 0.13 | 0.13 | 0.84 |
| CNAG_03058 | hypothetical protein CNAG_03058 | 1.16 | 0.13 | 0.15 | 0.99 |
| CNAG_05607 | cellulase like glycosyl hydrolase | 1.36 | 0.13 | 0.10 | 1.68 |
| CNAG_00254 | NADH dehydrogenase | 1.00 | 0.13 | 0.19 | 0.67 |
| CNAG_07775 | hypothetical protein CNAG_07775 | 2.54 | 0.14 | 0.15 | 2.42 |
| CNAG_06668 | mitochondrial protein | 0.78 | 0.14 | 0.15 | 0.75 |
| CNAG_02263 | hypothetical protein CNAG_02263 | 1.00 | 0.14 | 0.18 | 0.80 |
| CNAG_01102 | oxidoreductase | 1.63 | 0.15 | 0.18 | 1.35 |
| CNAG_04076 | hypothetical protein CNAG_04076 | 1.52 | 0.15 | 0.20 | 1.13 |
| CNAG_02577 | inositolphosphorylceramide-B C-26 hydroxylase (IPC-B hydroxylase) | 2.96 | 0.15 | 0.46 | 0.96 |
| CNAG_04938 | hypothetical protein CNAG_04938 | 1.42 | 0.15 | 0.18 | 1.16 |
| CNAG_03728 | hypothetical protein CNAG_03728 | 1.14 | 0.15 | 0.17 | 0.97 |
| CNAG_05939 | hypothetical protein CNAG_05939 | 3.37 | 0.15 | 0.31 | 1.61 |
| CNAG_03771 | DNA binding Ncp1 | 0.87 | 0.16 | 0.14 | 1.00 |
| CNAG_04016 | hypothetical protein CNAG_04016 | 2.66 | 0.16 | 0.43 | 0.97 |
| CNAG_04163 | hypothetical protein CNAG_04163 | 1.01 | 0.16 | 0.11 | 1.50 |
| CNAG_00522 | C2 domain-containing | 1.14 | 0.16 | 0.13 | 1.40 |
| CNAG_04744 | mannose-6-phosphate class I | 2.57 | 0.16 | 0.32 | 1.29 |
| CNAG_00107 | hypothetical protein CNAG_00107 | 0.83 | 0.16 | 0.10 | 1.35 |
| CNAG_02398 | hypothetical protein CNAG_02398 | 1.48 | 0.17 | 0.27 | 0.92 |
| CNAG_03040 | transketolase | 1.67 | 0.17 | 0.18 | 1.54 |
| CNAG_06800 | hypothetical protein CNAG_06800 | 1.79 | 0.17 | 0.25 | 1.23 |
| CNAG_03492 | hypothetical protein CNAG_03492 | 0.83 | 0.17 | 0.22 | 0.64 |
| CNAG_01737 | methylsterol monooxygenase | 2.19 | 0.17 | 0.35 | 1.06 |
| CNAG_02942 | hypothetical protein CNAG_02942 | 2.86 | 0.17 | 0.53 | 0.92 |
| CNAG_04794 | spermine transporter | 1.77 | 0.17 | 0.43 | 0.70 |
| CNAG_01742 | water channel | 3.12 | 0.17 | 0.30 | 1.82 |
| CNAG_03142 | hypothetical protein CNAG_03142 | 1.34 | 0.17 | 0.22 | 1.04 |
| CNAG_01803 | hypothetical protein CNAG_01803 | 1.57 | 0.18 | 0.22 | 1.23 |
| CNAG_06201 | hypothetical protein CNAG_06201 | 1.33 | 0.18 | 0.20 | 1.14 |
| CNAG_00995 | meiotic recombination-related | 0.99 | 0.18 | 0.13 | 1.34 |
| CNAG_03563 | aspartate-tRNA(Asn) ligase | 1.86 | 0.18 | 0.27 | 1.25 |
| CNAG_07316 | alcohol dehydrogenase | 1.63 | 0.18 | 0.15 | 1.92 |
| CNAG_02335 | hypothetical protein CNAG_02335 | 0.80 | 0.18 | 0.20 | 0.72 |
| CNAG_01849 | hypothetical protein CNAG_01849 | 1.09 | 0.18 | 0.29 | 0.68 |
| CNAG_07516 | hypothetical protein CNAG_07516 | 1.08 | 0.18 | 0.48 | #N/A |
| CNAG_02877 | hypothetical protein CNAG_02877 | 1.27 | 0.19 | 0.18 | 1.33 |
| CNAG_03002 | hypothetical protein CNAG_03002 | 0.58 | 0.19 | 0.16 | 0.68 |
| CNAG_00047 | pyruvate dehydrogenase kinase | 2.03 | 0.19 | 0.33 | 1.16 |
| CNAG_02230 | phosphoketolase | 1.97 | 0.19 | 0.34 | 1.07 |
| CNAG_01341 | mannose-6-phosphate isomerase | 1.79 | 0.19 | 0.34 | 0.99 |
| CNAG_02705 | hypothetical protein CNAG_02705 | 1.32 | 0.19 | 0.23 | 1.10 |
| CNAG_04517 | hypothetical protein CNAG_04517 | 0.40 | 0.19 | 0.05 | 1.51 |
| CNAG_05356 | guanine nucleotide-binding subunit gamma | 2.24 | 0.19 | 1.50 | 0.29 |
| CNAG_04067 | haloacid type II | 3.01 | 0.20 | 0.43 | 1.37 |
| CNAG_06220 | allergen | 1.43 | 0.20 | 0.20 | 1.40 |
| CNAG_06207 | hypothetical protein CNAG_06207 | 0.45 | 0.20 | 0.19 | 0.46 |
| CNAG_04275 | metalloendopeptidase | 1.13 | 0.20 | 0.27 | 0.81 |
| CNAG_05608 | hypothetical protein CNAG_05608 | 1.81 | 0.20 | 0.36 | 0.99 |
| CNAG_02751 | short-chain dehydrogenase | 1.00 | 0.20 | 0.29 | 0.68 |
| CNAG_02069 | hypothetical protein CNAG_02069 | 0.86 | 0.20 | 0.20 | 0.86 |
| CNAG_01446 | hypothetical protein CNAG_01446 | 1.59 | 0.20 | 0.33 | 0.98 |
| CNAG_03794 | endoplasmic reticulum | 1.63 | 0.20 | 0.27 | 1.22 |
| CNAG_06843 | hypothetical protein CNAG_06843 | 2.96 | 0.20 | 0.50 | 1.20 |
| CNAG_06286 | hypothetical protein CNAG_06286 | 2.45 | 0.21 | 0.51 | 1.00 |
| CNAG_05167 | hypothetical protein CNAG_05167 | 1.15 | 0.21 | 0.24 | 1.02 |
| CNAG_06577 | hypothetical protein CNAG_06577 | 2.32 | 0.21 | 0.36 | 1.38 |
| CNAG_05031 | 3-oxoacid -transferase | 0.96 | 0.22 | 0.65 | 0.32 |
| CNAG_01368 | hypothetical protein CNAG_01368 | 0.31 | 0.22 | 0.10 | 0.69 |
| CNAG_03564 | hypothetical protein CNAG_03564 | 1.04 | 0.22 | 0.21 | 1.06 |
| CNAG_03881 | hypothetical protein CNAG_03881 | 1.19 | 0.23 | 0.20 | 1.32 |
| CNAG_06121 | hypothetical protein CNAG_06121 | 1.46 | 0.23 | 0.25 | 1.30 |
| CNAG_02768 | hypothetical protein CNAG_02768 | 2.97 | 0.23 | 0.32 | 2.11 |
| CNAG_01558 | chlorophyll synthesis pathway | 0.71 | 0.23 | 0.19 | 0.86 |
| CNAG_03566 | hypothetical protein CNAG_03566 | 1.08 | 0.23 | 0.19 | 1.28 |
| CNAG_00732 | hypothetical protein CNAG_00732 | 0.73 | 0.23 | 0.21 | 0.80 |
| CNAG_01042 | hypothetical protein CNAG_01042 | 1.82 | 0.23 | 0.45 | 0.94 |
| CNAG_00519 | lathosterol oxidase | 2.18 | 0.24 | 0.39 | 1.30 |
| CNAG_01585 | hypothetical protein CNAG_01585 | 1.26 | 0.24 | 0.38 | 0.77 |
| CNAG_07826 | hypothetical protein CNAG_07826 | 2.47 | 0.24 | 0.25 | 2.32 |
| CNAG_01736 | DASH complex subunit DAD4 | 2.37 | 0.24 | 0.47 | 1.18 |
| CNAG_02591 | hypothetical protein CNAG_02591 | 3.60 | 0.24 | 0.61 | 1.40 |
| CNAG_00057 | fructose-1,6-bisphosphatase I | 0.80 | 0.24 | 0.25 | 0.77 |
| CNAG_07391 | hypothetical protein CNAG_07391 | 2.05 | 0.24 | 0.60 | 0.80 |
| CNAG_06999 | hypothetical protein CNAG_06999 | 1.74 | 0.24 | 0.24 | 1.70 |
| CNAG_01751 | hypothetical protein CNAG_01751 | 1.31 | 0.24 | 0.26 | 1.19 |
| CNAG_03113 | trehalose synthase | 2.19 | 0.25 | 0.21 | 2.62 |
| CNAG_06302 | pathogenesis associated pep2 | 2.50 | 0.25 | 0.50 | 1.23 |
| CNAG_00854 | C-8 sterol isomerase | 2.46 | 0.25 | 0.57 | 1.08 |
| CNAG_07522 | hypothetical protein CNAG_07522 | 1.16 | 0.25 | 0.23 | 1.24 |
| CNAG_04160 | hypothetical protein CNAG_04160 | 1.49 | 0.25 | 0.36 | 1.05 |
| CNAG_02550 | hypothetical protein CNAG_02550 | 0.96 | 0.26 | 0.24 | 1.03 |
| CNAG_04015 | amino acid transporter | 1.35 | 0.26 | 0.34 | 1.03 |
| CNAG_06791 | hypothetical protein CNAG_06791 | 1.59 | 0.26 | 0.36 | 1.14 |
| CNAG_03937 | hypothetical protein CNAG_03937 | 1.46 | 0.26 | 0.36 | 1.04 |
| CNAG_03824 | solute carrier family 25 (mitochondrial phosphate transporter) member 3 | 1.19 | 0.26 | 0.31 | 1.00 |
| CNAG_01584 | hydrolase | 0.94 | 0.26 | 0.26 | 0.95 |
| CNAG_05994 | multidrug transporter | 1.04 | 0.27 | 0.28 | 0.98 |
| CNAG_06075 | hypothetical protein CNAG_06075 | 0.64 | 0.27 | 0.19 | 0.91 |
| CNAG_04804 | hypothetical protein CNAG_04804 | 2.45 | 0.27 | 0.40 | 1.64 |
| CNAG_00813 | hypothetical protein CNAG_00813 | 0.53 | 0.27 | 0.11 | 1.24 |
| CNAG_06381 | membrane Rsn1p | 1.29 | 0.27 | 0.46 | 0.76 |
| CNAG_03738 | pantetheine-phosphate adenylyltransferase | 1.39 | 0.28 | 0.46 | 0.82 |
| CNAG_04043 | hypothetical protein CNAG_04043 | 1.02 | 0.28 | 0.32 | 0.88 |
| CNAG_04926 | hypothetical protein CNAG_04926 | 1.26 | 0.28 | 0.33 | 1.05 |
| CNAG_00851 | hypothetical protein CNAG_00851 | 1.59 | 0.28 | 0.37 | 1.22 |
| CNAG_04687 | stearoyl- desaturase (delta-9 desaturase) | 1.14 | 0.29 | 0.30 | 1.08 |
| CNAG_06957 | hypothetical protein CNAG_06953 | 2.50 | 0.29 | 0.74 | 0.97 |
| CNAG_04070 | exonuclease | 1.39 | 0.30 | 0.31 | 1.32 |
| CNAG_05309 | hypothetical protein CNAG_05309 | 1.02 | 0.30 | 0.32 | 0.93 |
| CNAG_07629 | endopolyphosphatase | 1.26 | 0.30 | 0.44 | 0.84 |
| CNAG_04746 | hypothetical protein CNAG_04746 | 0.97 | 0.30 | 0.28 | 1.01 |
| CNAG_04351 | methylmalonate-semialdehyde dehydrogenase (acylating) | 2.10 | 0.30 | 0.48 | 1.31 |
| CNAG_05458 | endo-1,3(4)-beta-glucanase | 2.00 | 0.30 | 1.09 | 0.55 |
| CNAG_03572 | opsin 1 | 1.73 | 0.30 | 0.38 | 1.35 |
| CNAG_03595 | hypothetical protein CNAG_03595 | 0.93 | 0.30 | 0.38 | 0.73 |
| CNAG_02685 | hypothetical protein CNAG_02685 | 2.59 | 0.30 | 0.65 | 1.21 |
| CNAG_06238 | glutathione S-transferase | 4.01 | 0.31 | 0.84 | 1.45 |
| CNAG_01847 | hypothetical protein CNAG_01847 | 1.83 | 0.31 | 0.36 | 1.54 |
| CNAG_06064 | hypothetical protein CNAG_06064 | 1.00 | 0.31 | 0.32 | 0.95 |
| CNAG_04523 | glyceraldehyde-3-phosphate type I | 3.24 | 0.31 | 0.53 | 1.86 |
| CNAG_06771 | hypothetical protein CNAG_06771 | 1.46 | 0.31 | 0.43 | 1.02 |
| CNAG_06375 | hypothetical protein CNAG_06375 | 1.85 | 0.31 | 0.43 | 1.31 |
| CNAG_02896 | hydroxymethylglutaryl- synthase | 1.38 | 0.31 | 0.36 | 1.19 |
| CNAG_00465 | hypothetical protein CNAG_00465 | 1.99 | 0.31 | 0.41 | 1.50 |
| CNAG_00638 | GTPase | 1.64 | 0.31 | 0.45 | 1.12 |
| CNAG_00301 | hypothetical protein CNAG_00301 | 1.59 | 0.31 | 0.74 | 0.67 |
| CNAG_03617 | clampless 1 | 2.65 | 0.32 | 0.39 | 2.12 |
| CNAG_00485 | hypothetical protein CNAG_00485 | 1.77 | 0.32 | 0.55 | 1.01 |
| CNAG_06658 | rhomboid family membrane | 2.37 | 0.32 | 1.00 | 0.75 |
| CNAG_00850 | glycosyl hydrolase family 88 | 1.12 | 0.32 | 0.21 | 1.64 |
| CNAG_07338 | N-acyl-phosphatidylethanolamine-hydrolyzing phospholipase D | 1.29 | 0.32 | 0.38 | 1.08 |
| CNAG_02943 | cytoplasmic variant | 1.24 | 0.32 | 0.32 | 1.21 |
| CNAG_00873 | hypothetical protein CNAG_00873 | 2.92 | 0.32 | 0.81 | 1.15 |
| CNAG_06065 | inositol polyphosphate-5-phosphatase F | 1.27 | 0.32 | 0.32 | 1.27 |
| CNAG_05940 | hypothetical protein CNAG_05940 | 0.84 | 0.32 | 0.29 | 0.94 |
| CNAG_01924 | hypothetical protein CNAG_01924 | 1.15 | 0.32 | 0.30 | 1.22 |
| CNAG_04548 | hypothetical protein CNAG_04548 | 1.60 | 0.33 | 0.45 | 1.15 |
| CNAG_03083 | cupin domain-containing | 1.61 | 0.33 | 0.38 | 1.36 |
| CNAG_03007 | hypothetical protein CNAG_03007 | 2.34 | 0.33 | 0.96 | 0.79 |
| CNAG_07026 | hypothetical protein CNAG_07026 | 3.43 | 0.33 | 0.56 | 1.96 |
| CNAG_07728 | solute carrier family 39 (zinc transporter) member 1 2 3 | 1.97 | 0.33 | 0.60 | 1.08 |
| CNAG_02347 | hypothetical protein CNAG_02347 | 2.01 | 0.33 | 0.53 | 1.25 |
| CNAG_06256 | hypothetical protein CNAG_06256 | 1.02 | 0.33 | 0.32 | #N/A |
| CNAG_04789 | hypothetical protein CNAG_04789 | 1.45 | 0.33 | 0.40 | 1.19 |
| CNAG_03282 | hypothetical protein CNAG_03282 | 1.55 | 0.33 | 0.49 | 1.05 |
| CNAG_04274 | acyl- thioesterase II | 2.38 | 0.33 | 0.52 | 1.53 |
| CNAG_07493 | hypothetical protein CNAG_07493 | 0.56 | 0.33 | 0.18 | 1.02 |
| CNAG_03679 | hypothetical protein CNAG_03679 | 2.24 | 0.33 | 0.57 | 1.31 |
| CNAG_06139 | hypothetical protein CNAG_06139 | 1.19 | 0.33 | 0.26 | 1.54 |
| CNAG_03082 | cupin domain-containing | 1.90 | 0.34 | 0.57 | 1.12 |
| CNAG_06590 | hypothetical protein CNAG_06590 | 1.86 | 0.34 | 0.47 | 1.33 |
| CNAG_00766 | hypothetical protein CNAG_00766 | 2.31 | 0.34 | 0.46 | 1.69 |
| CNAG_01942 | hypothetical protein CNAG_01942 | 1.37 | 0.34 | 0.62 | 0.74 |
| CNAG_01007 | C2 domain-containing | 1.03 | 0.34 | 0.41 | 0.85 |
| CNAG_04361 | hypothetical protein CNAG_04361 | 1.12 | 0.34 | 0.29 | 1.32 |
| CNAG_05095 | pod-specific dehydrogenase SAC25 | 1.15 | 0.34 | 0.33 | 1.19 |
| CNAG_04208 | Machado-Joseph disease 1 (Ataxin-3) | 0.91 | 0.34 | 0.33 | 0.94 |
| CNAG_05331 | hypothetical protein CNAG_05331 | 0.60 | 0.34 | 0.14 | #N/A |
| CNAG_03215 | hypothetical protein CNAG_03215 | 1.65 | 0.34 | 0.54 | 1.05 |
| CNAG_03807 | E3 ubiquitin- ligase CCNP1IP1 | 1.79 | 0.34 | 0.56 | 1.09 |
| CNAG_04288 | Fe-S assembly co-chaperone | 1.78 | 0.34 | 0.60 | 1.01 |
| CNAG_06576 | allergen | 2.39 | 0.34 | 0.49 | 1.66 |
| CNAG_07790 | hypothetical protein CNAG_06523 | 1.20 | 0.34 | 0.55 | 0.75 |
| CNAG_07317 | hypothetical protein CNAG_07317 | 1.01 | 0.34 | 0.32 | 1.07 |
| CNAG_05388 | formamidopyrimidine-DNA glycosylase | 1.58 | 0.35 | 0.41 | 1.32 |
| CNAG_00079 | hypothetical protein CNAG_00079 | 0.67 | 0.35 | 0.20 | 1.17 |
| CNAG_00075 | hypothetical protein CNAG_00075 | 1.12 | 0.35 | 0.35 | 1.10 |
| CNAG_06694 | hydroxyisourate hydrolase | 1.99 | 0.35 | 0.44 | 1.57 |
| CNAG_03243 | 2-nitropropane dioxygenase | 2.07 | 0.35 | 0.51 | 1.40 |
| CNAG_03292 | hypothetical protein CNAG_03292 | 1.89 | 0.35 | 0.52 | 1.27 |
| CNAG_01874 | glutathione S-transferase | 1.00 | 0.35 | 0.35 | 0.99 |
| CNAG_07788 | hypothetical protein CNAG_07788 | 1.83 | 0.35 | 0.42 | 1.51 |
| CNAG_03875 | galactose-1-phosphate uridylyltransferase | 1.08 | 0.35 | 0.47 | 0.81 |
| CNAG_02053 | hypothetical protein CNAG_02053 | 1.85 | 0.35 | 0.45 | 1.42 |
| CNAG_05739 | hypothetical protein CNAG_05739 | 2.48 | 0.35 | 0.43 | 2.02 |
| CNAG_00921 | glutathione transferase | 1.42 | 0.35 | 0.33 | 1.53 |
| CNAG_03141 | hypothetical protein CNAG_03141 | 1.37 | 0.36 | 0.29 | 1.63 |
| CNAG_04833 | hypothetical protein CNAG_04833 | 1.18 | 0.36 | 0.32 | 1.31 |
| CNAG_04443 | hypothetical protein CNAG_04443 | 1.50 | 0.36 | 0.43 | 1.23 |
| CNAG_06955 | hypothetical protein CNAG_06955 | 1.64 | 0.36 | 0.59 | #N/A |
| CNAG_06245 | hypothetical protein CNAG_06245 | 2.03 | 0.36 | 0.28 | 2.62 |
| CNAG_05644 | 2-nitropropane dioxygenase | 2.02 | 0.36 | 0.59 | 1.22 |
| CNAG_05682 | hypothetical protein CNAG_05682 | 1.18 | 0.36 | 0.44 | 0.96 |
| CNAG_07492 | hypothetical protein CNAG_07492 | 0.67 | 0.36 | 0.20 | 1.22 |
| CNAG_00686 | hypothetical protein CNAG_00686 | 0.91 | 0.36 | 0.34 | 0.97 |
| CNAG_05022 | endoribonuclease l-psp | 2.28 | 0.36 | 0.53 | 1.54 |
| CNAG_07164 | hypothetical protein CNAG_07164 | 1.15 | 0.36 | 0.39 | 1.05 |
| CNAG_03454 | pria precursor | 0.85 | 0.36 | 0.27 | 1.13 |
| CNAG_05767 | regulation of carbohydrate metabolism-related | 1.86 | 0.37 | 0.61 | 1.10 |
| CNAG_02255 | BNR Asp-box repeat family | 1.19 | 0.37 | 0.31 | 1.40 |
| CNAG_00524 | acetyl- acyltransferase 2 | 1.71 | 0.37 | 0.41 | 1.53 |
| CNAG_00074 | integral to plasma membrane | 1.03 | 0.37 | 0.47 | 0.81 |
| CNAG_02667 | hypothetical protein CNAG_02667 | 1.93 | 0.37 | 0.69 | 1.02 |
| CNAG_04090 | bZip transcription factor | 0.84 | 0.37 | 0.35 | 0.90 |
| CNAG_07745 | mannitol-1-phosphate dehydrogenase | 0.75 | 0.37 | 0.23 | 1.19 |
| CNAG_06761 | siderophore-iron transporter Str1 | 2.29 | 0.37 | 0.89 | 0.96 |
| CNAG_05871 | hypothetical protein CNAG_05871 | 1.21 | 0.38 | 0.39 | 1.17 |
| CNAG_04322 | hypothetical protein CNAG_04322 | 1.15 | 0.38 | 0.47 | 0.92 |
| CNAG_03759 | conidiation-specific 6 | 2.66 | 0.38 | 0.59 | 1.68 |
| CNAG_00961 | hypothetical protein CNAG_00961 | 1.77 | 0.38 | 0.63 | 1.05 |
| CNAG_06453 | benzodiazapine receptor | 1.37 | 0.38 | 0.45 | 1.15 |
| CNAG_06884 | hypothetical protein CNAG_06884 | 1.01 | 0.38 | 0.32 | 1.20 |
| CNAG_04781 | hypothetical protein, variant 2 | 3.34 | 0.38 | 1.04 | 1.21 |
| CNAG_01369 | hypothetical protein CNAG_01369 | 1.54 | 0.38 | 0.46 | 1.28 |
| CNAG_06050 | UDP-glucose 4-epimerase | 2.53 | 0.38 | 0.93 | 1.03 |
| CNAG_06413 | hypothetical protein CNAG_06413 | 1.05 | 0.39 | 0.43 | 0.93 |
| CNAG_03213 | UV damage endonuclease | 1.46 | 0.39 | 0.45 | 1.24 |
| CNAG_05686 | hypothetical protein CNAG_05686 | 1.45 | 0.39 | 0.45 | 1.23 |
| CNAG_06074 | cytoplasmic variant | 2.27 | 0.39 | 0.53 | 1.64 |
| CNAG_03398 | solute carrier family 39 (zinc transporter) member 1 2 3 | 1.77 | 0.39 | 0.70 | 0.98 |
| CNAG_02864 | hypothetical protein CNAG_02864 | 1.87 | 0.39 | 1.22 | 0.59 |
| CNAG_03268 | hypothetical protein CNAG_03268 | 1.45 | 0.39 | 0.42 | 1.33 |
| CNAG_06431 | acyl- oxidase | 1.72 | 0.39 | 0.41 | 1.65 |
| CNAG_04737 | hypothetical protein CNAG_04737 | 1.54 | 0.39 | 0.52 | 1.16 |
| CNAG_07955 | hypothetical protein CNAG_07955 | 1.22 | 0.39 | 0.42 | 1.14 |
| CNAG_00729 | hypothetical protein CNAG_00729 | 1.97 | 0.39 | #N/A | #N/A |
| CNAG_02182 | D-lactaldehyde dehydrogenase | 2.42 | 0.39 | 0.78 | 1.21 |
| CNAG_03828 | aromatic amino acid aminotransferase I | 1.40 | 0.40 | 0.34 | 1.62 |
| CNAG_00521 | hypothetical protein CNAG_00521 | 1.46 | 0.40 | 0.36 | 1.60 |
| CNAG_03685 | hypothetical protein CNAG_03685 | 1.26 | 0.40 | 0.58 | 0.86 |
| CNAG_04587 | hypothetical protein CNAG_04587 | 0.59 | 0.40 | 0.22 | 1.07 |
| CNAG_07851 | isocitrate NAD-dependent | 1.13 | 0.40 | 0.56 | 0.80 |
| CNAG_02006 | N-terminal asparagine amidohydrolase | 1.43 | 0.40 | 0.50 | 1.13 |
| CNAG_06602 | cysteine-type peptidase | 2.37 | 0.40 | 0.63 | 1.48 |
| CNAG_07557 | ATP-binding cassette transporter | 0.62 | 0.40 | 0.36 | 0.69 |
| CNAG_02926 | hypothetical protein CNAG_02926 | 1.28 | 0.40 | 0.70 | 0.72 |
| CNAG_01787 | hypothetical protein CNAG_01787 | 2.29 | 0.40 | 0.78 | 1.17 |
| CNAG_02584 | serine threonine- phosphatase 2A activator 1 | 1.72 | 0.40 | 0.67 | 1.02 |
| CNAG_00866 | transketolase | 1.47 | 0.40 | 0.68 | 0.86 |
| CNAG_01386 | phosphatidylinositol class P | 1.61 | 0.40 | 0.58 | 1.11 |
| CNAG_02348 | hypothetical protein CNAG_02348 | 1.05 | 0.40 | 0.67 | 0.63 |
| CNAG_04970 | hypothetical protein CNAG_04970 | 1.13 | 0.40 | 0.40 | 1.14 |
| CNAG_03705 | hypothetical protein CNAG_03705 | 1.17 | 0.41 | 0.39 | 1.22 |
| CNAG_02655 | hypothetical protein CNAG_02655 | 1.03 | 0.41 | 0.35 | 1.19 |
| CNAG_01155 | glycerol kinase | 0.58 | 0.41 | 0.27 | 0.86 |
| CNAG_05732 | hypothetical protein CNAG_05732 | 1.49 | 0.41 | 0.56 | 1.08 |
| CNAG_03239 | hypothetical protein CNAG_03239 | 1.30 | 0.41 | 0.63 | 0.84 |
| CNAG_02753 | endoplasmic reticulum | 1.19 | 0.41 | 0.43 | 1.14 |
| CNAG_01750 | heat shock | 0.32 | 0.41 | 0.15 | 0.85 |
| CNAG_02297 | hypothetical protein CNAG_02297 | 3.96 | 0.41 | 1.07 | 1.52 |
| CNAG_03178 | hypothetical protein CNAG_03178 | 1.09 | 0.41 | 0.45 | 0.99 |
| CNAG_03905 | cell growth-related | 0.79 | 0.41 | 0.39 | 0.82 |
| CNAG_00776 | immunoreactive manno MP88 | 1.38 | 0.41 | 0.82 | 0.70 |
| CNAG_06448 | cystathionine gamma-lyase | 2.31 | 0.41 | 0.73 | 1.31 |
| CNAG_01946 | allantoate permease | 1.08 | 0.42 | 0.60 | 0.75 |
| CNAG_07303 | hypothetical protein CNAG_07303 | 1.16 | 0.42 | 0.60 | 0.80 |
| CNAG_06922 | lipid metabolism-related | 1.33 | 0.42 | 0.65 | 0.84 |
| CNAG_04112 | oxidoreductase | 1.11 | 0.42 | 0.32 | 1.43 |
| CNAG_02427 | hypothetical protein CNAG_02427 | 2.48 | 0.42 | 0.88 | 1.17 |
| CNAG_04886 | hypothetical protein CNAG_04886 | 1.38 | 0.42 | 0.51 | 1.12 |
| CNAG_01043 | hypothetical protein CNAG_01043 | 1.25 | 0.42 | 0.52 | 0.99 |
| CNAG_07591 | mitochondrial metalloendopeptidase OMA1 | 1.00 | 0.42 | 0.48 | 0.88 |
| CNAG_07639 | lipase 2 | 1.47 | 0.42 | 0.61 | 1.01 |
| CNAG_02211 | hypothetical protein CNAG_02211 | 1.53 | 0.42 | 0.51 | 1.27 |
| CNAG_04098 | xenobiotic-transporting ATPase | 1.53 | 0.42 | 0.44 | 1.45 |
| CNAG_06616 | hypothetical protein CNAG_06616 | 1.23 | 0.42 | 0.40 | 1.28 |
| CNAG_05652 | cytoplasmic protein | 1.22 | 0.42 | 0.65 | 0.79 |
| CNAG_00036 | Sec14 cytosolic factor | 1.05 | 0.43 | 0.44 | 1.01 |
| CNAG_06993 | hypothetical protein CNAG_06993 | 1.35 | 0.43 | 0.99 | 0.58 |
| CNAG_00130 | serine threonine- kinase | 1.21 | 0.43 | 0.51 | 1.02 |
| CNAG_04152 | phosphatase methylesterase 1 | 1.02 | 0.43 | 0.37 | 1.16 |
| CNAG_03509 | pyruvate dehydrogenase x mitochondrial precursor | 0.86 | 0.43 | 0.46 | 0.80 |
| CNAG_07642 | gag-pol poly | 1.27 | 0.43 | 0.63 | 0.85 |
| CNAG_04934 | hypothetical protein CNAG_04934 | 1.44 | 0.43 | 0.64 | 0.95 |
| CNAG_07940 | hypothetical protein CNAG_07940 | 1.21 | 0.43 | 0.49 | 1.06 |
| CNAG_00121 | glycerol-3-phosphate dehydrogenase (NAD(+)) | 1.17 | 0.43 | 0.46 | 1.10 |
| CNAG_00480 | hypothetical protein CNAG_00480 | 1.70 | 0.43 | 0.59 | 1.23 |
| CNAG_01821 | hypothetical protein CNAG_01821 | 1.27 | 0.43 | 0.63 | 0.87 |
| CNAG_07954 | hypothetical protein CNAG_07954 | 1.68 | 0.43 | 0.92 | 0.78 |
| CNAG_05658 | L-arabinitol 4-dehydrogenase | 0.50 | 0.43 | 0.18 | 1.20 |
| CNAG_01129 | lanosterol synthase | 1.66 | 0.43 | 0.54 | 1.32 |
| CNAG_07586 | hypothetical protein CNAG_07586 | 2.02 | 0.43 | 0.98 | 0.88 |
| CNAG_02953 | tuberin | 1.31 | 0.43 | 0.51 | 1.11 |
| CNAG_07004 | dihydrolipoyl dehydrogenase | 0.81 | 0.43 | 0.41 | 0.85 |
| CNAG_02883 | rho family | 1.50 | 0.43 | 0.62 | 1.04 |
| CNAG_04849 | vacuolar protein | 1.10 | 0.44 | 0.61 | 0.79 |
| CNAG_06104 | hypothetical protein CNAG_06104 | 0.81 | 0.44 | 0.54 | 0.65 |
| CNAG_05333 | hypothetical protein CNAG_05333 | 2.40 | 0.44 | 0.78 | 1.34 |
| CNAG_07923 | hypothetical protein CNAG_07923 | 4.59 | 0.44 | #N/A | #N/A |
| CNAG_04879 | glycogen debranching enzyme | 1.65 | 0.44 | 0.52 | 1.37 |
| CNAG_05599 | hypothetical protein CNAG_05599 | 1.43 | 0.44 | 0.48 | 1.30 |
| CNAG_07641 | monosaccharide transporter | 1.12 | 0.44 | 0.92 | 0.53 |
| CNAG_04091 | hypothetical protein CNAG_04091 | 1.31 | 0.44 | 0.59 | 0.97 |
| CNAG_05638 | hypothetical protein CNAG_05638 | 0.60 | 0.44 | 0.29 | 0.91 |
| CNAG_05864 | hypothetical protein CNAG_05864 | 1.73 | 0.44 | 0.64 | 1.19 |
| CNAG_06290 | high-affinity glucose transporter SNF3 | 1.23 | 0.44 | 0.53 | 1.01 |
| CNAG_03565 | plasma-membrane proton-efflux P-type ATPase | 1.70 | 0.44 | 0.85 | 0.87 |
| CNAG_02974 | voltage-dependent anion channel 2 | 0.63 | 0.44 | 0.31 | 0.91 |
| CNAG_06962 | DNA ligase 3 -phosphoesterase domain-containing | 2.02 | 0.44 | 0.66 | 1.35 |
| CNAG_01492 | hypothetical protein CNAG_01492 | 1.15 | 0.44 | 0.45 | 1.13 |
| CNAG_05971 | hypothetical protein CNAG_05971 | 2.11 | 0.44 | 0.76 | 1.22 |
| CNAG_04084 | hypothetical protein CNAG_04084 | 2.06 | 0.44 | 0.92 | 0.98 |
| CNAG_04807 | hypothetical protein CNAG_04807 | 1.29 | 0.44 | 0.51 | 1.12 |
| CNAG_01299 | hypothetical protein CNAG_01299 | 1.71 | 0.45 | 0.82 | 0.92 |
| CNAG_02005 | hypothetical protein CNAG_02005 | 0.71 | 0.45 | 0.25 | 1.24 |
| CNAG_02814 | glycerol-3-phosphate dehydrogenase | 0.73 | 0.45 | 0.30 | 1.08 |
| CNAG_07975 | hypothetical protein CNAG_07975 | 1.95 | 0.45 | 0.80 | 1.08 |
| CNAG_07574 | hypothetical protein CNAG_07574 | 2.03 | 0.45 | 0.72 | 1.25 |
| CNAG_06232 | transcription factor C subunit 7 | 1.47 | 0.45 | 0.53 | 1.24 |
| CNAG_04946 | hypothetical protein CNAG_04946 | 1.33 | 0.45 | 0.48 | 1.22 |
| CNAG_00424 | choline-phosphate cytidylyltransferase | 0.80 | 0.45 | 0.36 | 1.00 |
| CNAG_01753 | hypothetical protein CNAG_01753 | 1.28 | 0.45 | 0.46 | 1.25 |
| CNAG_01933 | hypothetical protein CNAG_01933 | 3.13 | 0.45 | #N/A | #N/A |
| CNAG_03609 | hypothetical protein CNAG_03609 | 1.15 | 0.45 | 0.40 | 1.30 |
| CNAG_06396 | hypothetical protein CNAG_06396 | 3.51 | 0.45 | 1.04 | 1.52 |
| CNAG_03187 | protoporphyrinogen oxidase | 0.92 | 0.45 | 0.45 | 0.91 |
| CNAG_05443 | hypothetical protein CNAG_05443 | 1.00 | 0.45 | 0.60 | 0.75 |
| CNAG_03883 | hypothetical protein CNAG_03883 | 1.25 | 0.46 | 0.62 | 0.91 |
| CNAG_07406 | pheromone alpha | 1.06 | 0.46 | 0.61 | 0.78 |
| CNAG_06781 | hypothetical protein CNAG_06781 | 1.16 | 0.46 | 0.51 | 1.02 |
| CNAG_05677 | phospholipase | 1.45 | 0.46 | 0.86 | 0.76 |
| CNAG_02830 | delta24(24(1))-sterol reductase | 1.82 | 0.46 | 0.94 | 0.88 |
| CNAG_02041 | hypothetical protein CNAG_02041 | 1.05 | 0.46 | 0.39 | 1.22 |
| CNAG_03041 | hypothetical protein CNAG_03041 | 1.74 | 0.46 | 0.69 | 1.14 |
| CNAG_00863 | flavin-containing monooxygenase | 1.74 | 0.46 | 0.55 | 1.46 |
| CNAG_03047 | hypothetical protein CNAG_03047 | 1.37 | 0.46 | 0.83 | 0.76 |
| CNAG_00941 | hypothetical protein CNAG_00941 | 1.27 | 0.46 | 0.63 | 0.92 |
| CNAG_03991 | integral membrane | 1.19 | 0.46 | 0.41 | 1.33 |
| CNAG_04750 | hypothetical protein CNAG_04750 | 1.13 | 0.46 | 0.43 | 1.20 |
| CNAG_03946 | galactokinase | 1.25 | 0.46 | 0.56 | 1.03 |
| CNAG_01588 | plasma membrane proteolipid 3 | 1.35 | 0.47 | 0.73 | 0.86 |
| CNAG_07043 | hypothetical protein CNAG_07043 | 2.10 | 0.47 | 0.75 | 1.29 |
| CNAG_05684 | hypothetical protein CNAG_05684 | 1.03 | 0.47 | 0.54 | 0.87 |
| CNAG_07363 | isocitrate NAD-dependent | 0.85 | 0.47 | 0.50 | 0.78 |
| CNAG_05238 | hypothetical protein CNAG_05238 | 1.39 | 0.47 | 0.52 | 1.25 |
| CNAG_02857 | response to drug-related | 0.74 | 0.47 | 0.47 | 0.74 |
| CNAG_07347 | heat shock | 0.15 | 0.47 | 0.13 | 0.53 |
| CNAG_01984 | transaldolase | 0.97 | 0.47 | 0.44 | 1.03 |
| CNAG_05168 | hypothetical protein CNAG_05168 | 1.38 | 0.47 | 0.77 | 0.85 |
| CNAG_00488 | hypothetical protein CNAG_00488 | 2.31 | 0.47 | 0.91 | 1.18 |
| CNAG_07927 | hypothetical protein, variant | 1.27 | 0.47 | 0.72 | 0.83 |
| CNAG_07032 | peroxiredoxin atypical 2-Cys peroxiredoxin | 1.62 | 0.47 | 0.58 | 1.30 |
| CNAG_07482 | SCF-associated factor 1 | 1.76 | 0.48 | 0.55 | 1.50 |
| CNAG_06497 | microsomal epoxide hydrolase | 1.89 | 0.48 | 0.59 | 1.52 |
| CNAG_06764 | short-chain dehydrogenase | 1.70 | 0.48 | 0.61 | 1.31 |
| CNAG_03631 | hypothetical protein CNAG_03631 | 0.96 | 0.48 | 0.45 | 1.01 |
| CNAG_05444 | NADPH dehydrogenase | 1.19 | 0.48 | 0.88 | 0.64 |
| CNAG_05785 | hypothetical protein CNAG_05785 | 2.09 | 0.48 | 0.81 | 1.22 |
| CNAG_01745 | glycerol-3-phosphate dehydrogenase (NAD+) | 0.76 | 0.48 | 0.39 | 0.92 |
| CNAG_04387 | thioredoxin 4A | 0.88 | 0.48 | 0.34 | 1.25 |
| CNAG_01481 | hypothetical protein CNAG_01481 | 2.33 | 0.48 | 0.69 | 1.62 |
| CNAG_00164 | hypothetical protein CNAG_00164 | 1.68 | 0.48 | 0.58 | 1.38 |
| CNAG_04659 | pyruvate decarboxylase | 0.45 | 0.48 | 0.23 | 0.92 |
| CNAG_06493 | hypothetical protein CNAG_06493 | 2.14 | 0.48 | 0.71 | 1.44 |
| CNAG_00125 | hypothetical protein CNAG_00125 | 0.70 | 0.48 | 0.34 | 0.98 |
| CNAG_01417 | hypothetical protein CNAG_01417 | 1.66 | 0.49 | 0.48 | 1.66 |
| CNAG_06244 | hypothetical protein CNAG_06244 | 1.19 | 0.49 | 0.53 | 1.08 |
| CNAG_06432 | acetate kinase | 1.21 | 0.49 | 0.51 | 1.16 |
| CNAG_05396 | LRP16 family | 1.85 | 0.49 | 0.54 | 1.65 |
| CNAG_00516 | peroxisome targeting signal receptor | 1.25 | 0.49 | 0.46 | 1.32 |
| CNAG_07786 | hypothetical protein CNAG_07786 | 1.13 | 0.49 | 0.39 | #N/A |
| CNAG_05104 | CAMK kinase | 1.98 | 0.49 | 0.82 | 1.17 |
| CNAG_05201 | DNA mismatch repair MSH4 | 2.86 | 0.49 | 0.87 | 1.61 |
| CNAG_02392 | hypothetical protein CNAG_02392 | 1.69 | 0.49 | 0.65 | 1.26 |
| CNAG_06594 | oxysterol binding | 1.00 | 0.49 | 0.53 | 0.93 |
| CNAG_04958 | ubiquitin fusion degradation 1 | 1.06 | 0.49 | 0.51 | 1.03 |
| CNAG_03517 | 64 kDa mitochondrial NADH dehydrogenase | 1.89 | 0.49 | 0.64 | 1.45 |
| CNAG_01769 | mitochondrial inner membrane | 0.77 | 0.49 | 0.47 | 0.80 |
| CNAG_02282 | carboxypeptidase A4 | 1.20 | 0.49 | 0.52 | 1.13 |
| CNAG_01691 | hypothetical protein, variant | 1.03 | 0.49 | 0.50 | 1.00 |
| CNAG_03769 | hexokinase | 0.74 | 0.49 | 0.39 | 0.93 |
| CNAG_04515 | hypothetical protein CNAG_04515 | 1.23 | 0.49 | 0.55 | 1.09 |
| CNAG_02552 | variant 1 | 1.97 | 0.50 | 0.61 | 1.58 |
| CNAG_06868 | enolase 1 | 1.63 | 0.50 | 0.77 | 1.04 |
| CNAG_01506 | hypothetical protein CNAG_01506 | 2.93 | 0.50 | 1.89 | 0.76 |
| CNAG_00050 | hypothetical protein CNAG_00050 | 1.06 | 0.50 | 0.52 | 1.01 |
| CNAG_06863 | hypothetical protein CNAG_06863 | 2.10 | 0.50 | 0.55 | 1.88 |
| CNAG_04248 | ubiquitin thioesterase OTUB1 | 1.18 | 0.50 | 0.42 | 1.39 |
| CNAG_03997 | hypothetical protein CNAG_03997 | 1.53 | 0.50 | 0.98 | 0.77 |
| CNAG_00919 | carboxypeptidase D | 0.99 | 0.50 | 0.59 | 0.82 |
| CNAG_01014 | hypothetical protein CNAG_01014 | 0.90 | 0.50 | 0.48 | 0.92 |
| CNAG_06704 | hypothetical protein CNAG_06704 | 0.82 | 0.50 | 0.45 | 0.91 |
|  |  |  |  |  |  |
|  |  |  |  |  |  |
|  |  |  |  |  |  |
|  |  |  |  |  |  |

The numbers in blue are those measurements where the log2 value is greater than or equal to 2; the numbers in yellow are log2 values of less than 0.5.
