# Supplementary material for: The Monothiol Glutaredoxin Grx4 Regulates Iron Homeostasis and Virulence in Cryptococcus neoformans
Source: mBio. 2018 Dec 4;9(6):e02377-18. doi: 10.1128/mBio.02377-18 (PMC6282196; doi:10.1128/mBio.02377-18)
Supplement: TABLE S3 [file mbo006184204st3.docx]

| **Table S3. Primers used for strain construction and qPCR** | |  |
| --- | --- | --- |
| **Strain construction** | Primer name | Sequence (5'-3') |
| *grx4* mutant | 1-GRX4 | GCGAGATGGGCACATCCTTTATGTGACC |
|  | 2-GRX4 | TGCTCCAGTGCATCCCATCGTGCAGGCC |
|  | 3-GRX4 | GGTTCGGCTCTGGCTAATGAAcaggaaacagctatgaccatg |
|  | 4-GRX4 | catggtcatagctgtttcctgTTCATTAGCCAGAGCCGAACC |
|  | 5-GRX4 | cactggccgtcgttttacaacCGGGTCAGGTCGAAGATTACC |
|  | 6-GRX4 | GGTAATCTTCGACCTGACCCGgttgtaaaacgacggccagtg |
|  | 7-GRX4 | AGGACGTCACTCTCCGAGCTTAACAACC |
|  | 8-GRX4 | GAAGCGTGCGGGGGAGAGGTGAAAGACC |
|  | 9-GRX4 | TCACAATCACCACCACGCGCC |
|  | 10-GRX4 | TAGCGTGCGTTTTTCAGCGTC |
| *grx4* complementation | SS-A | GTTTCTACATCTCTTCCGTGTTAATACAGAGACCTGACCCGATGACGCTGAAAAACGCACG |
|  | SS-B | CCGCGACGTGGTTCGGCTCTGGCTAATGAATGGGCGCGTGGTGGTGATTGTGATGGGGAT |
|  | SS-7 | AGGACGTCACTCTCCGAGCTTAACAACC |
|  | SS-C | ATGGAATGCGTGAGATCG |
|  | SS-1 | GCGAGATGGGCACATCCTTTATGTGACC |
|  | SS-D | CTGCGAGGATGTGAGCTG |
|  | SS-E | CAGCTCACATCCTCGCAG |
| Grx4-mCherry | Grx3mCherry-P1F | CGCCGACGCTTCCCTCCTTCACTCTCTC |
|  | Grx3mCherry-P1R | TGTTATCCTCCTCGCCCTTGCTCACCACCTCCGTCTTGCCCTCCTCGATAG |
|  | Grx3mCherry-P2F | CTATCGAGGAGGGCAAGACGGAGGTGGTGAGCAAGGGCGAGGAGGATAACA |
|  | Grx3mCherry-P2R | ACTCCTTTCCCGCTCCAAGGCGCTCGCCCAAGCTTGGTACCGAGCTCGGATC |
|  | Grx3mCherry-P3F | GATCCGAGCTCGGTACCAAGCTTGGGCGAGCGCCTTGGAGCGGGAAAGGAGT |
|  | Grx3mCherry-P3R | CTCAGTTACTCGCCAACCCCATCCA |
| Cir1-GFP | Cir1-GFP-P1F | CCAATGTCCTTTCTCCTCCACGAC |
|  | Cir1-GFP-P1R | GTGAACAGCTCCTCGCCCTTGCTCACACTCCTAACGTCAAAACTCCACA |
|  | Cir1-GFP-P2F | TGTGGAGTTTTGACGTTAGGAGTGTGAGCAAGGGCGAGGAGCTGTTCAC |
|  | Cir1-GFP-P3R | AGTCTGTACCAACTTCCAACTCCAcacgacgttgtaaaacgacggccag |
|  | Cir1-GFP-P5F | ctggccgtcgttttacaacgtcgtgTGGAGTTGGAAGTTGGTACAGACT |
|  | Cir1-GFP-P5R | TGATTGTCCGATTTTCGAACACTTCC |

**Primers for qPCR**

| Primers | Sequence |
| --- | --- |
| Fre3_F | TCGGTGTCTGCGGTCCAT |
| Fre3_R | CCTCCCTGACACCCTTTCG |
| LAC1_F | CCCCGAGTCTTGGACGAAT |
| LAC1_R | GCGGGTCCAAATGCATTG |
| Cir1_F | GATCGCGAGCATCGTCCTT |
| Cir1_R | AACCGTTACCCATGCTGTTCTC |
| TEF2_F | CCTTCCTTGCCCTCTTCTCAT |
| TEF2_R | AGCGACGACAGGGACAATG |
