# Supplementary material for: The Monothiol Glutaredoxin Grx4 Regulates Iron Homeostasis and Virulence in Cryptococcus neoformans
Source: mBio. 2018 Dec 4;9(6):e02377-18. doi: 10.1128/mBio.02377-18 (PMC6282196; doi:10.1128/mBio.02377-18)
Supplement: TABLE S4 [file mbo006184204st4.docx]

| **Table S4. Bioproject and biosample accession numbers for the RNASeq data.** | | | |
| --- | --- | --- | --- |
| **bioproject_accession** | **biosample_accession** | **sample_name** | **library_ID** |
| PRJNA478320 | SAMN09510544 | WT | WT_lowFe_rep1 |
| PRJNA478320 | SAMN09510544 | WT | WT_lowFe_rep2 |
| PRJNA478320 | SAMN09510544 | WT | WT_lowFe_rep3 |
| PRJNA478320 | SAMN09510544 | WT | WT_highFe_rep1 |
| PRJNA478320 | SAMN09510544 | WT | WT_highFe_rep2 |
| PRJNA478320 | SAMN09510544 | WT | WT_highFe_rep3 |
| PRJNA478320 | SAMN09510545 | grx4 | grx4_lowFe_rep1 |
| PRJNA478320 | SAMN09510545 | grx4 | grx4_lowFe_rep2 |
| PRJNA478320 | SAMN09510545 | grx4 | grx4_lowFe_rep3 |
| PRJNA478320 | SAMN09510545 | grx4 | grx4_highFe_rep1 |
| PRJNA478320 | SAMN09510545 | grx4 | grx4_highFe_rep2 |
| PRJNA478320 | SAMN09510545 | grx4 | grx4_highFe_rep3 |
